# Effectiveness and Cost-Effectiveness of Survivorship Care for Survivors of Hodgkin Lymphoma (INSIGHT Study): Protocol for a Multicenter Retrospective Cohort Study With a Quasi-Experimental Design

**DOI:** 10.2196/55601

**Published:** 2024-04-18

**Authors:** Eline M J Lammers, Josée M Zijlstra, Valesca P Retèl, Berthe M P Aleman, Flora E van Leeuwen, Annelies Nijdam, BETER consortium

**Affiliations:** 1 Department of Epidemiology The Netherlands Cancer Institute Amsterdam Netherlands; 2 Department of Hematology Amsterdam UMC, location Vrije Universiteit, Cancer Center Amsterdam Netherlands; 3 Department of Health Technology Assessment The Netherlands Cancer Institute Amsterdam Netherlands; 4 Department of Radiation Oncology The Netherlands Cancer Institute Amsterdam Netherlands; 5 See Acknowledgments

**Keywords:** research design, Hodgkin lymphoma, late effects of cancer treatment, survivorship care, screening, cost-effectiveness analysis

## Abstract

**Background:**

Hodgkin lymphoma (HL) occurs at young ages, with the highest incidence between 20 and 40 years. While cure rates have improved to 80%-90% over the past decades, survivors of HL are at substantial risk of late treatment–related complications, such as cardiovascular diseases, breast cancer, severe infections, and hypothyroidism. To reduce morbidity and mortality from late treatment effects, the Dutch Better care after lymphoma, Evaluation of long-term Treatment Effects and screening Recommendations (BETER) consortium developed a survivorship care program for 5-year survivors of HL that includes risk-based screening for and treatment of (risk factors for) late adverse events. Even though several cancer survivorship care programs have been established worldwide, there is a lack of knowledge about their effectiveness in clinical practice.

**Objective:**

The Improving Nationwide Survivorship care Infrastructure and Guidelines after Hodgkin lymphoma Treatment (INSIGHT) study evaluates whether Dutch BETER survivorship care for survivors of HL decreases survivors’ burden of disease from late adverse events after HL treatment and associated health care costs and improves their quality of life.

**Methods:**

The INSIGHT study is a multicenter retrospective cohort study with a quasi-experimental design and prospective follow-up, embedded in the national BETER survivorship care infrastructure. The first BETER clinics started in 2013-2016 and several other centers started or will start BETER clinics in 2019-2024. This allows us to compare survivors who did and those who did not receive BETER survivorship care in the last decade. Survivors in the intervention group are matched to controls (n=450 per group) based on sex, age at diagnosis (±5 years), age in 2013 (±5 years), and treatment characteristics. The primary outcome is the burden of disease in disability-adjusted life years from cardiovascular disease, breast cancer, severe infections, and hypothyroidism. In a cost-effectiveness analysis, we will assess the cost of BETER survivorship care per averted or gained disability-adjusted life year and quality-adjusted life year. Secondary outcomes are BETER clinic attendance, adherence to screening guidelines, and knowledge and distress about late effects among survivors of HL. Study data are collected from a survivor survey, a general practitioner survey, medical records, and through linkages with national disease registries.

**Results:**

The study was funded in November 2020 and approved by the institutional review board of the Netherlands Cancer Institute in July 2021. We expect to finalize recruitment by October 2024, data collection by early 2025, and data analysis by May 2025.

**Conclusions:**

INSIGHT is the first evaluation of a comprehensive survivorship program using real-world data; it will result in new information on the (cost-)effectiveness of survivorship care in survivors of HL in clinical practice. The results of this study will be used to improve the BETER program where necessary and contribute to more effective evidence-based long-term survivorship care for lymphoma survivors.

**International Registered Report Identifier (IRRID):**

PRR1-10.2196/55601

## Introduction

Cardiovascular diseases (CVDs) and second malignancies, especially breast cancer, are well-known late complications of Hodgkin lymphoma (HL) treatment. The risks of CVD and breast cancer are highest in survivors of HL treated with chest radiotherapy with or without anthracycline-based chemotherapy [1-8]. In addition, survivors of HL may also face other late treatment effects such as severe infections, associated with (functional) asplenia [3,9], and hypothyroidism after neck radiotherapy [10,11]. Although improved chemotherapy and radiotherapy regimens have increased HL cure rates to 80%-90% over the past decades, the aforementioned treatment-related complications have led to excess morbidity and mortality in survivors of HL [3,4,12-14].

CVD risk is 4-6 times higher in survivors of HL treated in 1965-1995 than in the general population, with up to 50% cumulative CVD incidence 40 years after HL treatment [7]. Female survivors of HL who received chest radiotherapy in the aforementioned period have a 5 times higher breast cancer risk than women in the general population, with a 19% cumulative incidence 30 years after treatment [2]. In addition, infectious disease mortality in survivors of HL treated in 1965-2000 increased 8-fold compared to the general population [3]. Moreover, 25 years after HL treatment, the cumulative incidence of (subclinical) hypothyroidism was 44% in survivors treated between 1961 and 1989 [9]. Individual late treatment–related complication risks depend on the characteristics of patients and HL treatment (radiotherapy location, dose and volume, chemotherapy type and dose, and whether or not splenectomy was performed) and are expected to be lower in more recent treatment years as a result of improved radiotherapy techniques [2,3,5,7,9].

In 2009, the Dutch “Better care after lymphoma, Evaluation of long-term Treatment Effects and screening Recommendations” (BETER) consortium, a collaboration between hemato-oncologists, radiation oncologists, nurse practitioners, epidemiologists, a general practitioner (GP), and patient representatives, was established. While HL generally occurs at a young age (with the highest incidence between 20 and 40 years) and survivors have a long life expectancy where they can develop late effects, structured survivorship care for adult survivors of HL was lacking. Structured survivorship care may lead to the early detection of (risk factors for) late adverse events, enabling timely intervention, possibly leading to a lower burden of disease due to late effects and better quality of life in survivors of HL [15,16]. Therefore, the BETER consortium developed a nationwide infrastructure of outpatient clinics where 5-year survivors of HL are screened for late adverse effects of lymphoma treatment according to nationally approved screening guidelines. Recommendations in the guidelines were based on the available scientific evidence in survivors of HL, and, if this evidence was absent, in other high-risk populations (eg, *BRCA* mutation carriers for breast cancer screening recommendations) or on expert consensus [17,18].

In 2013-2016, the first BETER clinics started inviting 5-year survivors of HL for risk-based screening for late treatment–related complications. Of note, setting up a BETER clinic requires a substantial effort, that is, identification and tracing of patients and setting up local infrastructure, including consulting specialists such as cardiologists [17]. Therefore, several Dutch lymphoma treatment centers were only able to start or will start inviting survivors of HL for survivorship care in 2019-2024.

Other cancer survivorship care programs, especially for survivors of childhood cancer (often also including survivors of childhood HL), have been established in several countries worldwide. However, there is a lack of knowledge about their effectiveness in clinical practice. Studies that evaluated the yield of late events in these programs, almost exclusively concerned with CVD and breast cancer screening results, lacked a comparison group or used hypothetical cohorts or simulation models to estimate the potential health benefits from structured survivorship care [19-25]. The effect of the implementation of cancer survivorship programs on survivors’ burden of disease, quality of life, and cost-effectiveness has never been assessed using real-world data. Therefore, in the Improving Nationwide Survivorship care Infrastructure and Guidelines after Hodgkin lymphoma Treatment (INSIGHT) study, we aim to assess whether BETER survivorship care leads to reduced burden of disease from late adverse events after HL treatment, lower health care costs, better quality of life, and reduced health-related productivity losses compared to the absence of structured survivorship care.

## Methods

### Design

**Figure 1:**
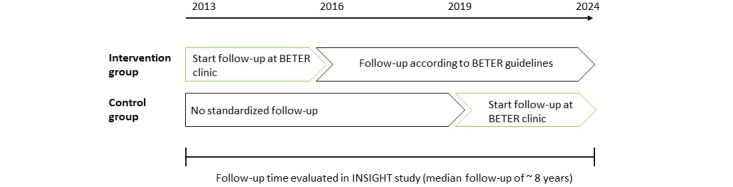
Schematic overview of the INSIGHT cohort study follow-up period among 5-year survivors of Hodgkin lymphoma in the Netherlands. BETER: Better care after lymphoma, Evaluation of long-term Treatment Effects and screening Recommendations.

The INSIGHT study is a retrospective cohort study with a quasi-experimental design and prospective follow-up, embedded in the national BETER survivorship care infrastructure. The first BETER clinics started in 2013-2016 and the number of centers participating in the BETER program increased over the last few years; several centers started or will start a BETER clinic in 2019-2024. This provides the unique opportunity to evaluate the effectiveness of BETER survivorship care by comparing survivors who did and did not receive BETER survivorship care over the past 6-10 years (expected median follow-up of ~8 years). The intervention group will consist of 450 survivors of HL who were invited to first visit a BETER clinic in 2013-2016. The control group will consist of a matched group of 450 survivors of HL who were eligible for BETER survivorship care as of 2013-2016 but were not invited because they were treated in a lymphoma treatment center not starting a BETER clinic until 2019-2024 (Figure 1).

### Study Population

**Figure 2:**
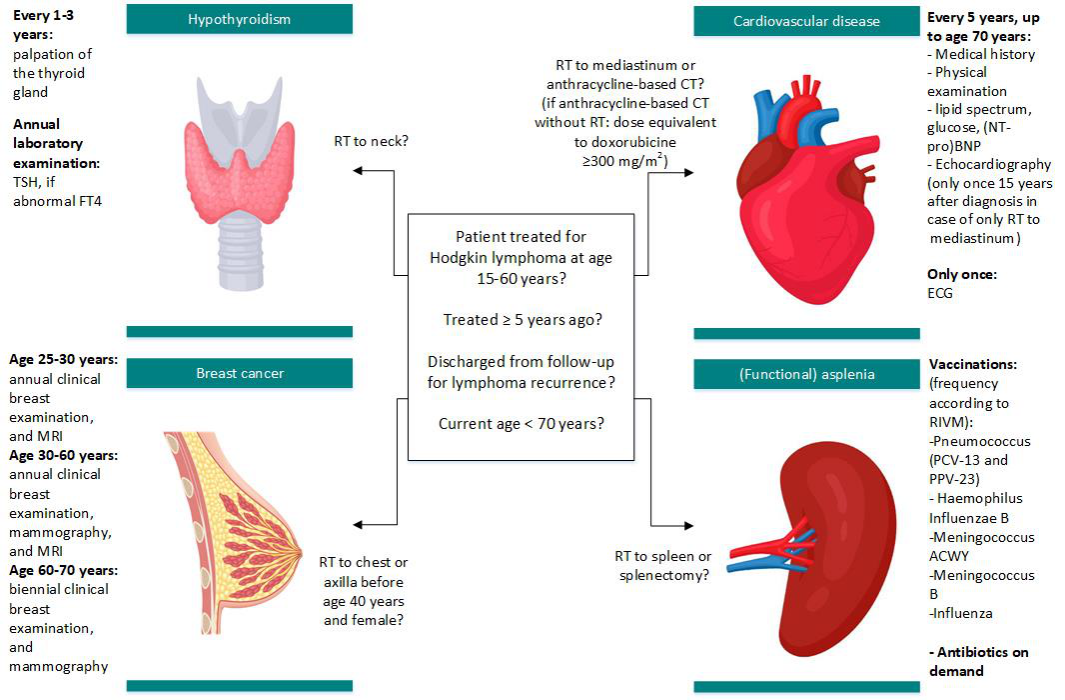
Simplified overview of BETER screening guidelines for 5-year survivors of Hodgkin lymphoma in the Netherlands [17, 18] and guideline asplenia from the National Institute for Public Health and the Environment (RIVM) [26]. CT: chemotherapy; ECG: electrocardiogram; FT4: free thyroxine; MRI: magnetic resonance imaging; (NTpro-)BNP: (N-terminal pro–)B-type natriuretic peptide; RIVM: National Institute for Public Health and the Environment, RT: radiotherapy; TSH: thyroid stimulating hormone.

According to the BETER guidelines, survivors of HL are eligible for survivorship care after completion of regular surveillance for HL recurrence (5 years after treatment), if they were 15-60 years at HL diagnosis, and are currently younger than 70 years of age. Figure 2 [17,18,26] shows a simplified overview of BETER eligibility criteria and the screening guidelines [17,18]. Survivors who do not have sufficient understanding of the Dutch language to complete the study survey, have developed an HL relapse during follow-up, whose vital status (and if alive current address) could not be verified, or who live abroad are excluded from the INSIGHT study.

The study participants in the control group are matched to patients in the intervention group based on the following survivor characteristics: sex, age at diagnosis (±5 years), and age in 2013 (±5 years) and the following treatment characteristics associated with increased risk of CVD, breast cancer, hypothyroidism, and (functional) asplenia: chest radiotherapy (yes or no), neck radiotherapy (yes or no), spleen radiotherapy or splenectomy (yes or no), and anthracycline-based chemotherapy (yes or no). Controls are selected from the comprehensive database of the previously established retrospective cohort of lymphoma survivors treated in participating BETER centers between 1965 and 2012. This database contains information on basic survivor characteristics and detailed treatment data [17].

### Outcomes

The primary outcomes of this study are the burden of disease (in disability-adjusted life years [DALYs]) from CVD including associated risk factors (Table S1 in Multimedia Appendix 1 [17,27-30]), breast cancer, severe infections (defined as fatal or requiring hospitalization) and hypothyroidism, the associated health care costs, and quality of life. In a cost-effectiveness analysis, the cost of BETER survivorship care will be weighed against averted or gained DALYs and quality-adjusted life years (QALYs). In order to assess indirect costs in both groups, health-related productivity losses and overall health care use will be calculated. Furthermore, the incidence rates (including stage and time since HL diagnosis) for the (risk factors for) late adverse events of interest (screen detected or nonscreen detected) will be compared between the intervention and control group.

The secondary outcomes of this study are BETER clinic nonattendance, guideline adherence, and knowledge and distress about late effects among survivors. Both the intervention and control group include survivors who attended the BETER clinic (attenders) and survivors who were invited for BETER survivorship care but chose to not attend (nonattenders). We will describe the reasons for nonattendance to BETER survivorship care and assess possible associations of nonattendance with socioeconomic status, health status, and other patient characteristics. This subanalysis will allow us to place our main study findings in perspective and possibly even improve future attendance rates. Survivors’ socioeconomic status will be derived from their zip code based on data from Statistics Netherlands [31]. We assess the BETER clinicians’ adherence to the guidelines by comparing the screening diagnostics that were performed in clinical practice to those that are recommended in the BETER guidelines. Finally, we will assess survivors’ knowledge about the risk of late complications (risk perception) in both groups and investigate whether this knowledge comes with increased or decreased distress about their risk of late complications.

### Follow-Up

For the intervention group, follow-up starts at the first BETER visit (or BETER clinic invitation date for nonattenders) and ends at the study inclusion of its matched control in 2019-2024 or at date of death, whichever comes first. For the control group, follow-up starts at the date their matched intervention group survivor started follow-up and ends at their first BETER visit (or BETER clinic invitation date for nonattenders), or, if earlier, at death. All study data will be stored for at least 20 years. This enables prospective follow-up of our cohort in order to study the long-term effects of screening on the incidence of late adverse events and the associated burden of disease.

### Recruitment and Informed Consent Procedure

For the detailed recruitment and informed consent procedure, refer to the detailed informed consent procedure section in Multimedia Appendix 1. In brief, in intervention centers, study patients are identified and linked to the national Personal Records Database to verify survivors’ vital status and current address. Subsequently, eligible survivors are invited for study participation by their (former) treating radiation oncologist or hematologist. In control centers, survivors who match an intervention group survivor are selected from the previously established cohort of survivors of HL from that specific center (Design section) [17]. Matched controls who are alive and do not experience adverse health outcomes in an advanced stage (eg, metastatic breast cancer or end-stage heart failure) are invited to the BETER clinic. After the invitation for BETER survivorship care, these controls are invited for study participation using the same procedures as for the intervention group. Survivors who do not respond to the study invitation will receive reminders; nonattenders receive 1 reminder approximately 4 weeks after the initial invitation and attenders receive 2 reminders after approximately 4 and 8 weeks, respectively. Survivors who died during follow-up are included without informed consent.

#### Data Collection

**Figure 3:**
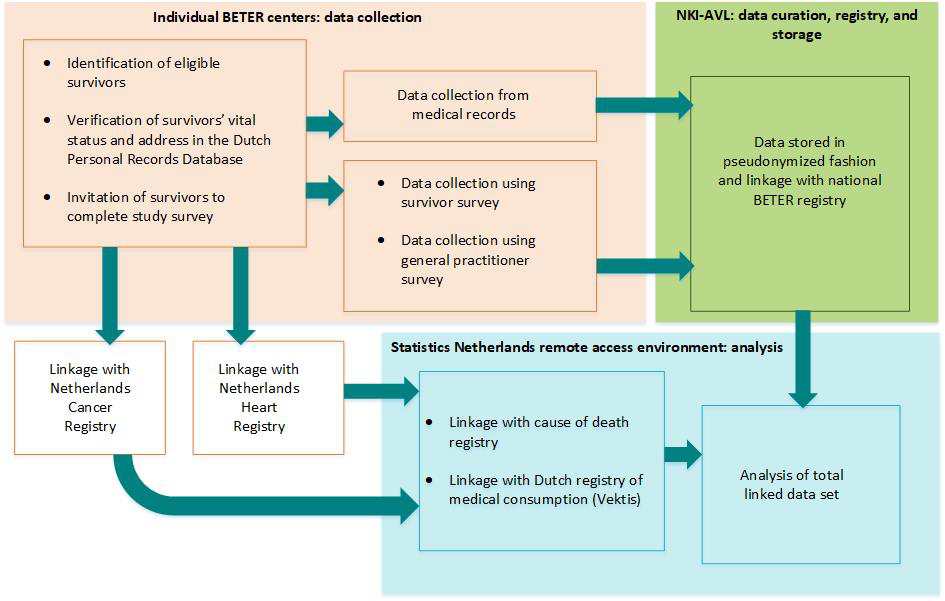
Data collection procedures for the INSIGHT cohort study at individual BETER survivorship care clinics for 5-year survivors of Hodgkin lymphoma in the Netherlands and planned linkages with national disease registries. BETER: Better care after lymphoma, Evaluation of long-term Treatment Effects and screening Recommendations; NKI-AVL: The Netherlands Cancer Institute.

Basic survivor characteristics and detailed treatment information for the included survivors are extracted from the comprehensive database of lymphoma survivors treated in participating BETER centers between 1965 and 2012 [17]. For the INSIGHT study, we collect additional data from multiple sources: medical records, a survivor survey, a GP survey, and national registries. We provide explanations on each of the data collection procedures in the upcoming sections Medical Records, Survivor Survey, GP Survey, and Linkage With National Registries and in Figure 3.

##### Medical Records

For attenders of the BETER clinics in the intervention group, complete follow-up data from the BETER clinic (ie, performed screening tests, results, diagnoses, and subsequent treatments) are collected from the medical records. For attenders in the control group, the date of the first BETER visit and the date of the last regular radiation oncology or hemato-oncology outpatient clinic visit are collected.

##### Survivor Survey

For attenders of the BETER clinics, the survivor survey includes, among others, the following standardized questionnaires: the EQ-5D-5L questionnaire yields utilities used to calculate QALYs, the 36-Item Short Form Health Survey is used to cross-sectionally assess the quality of life, and the Institute for Medical Technology Assessment Productivity Cost questionnaire is used to assess health-related productivity losses [32-34]. Textbox 1 shows an overview of the total study survey components for attenders of BETER care. The expected survey completion time for attenders of BETER care is 20-55 minutes depending on medical history. Nonattenders of the BETER clinics are asked to complete a survey that contains the following components: (1) a questionnaire assessing reasons to not attend a BETER clinic and possible screening or treatment for late effects elsewhere, (2) a short questionnaire on their current health status and lifestyle, and (3) the 6-item adapted version of the Cancer Worry Scale to determine distress associated with the increased risk of late effects [35]. The expected survey completion time for nonattenders is 10-25 minutes. Attenders in the intervention and control group receive the same survey; the same applies to the nonattenders in the intervention and control group.

**Overview of Improving Nationwide Survivorship care Infrastructure and Guidelines after Hodgkin lymphoma Treatment cohort study survey components for 5-year survivors of Hodgkin lymphoma who attended survivorship care at different Better care after lymphoma, Evaluation of long-term Treatment Effects and screening Recommendations (BETER) centers in the Netherlands. The survey is completed by study participants at enrollment in the period 2021-2024.**

- EQ-5D-5L questionnaire (a standardized questionnaire that yields utilities used for calculating quality-adjusted life years)
- 36-Item Short Form Health Survey (standardized quality of life questionnaire)
- Institute for Medical Technology Assessment Productivity Cost Questionnaire (standardized health-related productivity losses questionnaire)
- Medical consumption (including general practitioner care, hospital care, physiotherapist, psychologist, and psychiatrist consultations)
- Knowledge about late adverse effects
- Satisfaction with BETER care and perception of benefit and burdensomeness of BETER care
- 6-item adapted Dutch version of the Cancer Worry Scale to determine distress about late adverse effects
- Current health status (diagnoses of possible late effects, medication use, and lifestyle). These questions are only included when the last BETER clinic visit was > 6 months ago

##### GP Survey

Each survivor’s GP is approached by the survivor’s former treating radiation oncologist or hematologist with a request to provide information for the INSIGHT study. Each GP receives a short paper survey enquiring about the survivor’s medical history of (risk factors for) CVD, severe infections, vaccination status, hypothyroidism, and, if applicable, cause of death. The GP receives 2 reminders in case of nonresponse.

##### Linkage With National Registries

For all survivors included in the study (intervention and control group), we will obtain information on the incidence of clinical events of interest through linkage with disease registries. Information on breast cancer diagnoses will be obtained from Netherlands Cancer Registry, information on cardiac interventions and disease episodes will be obtained from the Netherlands Heart Registry, information on hospital admissions for CVD and serious infections and total medical consumption will be obtained from the Dutch health care costs registration (Vektis), and for those who died during follow-up cause of death will be obtained from Statistics Netherlands [36-39]. Furthermore, linkage with the national BETER registry data, containing BETER questionnaire data, will be performed. The BETER questionnaire is completed by survivors before their first BETER visit and contains extensive questions about current health, medical history, and lifestyle, which helps health care providers focus on actual symptoms of late adverse effects and lifestyle during the consultation [17]. The questionnaire answers are registered in the national BETER registry for research into late effects of lymphoma treatment upon survivor’s written informed consent [17]. Due to the privacy regulations of Statistics Netherlands, the final linked data set can only be accessed and analyzed in the protected remote environment of Statistics Netherlands.

### Ethical Considerations

The study protocol was approved by the institutional review board of the Netherlands Cancer Institute (IRB21-115), and we complied with local ethical approval guidelines at all participating sites. Survivors who opted out of the use of their medical data for the INSIGHT study, or medical research in general, are excluded from the study (refer to the detailed informed consent procedure section in Multimedia Appendix 1). Participating survivors did not receive financial compensation. Patient-identifying information is pseudonymized at the participating sites before storage at the Netherlands Cancer Institute and before data analysis in the Statistics Netherlands remote access environment.

#### Statistical Analysis

##### Burden of Disease and Secondary Outcomes

DALYs for the conditions of interest are calculated as the sum of the “years of life lost” (YLL) due to premature mortality and the “years lost due to disability” (YLD) for people living with the condition: DALY=YLL+YLD. The YLL is calculated by the number of deaths multiplied by the standard life expectancy at the age at which death occurs. Standard life expectancy in the Netherlands is available from Statistic Netherlands [40]. To estimate YLD during the follow-up period, the number of cases in that period will be multiplied by the average duration of the disease and a weight factor (disability weight) that reflects the severity of the disease on a scale from 0 (perfect health) to 1 (dead). Disability weights are extracted from the Global Burden of Disease Study 2019 or the most recent Global or preferably Dutch disability weight studies [41].

For continuous outcomes (eg, DALYs and health-related quality of life), multiple linear regression analysis will be used to study differences between the intervention and the control group. Important confounders will be controlled for by matching survivors in the intervention group to survivors in the control group based on patient and treatment characteristics, see Study population section. The influence of other potential confounders (eg, BETER hospital or socioeconomic status) on the regression coefficients will be examined, and these will be included in the model when associated with a ≥10% change in the regression coefficient.

For count data (eg, the incidence of late adverse events), negative binomial regression analysis or Poisson regression analysis (depending on the distribution of the data) will be used. To analyze the time between HL diagnosis and diagnosis of late adverse events, competing risk regression methods will be used, with death treated as a competing risk.

Missing data will be handled by multiple imputation when data are missing (completely) at random. Data analysis will be performed in R and RStudio software (The R Foundation).

##### Sample Size Calculation

The complete sample size calculation including detailed substantiation can be found in the Detailed sample size calculation section in Multimedia Appendix 1. In brief, in an intention-to-treat analysis, taking a BETER clinic attendance rate of 65% into account (A Nijdam, unpublished data, 2016), the expected difference between the intervention and the control group is 0.40 acquired DALY over a follow-up period of 5 years (our expected median follow-up is longer). With a 2-sided significance level of .05 and 80% power to detect a difference of 0.20 SD (small effect size) and larger, we need 393 patients in each study arm. When we take a 15% loss to follow-up and incomplete data into account, we need 452 patients in each study arm.

##### Cost-Effectiveness Analysis

To compare direct and indirect health care costs between the intervention and the control group, the Dutch health care costs registration (Vektis) and the handbook of costing studies from the Dutch National Health Care Institute (ZorgInstituut) will be used [39,42]. The effects of BETER survivorship care in comparison to the absence of standardized follow-up will be expressed in costs/DALY and costs/QALY. QALYs are derived by health state utilities. A utility is a standardized score between 0 and 1, with 0 reflecting death and 1 perfect health, measured by the EQ-5D-5L questionnaire [33]. The cost-effectiveness analysis will be performed using a discrete event simulation, where we will incorporate several screening strategies. A discrete event simulation analysis on the patient level will be used to simulate the sequences of events that occur as a continuous process over time and to calculate the mean associated costs and QALYs [43]. Uncertainty around the results will be quantified using nonparametric bootstrapping and cost-effectiveness acceptability curves. These curves will show whether BETER survivorship care is cost-effective or not for various values of the Dutch society’s willingness to pay for 1 QALY. A budget impact analysis will be performed to estimate the 5-year financial consequences of the implementation of BETER survivorship care in the Dutch health care system. The following factors will be accounted for in the budget impact analysis: screening costs, the potential number of survivors of HL eligible for screening, and the costs of treatment of late adverse events.

## Results

The study was funded in November 2020 and approved by the institutional review board of the Netherlands Cancer Institute in July 2021. The first survivor was included in November 2021, and by November 2023, a total of 445 survivors were included in the study intervention group, and 34 survivors were included in the control group. We expect to finalize recruitment by October 2024, data collection by early 2025, and data analysis by May 2025.

## Discussion

### Principal Findings

BETER is an internationally unique comprehensive infrastructure for adult oncology survivorship care. The INSIGHT study evaluates the effect of the implementation of BETER survivorship care for survivors of HL on survivors’ burden of disease from late adverse events, associated health care costs, and quality of life. It will result in important new information on the (cost-)effectiveness of survivorship care in survivors of HL in clinical practice. With our study results, we will be able to evaluate current BETER screening guidelines and adapt them accordingly, if indicated [17,18]. These study results can therefore be used to improve the BETER survivorship care infrastructure and contribute to more effective evidence-based long-term survivorship care for survivors of HL. Moreover, some results could possibly be extrapolated to other cancer survivors (eg, survivors of breast cancer treated with anthracyclines). Importantly, the knowledge gained in this study can be used to better inform survivors of HL about the possible benefits of screening.

If our study demonstrates the cost-effectiveness of BETER survivorship care, we expect that more hospitals in the Netherlands will be motivated to reallocate resources to overcome the organizational hurdles to implement survivorship care according to the BETER guidelines, thus improving implementation of BETER care.

Taking into account the increasing number of cancer diagnoses, improving survival rates, and increasing health care costs, it is essential to ensure that BETER survivorship care is feasible and cost-effective in the long term. If we are able to identify diagnostic tests that have little added value in terms of diagnostic yield and clinical benefit, we will look into leaving them out in future BETER guidelines. Our results may demonstrate that additional diagnostics not recommended in the guidelines, which do not contribute to the cost-effectiveness of BETER survivorship care, are routinely performed. Also, we may observe that survivors are recalled for surveillance more frequently than advised in the guidelines. We can then provide this feedback to the centers in question, as it is not only important to reduce costs but also to make sure we do not overmedicalize survivors of HL.

### Strengths and Limitations

An important strength of this study is the quasi-experimental design, which takes advantage of the situation in which BETER care has been gradually implemented in different lymphoma treatment centers in the Netherlands over the past decade. This provides the unique opportunity to study the added value of BETER survivorship care by comparing survivors who did receive such care over the past 6-10 years with a matched comparison group that did not, thereby minimizing the risk of bias in our observational study. A second strength of this study is the comprehensive data collection on the incidence rates of late adverse events from multiple sources: survivors, survivors’ medical records, survivors’ GPs (and if necessary medical specialists), and national disease registries. This allows us to verify our data, in order to overcome uncertainties and selection bias related to patient-reported outcomes and potential missing data, for example, due to failed linkages. Third, very importantly, this study design provided us with a representative study population. As we have identified the entire cohort of 5-year survivors who were treated for HL in the centers participating in BETER, we can also include survivors who died during follow-up [17]. Moreover, we also include the data of survivors who did not respond to the invitation to visit the BETER clinic and those who did not respond to our INSIGHT study invitations. The only survivors not included in our study are those who explicitly objected to the use of their data for the INSIGHT study or medical research in general (expected 2%-7%). Including nonattenders of BETER care in this study enables us to accurately assess the total number of survivors eligible for BETER care and perform the cost-effectiveness analysis from a societal perspective of the Netherlands.

The first limitation of our analysis of patient-reported outcomes such as quality of life and risk perceptions is that our results can be affected by selection bias, as we expect 60%-70% of survivors to complete our study survey. A second limitation may be that our sample size calculation is based on a cumulative difference in the burden of disease of CVD, breast cancer, severe infections, and hypothyroidism. Therefore, we may have limited power to perform subanalyses of parts of the disease-specific screening guidelines separately. A third limitation is that we could only follow survivors treated for HL from 1971 to 2011 who were screened for late adverse events from 2013 onward. As late adverse events of interest may have already developed before the start of the BETER screening program in a substantial proportion of the included survivors, we may underestimate the yield and potential benefit of screening. Finally, the expected study follow-up of approximately 8 years may not be sufficient to accumulate enough events of interest to be able to detect a significant difference in the burden of disease between the groups. Therefore, we plan to prospectively follow our cohort and repeat linkages with disease registries after 5-10 years.

### Comparison With Prior Work

The INSIGHT study is not only the first evaluation of survivorship care provided at BETER clinics but also the first cost-effectiveness study of structured cancer survivorship care worldwide that uses real-world data and focuses on multiple types of screening. Previous studies performed on survivors of HL and childhood cancer reported on the screening yield of 1 type of screening, lacked a valid comparison group, or used simulations models or hypothetical cohorts to study its cost-effectiveness [19-25,44].

For example, the study by Chow et al [23] reported on underdiagnosis and undertreatment of hypertension, hypercholesterolemia, and diabetes in adult survivors of childhood cancer at high risk of premature CVD in the United States Childhood Cancer Survivor Study cohort and compared the prevalence rates to the general population [23]. Furthermore, in the St. Jude Lifetime Cohort study of childhood cancer survivors, Armstrong et al [24] and Palmer et al [25] evaluated echocardiographic detection rates of impaired left ventricular ejection fraction and diastolic dysfunction, respectively [24,25]. Howell et al [44] described breast cancer cases detected in the United Kingdom national GP mammography screening program for survivors of HL and compared the findings (eg, stage) to breast cancer cases diagnosed in the general population. Although these studies provide valuable information, the results do not demonstrate the incremental value of screening due to the lack of a valid comparison group.

Chen et al [21] evaluated lipid screening and statin therapy in a hypothetical cohort of 30-year-old 5-year survivors of HL treated with chest irradiation. The authors compared no screening with screening at 1-, 3-, 5-, or 7-year intervals and used Markov models to calculate life expectancy, quality-adjusted life expectancy, and lifetime cost. Wong et al [20] simulated life histories using Markov health states to assess the effect of the implementation of echocardiographic screening followed by angiotensin-converting enzyme inhibitor and β-blocker therapy in survivors of childhood cancer on cost-effectiveness, life expectancy, QALYs, and the cumulative incidence of heart failure 30 years after a cancer diagnosis. An important limitation of the above-mentioned studies is that the actual efficacy of statins, angiotensin-converting enzyme inhibitors, and β-blockers in survivors of HL and childhood cancer is unknown and therefore had to be estimated based on assumptions. Furzer et al [22] used simulation models to evaluate the cost utility of 8 different breast cancer screening strategies for survivors of HL treated with chest radiotherapy, with annual magnetic resonance imaging and mammography from age 25 years onward as a reference (no comparison to population screening only). A limitation of this study is that model assumptions about the efficacy of breast cancer screening at young ages were mostly based on *BRCA1*/*2* mutation carriers, who are known to have different breast cancer tumor characteristics than other women [45].

The German Hodgkin lymphoma study group described the yield of hypothyroidism with thyroid-stimulating hormone screening in survivors of HL (median time since treatment 6 years, median follow-up of 70 months, IQR 12-243 months) [11]. Our study will examine the efficacy of thyroid-stimulating hormone screening after longer time intervals since HL treatment and will also provide longer follow-up after the start of screening. To our knowledge, no studies describing health gains from vaccinations or prescription of on-demand antibiotics in (functional) asplenic survivors of HL have been published. The current BETER afunctional spleen guideline is based on knowledge about patients who had a splenectomy for other indications and on the known increased mortality from infectious disease after spleen irradiation in survivors of HL [3,9,17,18,26]. This study will provide the opportunity to compare incidences of severe infections among survivors of HL who did and did not receive the recommended vaccinations and antibiotics.

Little is known about the effect of survivorship care on survivors’ knowledge about late adverse effects and possible distress about these risks. Signorelli et al [46] performed a systematic review and described that attenders of survivorship care (compared to nonattenders) demonstrated increased knowledge about their treatment and diagnosis, and although not significantly, attenders tended to report more distress associated with the risk of late effects [46]. With our study, we can identify areas of insufficient risk perception in survivors of HL attending the Dutch survivorship program, with the aim of improving risk communication by BETER health care providers.

### Conclusions

INSIGHT is the first evaluation of (cost-)effectiveness of structured cancer survivorship care for survivors of HL based on real-world data. It will result in new knowledge on the (cost-)effectiveness of survivorship care in lymphoma survivors in clinical practice. The results are expected to contribute to more effective evidence-based long-term lymphoma survivorship care and could also be of interest to other survivorship programs.

## Appendix

Multimedia Appendix 1 Detailed informed consent and recruitment procedure, detailed sample size calculation, and overview of cardiovascular diseases and associated risk factors included in the INSIGHT study.

Multimedia Appendix 2 Project peer-review by funder.

## Abbreviations

BETER: Better care after lymphoma, Evaluation of long-term Treatment Effects and screening Recommendations
CVD: cardiovascular disease
DALY: disability-adjusted life year
GP: general practitioner
HL: Hodgkin lymphoma
INSIGHT: Improving Nationwide Survivorship care Infrastructure and Guidelines after Hodgkin lymphoma Treatment
QALY: quality-adjusted life year
YLD: years lost due to disability
YLL: years of life lost

## Data Availability

The data sets generated and analyzed during this study are available from the corresponding author on reasonable request.
