# Supplementary material for: Effectiveness and Cost-Effectiveness of Survivorship Care for Survivors of Hodgkin Lymphoma (INSIGHT Study): Protocol for a Multicenter Retrospective Cohort Study With a Quasi-Experimental Design
Source: JMIR Res Protoc. 2024 Apr 18;13:e55601. doi: 10.2196/55601 (PMC11066749; doi:10.2196/55601)
Supplement: Multimedia Appendix 1 [file resprot_v13i1e55601_app1.docx]

## A.1 Detailed recruitment and informed consent procedure

In intervention centers, study patients are identified and linked to the national Personal Records Database to verify survivors’ vital status and current address. Subsequently, eligible survivors who are alive receive a study invitation letter from their (former) treating radiation oncologist or hematologist. In the letter, survivors also receive a link to access a short informative animation video explaining the INSIGHT study rationale. Participation involves completion of a survey and an informed consent form. Permission is requested to combine the survey data with medical data available in their lymphoma treatment center, additional medical data from their general practitioner (GP), medical specialists and national disease registries. The survey and informed consent form can be filled out online and can be accessed by a personal link to Castor Electronic Data Capturing system, but a paper version can be sent to survivors upon request. In control centers, survivors who match an intervention group survivor are selected from the previously established cohort of HL survivors from that specific center (see section Design) [17]. Matched controls who are alive and do not suffer from adverse health outcomes in an advanced stage (e.g. metastatic breast cancer or end-stage heart failure), are invited to the BETER clinic. After invitation for BETER survivorship care, the same recruitment and informed consent procedures as in intervention group survivors are carried out for matched controls.

Survivors who do not respond to the study invitation, will receive reminders; non-attenders receive one reminder approximately four weeks after initial invitation and attenders receive two reminders after approximately four and eight weeks, respectively. A paper version of the study survey and informed consent form is included with the last reminder letter.

Survivors who object to the use of their medical data for the INSIGHT study, are asked to opt out for study participation within four weeks after the last reminder, by either e-mail or telephone. In accordance with Dutch law (WGBO, article 7:458), data from survivors who do not respond to any of the study invitation letters may be used, as they had the explicit opportunity (at least twice) to refuse participation, and not including them could result in a significant selection bias, making it impossible to evaluate the (cost-)effectiveness of BETER survivorship care. Also in accordance with Dutch law (WGBO, article 7:458), survivors in the control group who are already known with the disease(s) of interest in an advanced stage (see above) and are therefore not invited to the BETER clinic, are included but exempt from the study informed consent procedures. Asking these survivors for informed consent may cause considerable psychological stress in the last phase of their lives. Obviously, survivors who opted out for the use of their medical data for the INSIGHT study, or for medical research in general, are not included in the study. According to Dutch law, deceased survivors are included without informed consent. The study protocol was approved by the Institutional Review Board of the Antoni van Leeuwenhoek (IRB21-115) and we complied with local ethical approval guidelines at all participating sites.

## A.2 Detailed sample size calculation

To assess the potential health gains (in terms of averted DALYs) of BETER survivorship care, we constructed a model in which the burden of disease accumulated during 5-year follow-up (of note: the median follow-up in the study is expected to be eight years) was calculated for the intervention group and the control group. The model includes cumulative incidence of the late adverse events of interest: (risk factors of) CVD, breast cancer, severe infections due to (functional) asplenia and hypothyroidism. The impact of treating the CVD risk factors hypertension and hypercholesterolemia on developing CVD and death from CVD was inferred from risk reduction tables published by the Dutch Society of General Practitioners in 2016 [27]. Disability weights for CVD were extracted from the Dutch and Global Burden of Disease studies [28, 29]. The number of DALYs averted by breast cancer screening was based on a cost-effectiveness study on breast cancer interventions [30]. Based on this model, we estimated that the average HL survivor of 52 years of age, who is not screened for late adverse treatment events, acquires 1.18 DALYs in the next five years (1.12 for breast cancer + CVD; 0.04 for (functional) asplenia; 0.01 for hypothyroidism), as opposed to 0.56 DALYs for a survivor screened at a BETER clinic (0.55 for breast cancer + CVD; 0.00 for functional asplenia; 0.00 for hypothyroidism) (numbers do not add up due to rounding). We therefore assumed that, at patient level, screening at the BETER clinics averts on average 0.62 DALYs per survivor in a 5-year period. Attendance to BETER clinics that started in 2013-2016 was 60-70% (*unpublished data by A. Nijdam, 2016)*. In an intention-to-treat analysis, taking an attendance rate of 65% into account, the expected difference between the intervention and the control group is 0.40 DALY: 0.78 DALYs in the intervention group (65% of 0.56 DALYs + 35% of 1.18 DALYs) and 1.18 DALYs in the control group. With a 2-sided significance level of 0.05 and 80% power to detect a difference of 0.20 standard deviation (small effect size) and larger, we need 393 patients in each study arm. When we take 15% loss to follow-up and incomplete data into account, we need 452 patients in each study arm.

**Table S1.** Overview of the (risk factors for) CVD studied in the INSIGHT cohort study among 5-year Hodgkin lymphoma survivors in the Netherlands.

| **Cardiovascular risk factors** |
| --- |
| Hypertension |
| Hypercholesterolemia |
| Hypertriglyceridemia |
| Diabetes Mellitus |
| Smoking |
| **CVDs** |
| Coronary artery disease |
| Clinically relevant valve dysfunction (i.e. aortic, pulmonary, tricuspid, mitral valve regurgitation or stenosis) |
| Dysrhythmia or conduction abnormality |
| Heart failure |
| Cardiomyopathy |
| *Abbreviation: CVD(s) = cardiovascular disease(s)* |
