# Supplementary material for: Effectiveness and Cost-Effectiveness of Survivorship Care for Survivors of Hodgkin Lymphoma (INSIGHT Study): Protocol for a Multicenter Retrospective Cohort Study With a Quasi-Experimental Design
Source: JMIR Res Protoc. 2024 Apr 18;13:e55601. doi: 10.2196/55601 (PMC11066749; doi:10.2196/55601)
Supplement: Multimedia Appendix 2 [file resprot_v13i1e55601_app2.pdf]

Algemene gegevens / General Information

Programma / Programme	:	**Evaluatieonderzoek ZE&GG**
Subsidieronde / Subsidy round	:	**Evaluatieonderzoek ronde 2019 - UA**
Projecttitel / Project title	:	**Evaluation of nationwide long-term follow-up care for lymphoma survivors in the Netherlands: does survivorship care at the BETER clinics reduce morbidity and mortality from late effects of lymphoma treatment and associated costs?**
Projecttaal / Project language	:	**Engels / English**
Geplande startdatum / Planned start date	:	**02-11-2020**
Geplande duur / Planned duration	:	**54 maanden / months**
Datum indienen / Date of application	:	**27-08-2020**
Projecttype / Project type	:	**Toegepast onderzoek / Applied research**
Vervolg eerder ZonMw-project / Continuation previously funded project ZonMw	:	**Nee / No**

Projectleden / Project members

Prof. dr. F.E. Van Leeuwen (Main applicant)
Functie / Position: Head Dept. Psychosocial Research and Epidemiology | *Opleiding / Education:*
Studierichting / Subject:
T: 020-512 2483 | *F:* | *E:* f.v.leeuwen@nki.nl

Nederlands Kanker Instituut
Psychosociaal Onderzoek en Epidemiologie
Epidemiology
Postbus 90203
1006 BE AMSTERDAM

Prof. dr. F.E. Van Leeuwen (Projectleader and secretary)
Functie / Position: Head Dept. Psychosocial Research and Epidemiology | *Opleiding / Education:*
Studierichting / Subject:
T: 020-512 2483 | *F:* | *E:* f.v.leeuwen@nki.nl

Nederlands Kanker Instituut
Psychosociaal Onderzoek en Epidemiologie
Epidemiology
Postbus 90203
1006 BE AMSTERDAM

Prof. dr. E. Voest MD (Administrative responsibility)
Functie / Position: Raad van Bestuur en Medisch directeur NKI/AVL | *Opleiding / Education:*
Studierichting / Subject:
T: 020-512 6273 | *F:* | *E:* e.voest@nki.nl

Nederlands Kanker Instituut
Raad van Bestuur
Postbus 90203
1006 BE AMSTERDAM

Dr. B.M.P. Aleman MD (Co-projectleader)
Functie / Position: Radiotherapeut oncoloog | *Opleiding / Education:*
Studierichting / Subject:
T: 020-512 2141 | *F:* | *E:* b.aleman@nki.nl

Nederlands Kanker Instituut
Radiotherapie
Postbus 90203
1006 BE AMSTERDAM

C.E.I.M. van Dierendonck (Project advisor)
Functie / Position: Patiëntvertegenwoordiger | *Opleiding / Education:*
Studierichting / Subject:
T: 0402410907 | *F:* | *E:* cecile.van.dierendonck@hematon.nl

Stichting Hematon
Leukemie en MDS

Postbus 8152
3503 RD UTRECHT

Prof. dr. A.H.E.M. Maas MD (Project advisor)
Functie / Position: Hoogleraar Cardiologie voor vrouwen | *Opleiding / Education:*
Studierichting / Subject:
T: 024-361420 | *F:* | *E:* angela.maas@radboudumc.nl

Radboudumc
Cardiologie

Postbus 9101
6500 HB NIJMEGEN

M.Sc. A. Nijdam PhD (Co-Applicant)
Functie / Position: Coordinator BETER-project | *Opleiding / Education:*
Studierichting / Subject:
T: 020-512 6132 | *F:* | *E:* a.nijdam@nki.nl

Nederlands Kanker Instituut
Psychosociaal Onderzoek en Epidemiologie
Epidemiology

Postbus 90203
1006 BE AMSTERDAM

Dr. V.P. Retèl (Co-Applicant)
Functie / Position: Head Health Technology Assessment Facility | *Opleiding / Education:*
Studierichting / Subject:
T: 020-512 6197 | *F:* | *E:* v.retel@nki.nl

Nederlands Kanker Instituut
Psychosociaal Onderzoek en Epidemiologie
Epidemiology

Postbus 90203
1006 BE AMSTERDAM

Prof. dr. ir. W.M.M. Verschuren (Project advisor)
Functie / Position: Hoogleraar Healthy vascular ageing public health | *Opleiding / Education:*
Studierichting / Subject:
T: 030-2749111 | *F:* | *E:* monique.verschuren@rivm.nl

Rijksinstituut voor Volksgezondheid en Milieu
Centrum voor Voeding, Preventie en Zorg
Determinanten Chronische Ziekten

Postbus 1
3720 BA BILTHOVEN

Prof. dr. J.M. Zijlstra MD (Co-projectleader)
Functie / Position: Hematoloog-internist | *Opleiding / Education:*
Studierichting / Subject:
T: +31 20 444 2604 | *F:* | *E:* j.zijlstra@amsterdamumc.nl

Amsterdam UMC - locatie VUmc
Hematologie
CCA Room 4.24

Postbus 19268
1000 GG AMSTERDAM

Projectgegevens / Project information

Aandachtsgebieden / Focus

Fase in zorgproces
- Nazorg

- Screening

Populatie
- Volwassenen

Setting
- Ziekenhuiszorg

Wetenschappelijke en/of beroepsverenigingen
- Interne Geneeskunde (NIV)
- Radiotherapie en Oncologie (NVRO)

Samenvatting / Summary

DOEL
In de klinische praktijk de (kosten-)effectiviteit bepalen van gestructureerde nazorg voor lymfklierkanker overlevenden.

HYPOTHESE
Screening op en vroege behandeling van late neveneffecten van lymfoombehandeling leiden tot een lagere ziektelast van late effecten, lagere zorgkosten en een betere levenskwaliteit in vergelijking met het ontbreken van gestructureerde nazorg.

PATIËNT (P)
Interventiegroep: eerste 450 Hodgkinlymfoom(HL)-overlevenden uitgenodigd voor BETER-nazorg in 2013-2016.
Controlegroep: 450 HL-overlevenden in aanmerking komend voor BETER-nazorg in 2013-2016, maar niet uitgenodigd omdat hun behandelcentrum pas in 2021-2023 een BETER-poli start.

INTERVENTIE (I)
Gepersonaliseerde screening en behandeling van (risicofactoren voor) hart- en vaatziekten (HVZ), borstkanker, hypothyreoïdie en asplenie op een BETER-poli.

COMPERATOR (C)
Ontbreken van gestructureerde nazorg na ontslag van controle voor HL (5 jaar na behandeling).

OUTCOME (O)
Primair: ziektelast (in ‘disability-adjusted life-years’, DALYs) als gevolg van HVZ, borstkanker, hypothyreoïdie en asplenie, bijbehorende gezondheidszorgkosten, kwaliteit van leven.
Secundair: opkomst van BETER-poli’s, naleving van richtlijnen, kennis van late effecten.

FOLLOW-UP
Mediaan 8 jaar: van ~2014 tot ~2022.

STUDIE OPZET
Retrospectieve cohortstudie met quasi-experimentele opzet.

SAMPLE SIZE BEREKENING/DATA-ANALYSE
Regressieanalyses worden uitgevoerd op ‘intention to screen’ en patiëntniveau. Met verwachte 5-jaar cumulatieve DALY’s van 0,78 in de interventie- en 1,18 in de controlegroep, hebben we in de ‘intention to screen’ analyse, met a = 0,05 en 80% power, 452 patiënten in elke studiearm nodig om een verschil van minimaal 0,20 standaarddeviatie te detecteren. Hiermee kunnen we ook medium effectgroottes aantonen in sub-analyses.

KOSTENEFFECTIVITEITSANALYSE/ BUDGET IMPACT ANALYSE
Kosteneffectiviteitsanalyse: vergelijking van de kosteneffectiviteit van BETER-nazorg met het ontbreken van deze zorg.
Perspectief: vanuit Nederlandse gezondheidszorg en maatschappij.
Tijdshorizon: 5 jaar (o.b.v. dit onderzoek) en levenslang (o.b.v. extrapolaties in een patiënt-level model).
Effectmaat: kosten/DALY en kosten/QALY.
Budget impact analyse: schatting van financiële gevolgen van BETER-nazorg over een 5-jaarsperiode in de Nederlandse gezondheidszorg.

TIJDPAD
Totaal: 4,5 jaar.
Voorbereiding + ethische goedkeuring: 0,75 jaar.
Patiëntinclusie + dataverzameling: 2,75 jaar.
Analyse + rapportage: 1,0 jaar.

OBJECTIVE(S)
To assess (cost-)effectiveness of structured survivorship care for lymphoma survivors in clinical practice and to identify possible areas of improvement.

HYPOTHESIS
Screening for and early treatment of late adverse effects of lymphoma treatment leads to reduced burden of disease from late effects, lower healthcare costs and better quality of life compared to the absence of structured survivorship care.

PATIENT (P)
Intervention group: first 450 Hodgkin lymphoma (HL) survivors invited for BETER survivorship care in 2013-2016.
Control group: 450 HL survivors eligible for BETER survivorship care in 2013-2016, but not invited as their treatment centre starts a BETER clinic in 2021-2023.

INTERVENTION (I)
Risk-based screening for and treatment of (risk factors for) cardiovascular disease (CVD), breast cancer, hypothyroidism and functional asplenia in a BETER clinic.

COMPRATOR (C)
Absence of structured survivorship care after discontinuation of follow-up for lymphoma recurrence (usually 5 years after treatment).

OUTCOME (O)
Primary: burden of disease (in disability-adjusted life-years, DALYs) from CVD, breast cancer, hypothyroidism and functional asplenia, associated health care costs, quality of life and health-related productivity losses.
Secondary: BETER clinic attendance, guideline adherence and knowledge and risk perception of late effects.

FOLLOW-UP TIME
Median 8 years, from ~2014 to ~2022.

STUDY DESIGN
A retrospective cohort study with a quasi-experimental design.

SAMPLE SIZE CALCULATION/DATA ANALYSIS
Regression analyses will be performed at intention to screen and patient level. At intention to screen level, expecting 5-year accumulation of 0.78 DALYs in the intervention vs.1.18 in the control group, with a=0.05 and 80% power to detect a difference of 0.20 standard deviation and larger, we need 452 patients in each study arm. This also allows for detection of medium effect sizes in sub analyses.

COST-EFFECTIVENESS ANALYSIS/ BUDGET IMPACT ANALYSIS
Cost-effectiveness analysis will compare costs and effects of BETER survivorship care vs. usual care, from a Dutch healthcare and societal perspective, using a 5-years (study-based) and long-term (based on extrapolations in a patient-level model) time horizon. Effects will be expressed in costs/DALY and costs/QALY. Budget impact analysis will estimate 5-year financial consequences of adoption of BETER care.

TIME SCHEDULE
Total: 4.5 years.
Preparation + ethics approval: 0.75 years.
Patient inclusion + data collection: 2.75 years.
Data analysis + reporting: 1.0 year.

Trefwoorden / Keywords

Long-term adverse effects; long-term follow-up; cancer survivorship care; screening guidelines; cost-effectiveness; Hodgkin lymphoma; non-Hodgkin lymphoma.

Samenwerking / Collaboration

Samenwerking tussen onderzoek en praktijk / Cooperation between research and practice:

Ja / Yes

Organisaties

Admiraal de Ruyter Ziekenhuis

Postbus 15
4460 AA GOES

Albert Schweitzer Ziekenhuis

Postbus 444
3300 AK DORDRECHT

Amsterdam UMC - locatie AMC
Hematologie

Meibergdreef 15
1105 AZ AMSTERDAM ZUIDOOST

Amsterdam UMC - locatie VUmc
Hematologie
CCA Room 4.24
Postbus 19268
1000 GG AMSTERDAM

Antoni van Leeuwenhoek Ziekenhuis
Plesmanlaan 121
1066 CX AMSTERDAM

Catharina Ziekenhuis
Postbus 1350
5602 ZA EINDHOVEN

Dr. B. Verbeeten Ziekenhuis
Radiotherapie
Brugstraat 10
5042 SB TILBURG

Erasmus MC Kanker Instituut
Radiotherapie
Postbus 5201
3008 AE ROTTERDAM

Haaglanden Medisch Centrum
Postbus 432
2501 CK DEN HAAG

HagaZiekenhuis
Haematologie
Postbus 40551
2504 LN DEN HAAG

Isala
Dokter van Heesweg 2
8000 GK ZWOLLE

Leiden University Medical Center
Radiotherapie
Postbus 9600
2300 RC LEIDEN

Maastricht Universitair Medisch Centrum+
Postbus 5800
6202 AZ MAASTRICHT

Maastro Clinic
Radiotherapie
Postbus 3035
6202 NA MAASTRICHT

Medisch Centrum Leeuwarden
Postbus 888
8901 BR LEEUWARDEN

Medisch Spectrum Twente
Postbus 50000
7500 KA ENSCHEDE

Nederlandse Internisten Vereniging
Postbus 20066
3502 LB UTRECHT

Nederlandse Vereniging voor Radiotherapie en Oncologie
Postbus 8176
3503 RD UTRECHT

Noordwest Ziekenhuisgroep

Postbus 501
1800 AM ALKMAAR

Radboudumc
Radiotherapie
Postbus 9101
6500 HB NIJMEGEN

Radiotherapeutisch Instituut Friesland
Borniastraat 36
8934 AD LEEUWARDEN

Radiotherapiegroep
Postbus 60160
6800 JD ARNHEM

Reinier de Graaf Groep
Postbus 5011
2600 GA DELFT

Rijnstate Ziekenhuis
Postbus 9555
6800 TA ARNHEM

St. Antonius Ziekenhuis
Interne Geneeskunde
Hematologie
Koekoekslaan 1
3435 CM NIEUWEGEIN

Stichting Hematon
Leukemie en MDS
Postbus 8152
3503 RD UTRECHT

Universitair Medisch Centrum Groningen
Hematologie
Hanzeplein 1
9713 GZ GRONINGEN

Universitair Medisch Centrum Utrecht
Inwendige Geneeskunde
Hematologie
Postbus 85500
3508 GA UTRECHT

Zuidwest Radiotherapeutisch Instituut
Koudekerkseweg 90
4382 EK VLISSINGEN

Inhoud / Content

Probleemstelling / Problem definition

THEME
This project aims to evaluate the nationwide long-term follow-up care for screening and treatment of late adverse effects of lymphoma treatment as set up by the Dutch BETER Consortium (Better care after Hodgkin lymphoma: Evaluation of long-term Treatment Effects and screening Recommendations). Therefore it fits the “follow-up” theme of this call.

INTRODUCTION/RATIONALE
Hodgkin lymphoma (HL) is a curable malignancy that occurs at young ages. Due to major improvements in treatment 5-year survival rates have increased to >85% (1). In the past, treatment consisted of radiotherapy only, whereas nowadays treatment usually involves chemotherapy with/without radiotherapy (2). For diffuse large B-cell lymphoma (DLBCL) cure rates have also substantially improved. However, cured lymphoma survivors are at increased risk of serious late complications of treatment, which may emerge from 5 years onwards. Common late effects include second malignancies (e.g. breast, lung, and gastrointestinal tract cancers), cardiovascular diseases (coronary heart disease, valvular disease, cardiomyopathy/heart failure), thyroid dysfunction, reduced fertility, premature menopause and fatigue. The risks of serious late adverse treatment effects in lymphoma survivors are substantial; e.g. the risks of second malignancy and cardiovascular disease in HL survivors

are 3- to 5-fold increased compared to the general population (3). We recently showed that, at age 65, HL survivors had a 68% cumulative risk of having developed either cardiovascular disease or second malignancy (4). Currently there are ~20,500 5-year survivors of HL and DLBCL in the Netherlands (1).
The need for long-term follow-up of subgroups of cancer survivors is being recognized. A well-organized survivorship care programme may prevent late adverse effects of treatment or detect them early, enabling early treatment and improved quality of life (5). In several countries, childhood cancer survivorship care has been implemented 10-15 years ago (6). However, structured survivorship care for late effects of lymphoma treatment was lacking. Therefore, in 2009 the Dutch BETER Consortium was established. The nationwide project group consists of haemato-oncologists, radiation oncologists, epidemiologists, psychologists and nursing specialists from 33 hospitals, as well as representatives from the Dutch Society of General Practitioners and the Dutch Haematology Patient Federation Hematon. The consortium aims to reduce morbidity and mortality from late effects in lymphoma survivors. To that end, we have developed a nationwide infrastructure of outpatient clinics where survivors are screened and treated for adverse effects of lymphoma treatment according to nationally approved BETER screening guidelines (7,8).

HEALTH CARE EFFICIENCY PROBLEM
Structured follow-up care according to nationally approved BETER screening guidelines for late adverse treatment effects for adult lymphoma survivors was first introduced in 2013-2014 in 5 BETER clinics. Currently ˜1,900 survivors have visited one of the 18 BETER clinics.
However, the efficiency of the BETER clinics has not yet been evaluated. There is a real gap in knowledge about (cost-)effectiveness of cancer survivorship programs in clinical practice (see Systematic Review). Existing studies only evaluated the yield of late effects in such programs, without a comparison group, or simulated health benefits in hypothetical cohorts (see Systematic Review). Yet, we are currently attempting to establish BETER survivorship care in all Dutch hospitals participating in BETER. The current situation with absence of BETER survivorship care in a substantial number of hospitals still allows for the conduct of a quasi-experimental study to evaluate effectiveness of BETER survivorship care. Once full nationwide implementation is in place, evaluation as proposed in this project will no longer be possible.

USUAL CARE
Before the introduction of structured survivorship care in BETER clinics, follow-up of lymphoma survivors was usually discontinued 5 years after lymphoma treatment (wait-and-see policy with regard to late adverse lymphoma treatment effects). In usual care this means that late effects like thyroid dysfunction, breast cancer, cardiovascular disease and increased risk of infections are usually not diagnosed until patients present with severe symptoms. In addition, a significant doctors' delay may occur since survivors generally present with these diseases at younger ages than the general population, often <50 years. Consequences of delays in diagnosis may include more advanced stage of disease leading to more intensive treatment, higher morbidity and mortality and increased health care costs.

(SUB-)GROUP OF PATIENTS
The patient population in this study will consist of HL survivors invited for survivorship care in BETER clinics in 2013-2016, compared to matched survivors not invited at that time, as their treatment centre had not yet started a BETER clinic.

Relevantie / Relevance

INTERVENTION TO BE INVESTIGATED
Screening and treatment of late adverse lymphoma treatment effects according to BETER guidelines (see GUIDELINE) in Hodgkin lymphoma (HL) survivors at the BETER clinics aimed at reducing morbidity and mortality from late adverse treatment effects.

EXISTING EVIDENCE OF EFFECTIVENESS
The (cost-)effectiveness of cancer survivorship care has never been assessed before in clinical practice (see Systematic Review). Benefit of screening has been demonstrated in other high-risk groups (9,10), but extrapolation of these results to cancer survivors is problematic, as adverse events caused by radiotherapy and/or chemotherapy may have a different pathogenesis. Furthermore, (cost-)effectiveness studies of cancer survivorship care have implied benefit of screening, but could not demonstrate the added value of screening in clinical practice, as they did not use a control group (10–12) or used hypothetical cohorts (13,14).

ANTICIPATED COST-EFFECTIVENESS
Based on the available data, the costs saved in health care are estimated to amount up to 4.4 million euros/year with an implementation rate of 80% (see Supplement 2). This is an underestimation of the total costs, as societal costs were not taken into account (currently unknown). The benefit of BETER survivorship care is expected to improve over time even more when survivors are all included directly after their 5-year follow-up for recurrence (average age of 33 years for HL survivors instead of 52 years in this study).

URGENCY
PRACTICE VARIATION
Currently, BETER survivorship care has been introduced in only 18 lymphoma treatment centres, due to logistic barriers involved in setting up a BETER clinic. The number of centres participating in BETER is now quickly expanding; several centres recently joined the Consortium and are planning to start a BETER clinic in 2020-2023. The current project takes advantage of this unique situation in which it is still possible to compare survivors who did and did not receive BETER survivorship care. Preliminary results of a pilot-study evaluating survivorship care within currently active BETER clinics have shown substantial differences in the frequency and the number of screening tests in practice. Some clinics see lymphoma survivors more often and/or perform more diagnostic tests than required by the BETER guidelines (7). As the optimal diagnostic test set and

frequency of screening have not, or only partly, been substantiated by scientific evidence, the proposed cost-effectiveness simulation with several scenarios for follow-up strategies based on the data generated in the proposed study will provide valuable information on which combination of screening parameters is associated with optimal outcome.

KNOWLEDGE AGENDA
Knowledge agenda Radiotherapy of the Dutch Association for Radiotherapy and Oncology (NVRO)
p.9 §3.2 Top 10 onderzoeksvragen:
• Hoe moet nazorg worden georganiseerd om de kennis van late radiatieschade te vergroten en preventie, behandeling en revalidatie hiervan te verbeteren?
p.37 §Overige kennishiaten:
• Hoe moet de follow-up worden georganiseerd?

Knowledge agenda of the Dutch Internist Association (NIV)
p.21 Wat zijn de lange termijn- en late effecten van oncologische behandeling (inclusief de impact op kwaliteit van leven)? Deze vraagstelling bestaat uit verschillende onderdelen: a. Voorkómen van lange termijn- en late effecten; b. Detecteren van en aandacht voor lange termijn- en late effecten; c. Behandelen van lange termijn- en late effecten; d. Impact van lange termijn- en late effecten op functioneren.

GUIDELINE
Before 2016, usual care for cured HL survivors was discharge from follow-up for HL recurrence after 5 years. The BETER consortium has developed guidelines for screening and treatment of the most important adverse events that occur at least 5 years after lymphoma (7). In 2016 the guidelines were approved by the respective scientific national medical societies and published on the Dutch national database for medical guidelines for secondary care and should therefore be standard of care. However, adoption of the BETER guidelines outside the BETER clinics is slow, due to organisational hurdles.

ADDED VALUE
BETER is an internationally unique initiative of an elaborate infrastructure for adult oncology survivorship care. The proposed study is not only the first evaluation of survivorship care provided at the BETER clinics but also the first such evaluation worldwide: it will result in significant information on the (cost-)effectiveness of survivorship care in screened lymphoma survivors in clinical practice. There is a lack of knowledge about the actual effectiveness of structured long-term survivorship care (see Systematic Review); the only comparable national program providing survivorship care to Dutch childhood cancer survivors (LATER) has not yet been evaluated either. The results of this study will be used to improve the BETER programme (including the BETER guidelines) where necessary and contribute to more effective, evidence-based long-term survivorship care for lymphoma and other cancer survivors.

IMPLEMENTABILITY
The BETER-project aims at implementation of survivorship care according to the nationally approved BETER guidelines for all eligible lymphoma survivors. We expect that substantiation of (cost-)effectiveness of BETER survivorship care will facilitate allocation of resources for further implementation in all lymphoma treatment centres and adherence to BETER guidelines in participating centres. Results suggesting de-implementation or adaption of the guidelines will require more scrutiny, but are easily enabled by the BETER Consortium. See IMPLEMENTATION.

SUPPORT
All BETER centres included in the established nationwide retrospective cohort of lymphoma survivors (n=27) support the current proposal. Moreover, our project proposal is in concordance with the research agenda of the NVRO and NIV. A letter of support signed by M. Stam (board member NVRO) is added (supplement 3).

PATIENT PARTICIPATION
Patient representatives from the Haematology Patient Federation Hematon actively participate in the BETER Consortium. They participate in the proposed study as advisor on patient approach, patient information and implementation of results. Members of the ‘Hematon Onderzoek Patiënten Participatie’ committee have support this proposal’s aim and feasibility and deemed it highly relevant.

DIVERSITY
The proposed study is nested in an established nationwide cohort of 5-year lymphoma survivors eligible for BETER survivorship care, allowing for assessment of BETER attendance and adherence by age, region and zip code (for socio-economic differences).

OPEN ACCESS
The BETER database is governed by the BETER consortium. Open access procedures to request and provide data for research are addressed in a regulations document that is currently being drafted. Data from the proposed study will be incorporated into the existing BETER database to accommodate FAIR data policy.

Kennisoverdracht, implementatie, bestendiging / Knowledge transfer, Implementation Consolidation

IMPLEMENTATION
We aim to assess the (cost-)effectiveness of structured survivorship care for Hodgkin lymphoma (HL) survivors in clinical practice and to identify possible areas of improvement. The results of this study will be of great interest for all stakeholders involved in cancer survivorship care and those involved in lymphoma survivorship care specifically. Involved parties include HL survivors and their health care providers, i.e. haemato-oncologists, radiation oncologists, nursing specialists, physician

assistants, cardiologists, and general practitioners.
The results of this study are also of great relevance for the population of diffuse large B-cell lymphoma survivors, who are as of 2018-2019 also being screened at the BETER clinics. The results can also be used for implementation of survivorship care for other groups of cancer survivors, such as survivors of breast cancer and childhood cancer.

When the results of this study are in favour of BETER survivorship care, more centres will be motivated to (re-)allocate resources to overcome the organisational hurdles to implement the BETER guidelines. Currently, BETER survivorship care has been introduced in 18 lymphoma treatment centres, but is still absent in other lymphoma treatment centres, due to logistic barriers involved in setting up a BETER clinic: e.g. lack of personnel, time and internal commitment and difficulties setting up the local infrastructure, such as cardio-oncology screening and referral to other specialist care. The consequence is that the approximately 30% of eligible survivors who are treated at non-participating centres, will not receive survivorship care according to the approved follow-up guidelines any time soon. However, this does not reflect the growing interest and commitment of the health care providers in lymphoma treatment centres: the number of centres interested in participating in BETER has been quickly expanding in recent years. Another contributing factor is that cardiologists are increasingly interested in the cardiovascular adverse effects of cancer treatments, reflected in the opening of several cardio-oncology outpatient clinics in University Medical Centres. We therefore expect that substantiation of the (cost-)effectiveness of BETER survivorship care will importantly contribute to implementation of and adherence to BETER guidelines, within as well as outside the BETER centres.

When the results of this study indicate that one or more of the BETER guidelines are not effective or need improvement, the BETER consortium will establish a working group consisting of members of the BETER consortium and additional relevant specialists (e.g. cardiology). Results suggesting de-implementation or adaptation of the guidelines will require further scrutiny: evidence from the proposed study will be systematically assessed and graded together with additional evidence collected from literature and recommendations by experts. After internal consent, proposals for adapted guidelines or de-implementation will be sent to the respective scientific national medical societies for approval.

Furthermore, reimbursement of the BETER survivorshipcare was negotiated with the responsible authorities (Nederlandse Zorgautoriteit), resulting in a DOT-DBC (available since 2016). If the BETER programme is significantly adapted based on the current proposal, the DOT-DBC will also have to be adapted.

The knowledge gained in this project about late adverse effects and risk perception of late effects of lymphoma treatment in HL survivors will be used to optimize our patient information facilities (i.e. our website for patients www.beternahodgkin.nl). This will hopefully also improve attendance at the BETER clinics and adherence to the BETER guidelines.
Furthermore, to increase attendance and adherence, and to report on our cost-effectiveness results, we will give presentations in Dutch hospitals (for survivors as well as treating physicians) and at conferences, and we will write Dutch-language as well as international publications.

BACKGROUND IP
Not applicable; although the intervention has not been implemented at the national level, the intervention is formally standard of care since the acceptance of the BETER guidelines in 2016.

Doelstelling / Objective

OBJECTIVE
We aim to assess the (cost-)effectiveness of structured survivorship care for lymphoma survivors in clinical practice and to identify possible areas of improvement. More specifically, we wish to examine whether survivorship care at the BETER clinics is effective in reducing morbidity and mortality from late effects of Hodgkin lymphoma (HL) treatment and associated costs, and whether it will increase health-related quality of life and decrease health-related productivity losses.
The cost-effectiveness analysis will be performed from a healthcare and societal perspective of the Netherlands, using a short term (based on current study) and long term/lifetime (based on extrapolations) time horizon. A budget impact analysis will be performed to estimate the 5-year financial consequences of adoption and diffusion of the BETER care in the Dutch healthcare system.
We will also assess adherence to the guidelines, both from physicians and patients in order to identify possible areas of improvement of BETER survivorship care in clinical practice. Moreover, attendance rates to the BETER clinics will be scrutinized and reasons not to attend will be assessed or inferred from patient characteristics for those who do not attend.
We also aim to assess whether BETER care increases knowledge about late adverse effects and affects risk perception of and distress about late effects.

HYPOTHESIS
Personalised, risk-based screening for and early treatment of adverse events in HL survivors, according to the BETER guidelines, leads to reduced burden of disease from late effects, lower healthcare costs, better quality of life and reduced productivity loss compared to the absence of structured survivorship care.

RESEARCH QUESTION
Compared to usual care (absence of structured survivorship care), is survivorship care at the BETER clinics effective in 1) reducing morbidity and mortality from late effects of lymphoma treatment and associated costs; 2) increasing health-related quality of life and 3) reducing productivity losses?
What are the adherence rates to the BETER guidelines from patient and health care provider perspectives and what specific (parts of the) guidelines have lower adherence?
Which patient characteristics are associated with lower attendance rates to the BETER clinic?

Is knowledge about late adverse effects, risk perception of and distress about late effects associated with BETER clinic attendance and adherence to the screening guidelines?

Plan van Aanpak / Strategy

1. DESCRIPTION OF CLINICAL STUDY

1.1. DESIGN
The proposed study is a retrospective cohort study with a quasi-experimental design. The intervention group consists of Hodgkin lymphoma (HL) survivors who were invited to first visit a BETER clinic in 2013-2016 and the control group consists of survivors eligible for BETER survivorship care as of 2013-2016, but treated in hospitals starting a BETER clinic later, in 2021-2024. In this design we make use of the gradual introduction of BETER survivorship care in the Netherlands, as not all centres participating in the BETER consortium were able to provide BETER care immediately. This was because it took time to identify and trace the eligible patient population and set up the local infrastructure, such as for referral to cardio-oncology and other specialist care.
For the conduct of the study, we also have the unique opportunity to use a previously established comprehensive data set from a large nationwide retrospective cohort of all lymphoma survivors treated in 27 BETER centres between 1965-2012, with patient characteristics, treatment data, and outcome data (such as dates of diagnosis of second cancers, cardiovascular events, date and cause of death). This allows selection of a control population of individuals who were not yet screened during 2013-2016, frequency matched to the intervention group on age and year of lymphoma diagnosis, and lymphoma treatment. Additional follow-up data for all included survivors will be collected through disease registries and survivors' general practitioners without burdening the study population. The comprehensive dataset enables comparison of the intervention and control group and assessment of characteristics of non-responders to BETER survivorship care.

1.1.1. Considerations about other study designs: The ideal design to evaluate the (cost-)effectiveness of BETER survivorship care would have been a randomized controlled trial (RCT), randomizing patients to receive either BETER care or no BETER care (which generally would imply no survivorship care at all). Such a design is not possible because it was deemed to be unethical to withhold BETER care from eligible survivors when BETER care was introduced, as their risks of late adverse effects are high and the benefits of screening for breast cancer and cardiovascular disease (CVD) have been demonstrated in other high-risk groups (9,10) and implied by single screening modality studies in lymphoma survivors (11,12). Moreover, when the screening recommendations developed by the BETER consortium were approved by the respective scientific national medical societies, health care professionals were bound to adhere to them.
Alternatively, an effectiveness study with a stepped wedge design was considered, randomizing centres between immediate introduction of BETER care or waiting several years, while BETER care was gradually introduced. However, again on ethical and regulatory grounds, this was not feasible because centres did not want to postpone initiation of BETER survivorship care when they were ready to start. A pre-test post-test control group design, also called the classic controlled experimental design, was not possible either, as baseline measurement in both patients in BETER participating centres and centres not yet participating could not be performed: most survivors eligible for BETER were discharged many years ago and treating physicians considered it unethical to invite them back to the hospital for a baseline assessment only, without subsequently offering them survivorship care according to the BETER guidelines.
In summary, an RCT, a stepped wedge design and a pre-test post-test control group design evaluating BETER survivorship care versus absence of structured survivorship care were not feasible due to ethical and regulatory objections.

1.2. PATIENT (P)
1.2.1. Patient population
The study will be nested in a large nationwide retrospective cohort study of HL survivors treated in 27 Dutch centres between 1965 and 2012 (15,16). This cohort was originally established in the 1980s-1990s to examine the late effects of HL treatment; since then the cohort has been periodically extended with more treatment centres, patients diagnosed in more recent years and with diffuse large B-cell lymphoma (DLBCL) survivors diagnosed between 1989 and 2012. Lymphoma survivors eligible for BETER survivorship care were successfully treated at least 5 years ago for HL or DLBCL at age 15-60 years and are less than 70 years of age at invitation. The patient population in this evaluation study will only consist of HL survivors eligible for BETER survivorship care, as they were the first to be invited to the BETER clinics in 2013-2016 due to their higher risk of late adverse treatment effects compared to DLBCL survivors.

1.2.2. Intervention group: the first 450 HL survivors between 2013 and 2016 who were invited for screening for late adverse treatment effects in the BETER centre where they were originally treated for HL. With attendance rates ranging between 60-70%, around 300 HL survivors in the intervention group are expected to receive screening at the BETER clinics.

1.2.3. Control group: frequency matched group of 450 HL survivors, eligible for BETER screening for late adverse treatment effects in 2013-2016, but not yet invited because the centre where they were originally treated for HL does not start a BETER clinic until 2021-2024. So, the control group will be invited in 2021-2024.
Controls will be matched with the intervention population on the following criteria:

- sex
- age at diagnosis (±3 years)
- year of diagnosis (±3 years)
- lymphoma treatment characteristics associated with increased risk of CVD, breast cancer, hypothyroidism and functional asplenia (see also 1.3.1.). In practice this implies matching on chest radiotherapy (y/n), neck radiotherapy (y/n), spleen radiotherapy (y/n), splenectomy (y/n) and anthracyclines (y/n + doxorubicin =300 mg/m2 y/n).

Survivors selected as controls should be alive at the time the corresponding survivor in the intervention group was invited to

come to the BETER clinic. Of note, matched controls eligible for BETER screening in 2013-2016 who died during follow-up, prior to invitation for BETER screening in a BETER clinic 2021-2024, are eligible for our study.

1.3. INTERVENTION (I)
The BETER guidelines provide personalised risk-based screening recommendations according to the risk of late adverse treatment effects associated with a survivor’s sex, current age, age at lymphoma diagnosis and lymphoma treatment (1.3.1.). The intervention in the proposed study consists of BETER survivorship care, i.e. personalised risk-based screening for and treatment of CVD (including risk factors for CVD), breast cancer and hypothyroidism, and infection prevention measures (incl. vaccination) in case of functional asplenia. BETER survivorship care also includes lifestyle recommendations (see 1.3.1.) and is administered in clinical practice at an outpatient clinic in a BETER participating centre (further denoted as BETER clinic).

1.3.1. BETER Screening Recommendations for Late Adverse Effects after Lymphoma Treatment

1.3.1.1. Cardiovascular Disease

RISK: standardized incidence ratio (SIR) of 2-6 and a 40-year cumulative incidence of 50% (at age 65) (16).

SUBGROUP OF LYMPHOMA SURVIVORS: treated with cardiotoxic chemotherapy (doxorubicin, epirubicin, rubidomycin, daunorubicin, mitoxantrone) with cumulative doses equivalent to doxorubicin =300 mg/m2, radiotherapy to the mediastinum or radiotherapy to the mediastinum in combination with cardiotoxic chemotherapy, independent of cumulative dose.

SCREENING: screening of cardiovascular risk factors every 5 years, up to age 70 (medical history, physical examination and blood tests: lipid spectrum, glucose, biomarkers BNP or NTproBNP as a reference for follow-up), screening for valvular disease and subclinical heart failure by cardiac ultrasound only once 15 years after diagnosis in case of radiotherapy mediastinum and every 5 years in case of cardiotoxic chemotherapy (with/without mediastinal radiotherapy) and ECG (once as a reference).

TREATMENT AND/OR PREVENTION OF LATE ADVERSE EFFECTS: treatment of cardiovascular risk factors hypertension and hypercholesterolemia, treatment in case of subclinical decrease of ejection fraction and lifestyle recommendations in accordance with general population guidelines.

1.3.1.2. Breast Cancer

RISK: SIR 4-6 and a 30-year cumulative incidence of 17-26% at age 55 (15).

SUBGROUP OF LYMPHOMA SURVIVORS: women treated with radiotherapy on chest and/or axillae before the age of 40 years.

SCREENING: age 25-30: annual clinical breast examination and magnetic resonance imaging (MRI); age 30-60: annual clinical breast examination, mammography and MRI; age 60-70: biennial clinical breast examination and mammography; age 70-75: biennial mammography through population screening.

TREATMENT AND/OR PREVENTION OF LATE ADVERSE EFFECTS: take previous chest radiation treatment into account and consider risk of cardiomyopathy when using anthracyclines.

1.3.1.3 Thyroid Dysfunction

RISK: absolute risk of 44-67% 25 years after treatment (3).

SUBGROUP OF LYMPHOMA SURVIVORS: treated with radiotherapy to the thyroid region.

SCREENING: every 1-3 years palpation of the thyroid gland and annual laboratory examination of thyroid-stimulating hormone; if abnormal free tetra-iodothyronine (FT4).

TREATMENT AND/OR PREVENTION OF LATE ADVERSE EFFECTS: in accordance with general population.

1.3.1.4 Overwhelming Post-Splenectomy Infections

RISK: relative risk up to 20 (17).

SUBGROUP OF LYMPHOMA SURVIVORS: treated with splenectomy, splenic radiotherapy (mean dose >20 Gy) or stem cell transplant.

SCREENING: none.

TREATMENT AND/OR PREVENTION OF LATE ADVERSE EFFECTS: vaccinations for Pneumococcus (start with Prevenar-13, followed by Pneumovax-23 two months later; Pneumovax-23 every 5 years), Haemophilus Influenzae B once, Meningococcus once, Influenza annual; medical alert card, travel advice and antibiotics on demand.

1.4 COMPARATOR (C)
Absence of structured survivorship care after discontinuation of follow-up for lymphoma recurrence (patients were usually

discharged 5 years after lymphoma treatment).

1.5 FOLLOW-TIME (T)
To facilitate follow-up of at least 5 years at the BETER clinics, the follow-up of lymphoma survivors in the intervention group starts in 2013-2016, when they were first invited to attend the BETER clinic, and ends in 2021-2024 at their most recent BETER visit. The start of follow-up of survivors in the control group will correspond to that of the intervention group and ends at their first BETER visit in 2021-2024. The follow-up of survivors who do not attend the BETER clinic (i.e. non-attenders in both the intervention and control groups) ends on completion of the mailed questionnaires. Furthermore, if survivors either in the intervention group or comparison group die during follow-up, follow-up ends at death. Expected median follow-up time is 8 years.

1.6 OUTCOME (O)

1.6.1 Primary
1) (Cost-)effectiveness of structured survivorship care at the BETER clinics in terms of:
a. Burden of disease in disability adjusted life years (DALYs) from CVD (including risk factors for CVD), breast cancer, hypothyroidism and functional asplenia
b. Health care costs related to CVD (including risk factors for CVD), breast cancer, hypothyroidism and functional asplenia
c. Health-related quality of life (HRQoL) and QALY's using the 36-item Short Form Health Survey (SF-36) and the 5 dimension/5-level EuroQol (EQ-5D-5L) questionnaire
d. Health-related productivity losses using the iMTA Productivity Cost Questionnaire (iPCQ).
2) The yield of previously undiagnosed adverse events from screening and the incidence of adverse events during follow-up
a. At prevalent and incident screening of survivors attending the BETER clinic (for those survivors in the intervention group who are attending the BETER clinic)
b. From hospital records, Netherlands Cancer Registry, The National Heart Registry, GP records, Dutch Hospital Data registry, and BETER questionnaire: for all included survivors, to allow valid comparison between the intervention and comparison groups

1.6.2 Secondary
1) Adherence to the BETER guidelines by
a. HL survivors
b. health care providers
2) (Non-)attendance at the BETER clinics
3) Health care use in both groups, incl. screening practices in survivors who do not attend the BETER clinic between 2013-2016 and 2021-2024
4) Knowledge about late adverse effects and risk perception of late effects of lymphoma treatment
5) Distress about late effects of lymphoma treatment

1.6.1.1. Assessment of Yield of Screening and Incidence of Adverse Events
For the primary outcomes, we will first determine the yield of previously undiagnosed adverse events during follow-up, i.e. between 2013-2016 and 2021-2024. This information will be extracted from hospital medical records, the survivors' general practitioners, disease registries and the self-administered BETER questionnaire.
Before their clinic visit survivors are asked to complete a questionnaire on their current health, medical history, and lifestyle. This so-called BETER questionnaire helps healthcare providers to focus on actual symptoms of late effects and on lifestyle during the consultation. The questionnaire contains items on general background (e.g. marital status, level of education), medical history (adverse events, including reproductive history and fatigue), family history, medication, screening history, physical activity, weight, alcohol, smoking and recreational drug use, as well as psychosocial problems, difficulties with employment, insurance coverage, and mortgages. The BETER questionnaire will also be mailed to eligible survivors who decided not to visit a BETER clinic. In line with the intervention group, the survivors in the control group will fill in the BETER questionnaire at their first BETER visit in 2021-2024 or through mail (for non-responders). The BETER questionnaire thus provides the proposed project with important information on existing morbidity/diseases prior to the first BETER visit and on characteristics of the BETER visitors. The response to The BETER questionnaire is very high, ranging between 90-95%.

For the intervention group trained data managers will access the BETER medical records in the BETER clinics and collect the relevant data from all BETER visits, including all screening/diagnostic tests during follow-up and their results. For all survivors included in the study (intervention and comparison groups) we will obtain information on the incidence of clinical events of interest through linkage with disease registries. Information on second malignancies will be obtained from The Netherlands Cancer Registry, information on cardiac interventions will be obtained from The National Heart Registry, information on hospital admissions for CVD and serious infections (overwhelming post-splenectomy infections) will be obtained from Dutch Hospital Data and cause of death for those who died during follow-up will be obtained from Statistics Netherlands. We will also approach the general practitioners of all patients to obtain data on CVDs (especially those not requiring hospital admission, such as angina pectoris), cardiovascular risk factors (hypertension and hypercholesterolemia) and hypothyroidism.
Survivors' informed consent for the above data collection is asked at the first visit to a BETER clinic. For included survivors who choose not to visit a BETER clinic informed consent for linkage with disease registries and approach of general practitioner is requested by mail. Currently over 97% of all patients visiting a BETER clinic has provided consent. Informed consent for the control group will be obtained during the patients' first visit at a BETER clinic in 2021-2024 and is not necessary for deceased individuals.

1.6.1.2. Assessment of Burden of Disease
In the proposed study, burden of disease from morbidity and mortality from CVD (including risk factors), breast cancer,

hypothyroidism and overwhelming post splenectomy infections due to functional asplenia is measured in 'disability adjusted life-years' (DALYs). One DALY can be thought of as one lost year of "healthy" life. DALYs for a disease or health condition are calculated as the sum of the 'years of life lost' (YLL) due to premature mortality and the 'years lost due to disability' (YLD) for people living with the health condition or its consequences: DALY = YLL + YLD. The YLL correspond to the number of deaths multiplied by the standard life expectancy at the age at which death occurs. To estimate YLD for a particular health condition in a particular time period, the number of cases in that period is multiplied by the average duration of the disease (in years) and a weight factor (disability weight) that reflects the severity of the disease on a scale from 0 (perfect health) to 1 (dead). Disability weights are extracted from literature (18–21).

1.6.1.3. Assessment of Health care costs
In order to calculate the actual health care costs incurred in both groups during the study follow-up, i.e. between 2013-2016 and 2021-2024, the screening and treatment costs associated with breast cancer, CVD (including risk factors for CVD), hypothyroidism and functional asplenia will be assessed based on a combination of the available standard tariffs. See also 2.2. Cost analysis.

1.6.1.4. Assessment of Health-related quality of life (HRQoL)
In all survivors HRQoL will be assessed at end of follow-up in 2021-2024 using the Short Form Health Survey, a 36-item, self-reported survey of patient health (SF-36), and the 5 dimension/5-level EuroQol (EQ-5D-5L) questionnaire. The SF-36 assesses HRQoL in 4 physical domains (physical functioning, role limitations due to physical problems, bodily pain, general health) and 4 mental domains (social functioning, role limitations due to emotional problems, mental health and vitality) with points ranging from zero to an optimum score of 100 points (22). From these domains a physical and mental component score (PCS and MCS) can be derived. The EQ-5D-5L assesses health-related problems in five domains (mobility, self-care, activities of daily living, pain/discomfort and anxiety/depression) to produce a utility value ranging from 0 (death) to 1 (perfect health) (23). Survivors who have declined the invitation to visit a BETER clinic will also be asked to fill in this questionnaire. Survivors in the control group will be asked to fill in these questionnaires before their first BETER visit.

1.6.1.5. Assessment of Health-related productivity losses
To measure and subsequently value indirect costs that arise outside the scope of the health care system, we will use the iMTA Productivity Cost Questionnaire (iPCQ) to assess health-related productivity losses (24). The iPCQ includes 3 modules measuring productivity losses of paid work due to absence from work, reduced productivity while at work and productivity losses related to unpaid work. This questionnaire will be administered at the end of follow-up in 2021-2024. Survivors who have declined the invitation to visit a BETER clinic will also be asked to fill in this questionnaire. Survivors in the control group will be asked to fill in this questionnaire before their first BETER visit.

1.6.2.1. Assessment of Adherence to the BETER guidelines
Adherence to the BETER guidelines by patients and health care providers will be assessed during follow-up between 2013-2016 and 2021-2024. We will assess whether survivors have followed recommendations for referral to medical specialists or to their GP (for cardiovascular risk management). Both for survivors and BETER health care providers we will assess whether the screening frequency during follow-up is adhered to.

1.6.2.2. Assessment of (Non-)Attendance at the BETER clinics
Preliminary data from a small study in a few BETER clinics show that 60-70% of the HL survivors who were invited attended the BETER clinic. We found that especially survivors who were no longer under medical surveillance at the BETER centre were less likely to attend. The most common reasons not to attend were having already been diagnosed with adverse events elsewhere and unwillingness to attend (e.g. due to emotional burden). A possible reason for declining may be that survivors do not want to become a lifelong patient instead of a cancer survivor. Also, financial considerations may play a role, as Dutch healthcare insurance includes a mandatory deductible sum of 385 euros as of 2016, which not everyone is able or willing to pay. More detailed evaluation of (non-)attendance in more BETER clinics may reveal the need for additional implementation measures to improve the uptake of the programme. Survivors who have declined the invitation to visit a BETER clinic will also be asked to fill in the BETER questionnaire. The BETER questionnaire contains items that assess a survivor's health care use and screening history: what, when, how and where (at which hospital/GP) is screened for. This will help us to get a better picture of the patient characteristics and current health issues of the non-attending survivors. It will also provide information on which part of these survivors is already partly screened according to (some of) the BETER guidelines. To elucidate possible financial reasons for (non-)attendance, we will infer social economic status from postal code and assess whether it correlates with attendance rate, as financial considerations about the mandatory deductible sum in health care insurance may play a role in declining the invitation.

1.6.2.3. Health Care Use, incl. Screening Practices
Information on the overall health care use (medication, GP visits, hospital visits and hospitalisation) between 2013-2016 and 2021-2024 in the intervention and control groups will be extracted from hospital medical records, the survivors' general practitioners, disease registries and the self-administered BETER questionnaire. It will allow us to compare overall health care use between the intervention and control groups, as well as compare survivors who have accepted and declined to attend the BETER clinic. This will provide more insight into the patient characteristics of those who declined to attend the BETER clinic: e.g. are they not experiencing any health complaints or is their health compromised and are they treated/screened for late treatment effects elsewhere? See also 1.6.2.2.

1.6.2.4. Assessment of Knowledge about Late Adverse Effects and Risk Perception of Late Effects
Despite the fact that in recent years increasing attention has been given to the need for follow-up care, cancer survivors are often unaware of their risk of treatment-related adverse effects, which may delay diagnosis and treatment of these adverse effects (25). In childhood HL survivors it was shown that increased awareness of possible late effects may influence the health

behaviour of cancer survivors and increase participation rates in screening programs (26). This underlines the importance of increasing awareness and knowledge about treatment-related adverse effects in lymphoma survivors. In the proposed project, knowledge about treatment-related adverse effects will be examined with a slightly adapted version of a questionnaire CCSS (25) in both the intervention and control groups. Most items consist of statements covering the wide range of treatment-related adverse effects and the level of risks after HL treatment, including knowledge about the need for screening. Survivors who have declined the invitation to visit a BETER clinic will also be asked to fill in this questionnaire. Survivors in the control group will be asked to fill in this questionnaire before their first BETER visit.

1.6.2.5. Assessment of Distress about Late Effects
Detailed information on late effects could cause increased levels of distress in survivors. We therefore want to assess the effect of BETER survivorship care on worries about treatment-related adverse events and will ask survivors in the intervention and control group to fill in a 6-item questionnaire based on the Cancer Worry Scale (CWS) (27). The scale assesses frequency of concerns about developing treatment-related adverse effects and the impact of these concerns on mood and daily functioning on a 4-point scale. Survivors who have declined the invitation to visit a BETER clinic will also be asked to fill in this questionnaire. Survivors in the control group will be asked to fill in this questionnaire before their first BETER visit.

1.7. SAMPLE SIZE CALCULATION

1.7.1. Assessing the potential health gains
To assess the potential health gains (in terms of averted DALYs) of BETER survivorship care, a model was constructed in which the burden of disease accumulated during 5-year follow-up (note: the follow-up in the proposed study is expected to be longer) was calculated for the intervention group, screened at a BETER clinic, and the control group. The model includes cumulative incidence of the late adverse events after treatment under study: (risk factors of) CVD, breast cancer, hypothyroidism and overwhelming post-splenectomy infections due functional asplenia. The risk of these conditions is substantially increased: see 1.3.1. BETER Screening Recommendations for Late Adverse Effects after Lymphoma Treatment. The impact of treating the CVD risk factors high blood pressure and hypercholesterolemia on developing CVD and death from CVD was inferred from risk reduction tables published by the Dutch Society of General Practitioners (www.nhg.org/sites/default/files/content/nhg_org/uploads/cvrm_protoc_bijlagen_web_13.pdf). Disability weights for CVD were extracted from the Global and Dutch Burden of Disease studies (19,21). The number of DALYs averted by breast cancer screening was based on a cost-effectiveness study on breast cancer interventions (28). Based on this model, we estimated that the average HL survivor of 52 years of age, who is not screened for late adverse treatment events, acquires 1.18 DALYs in the next 5 years (1.12 for breast cancer + CVD; 0.01 for hypothyroidism; 0.04 for functional asplenia), as opposed to 0.56 DALYs for a survivor screened at a BETER clinic (0.55 for breast cancer + CVD; 0.00 for hypothyroidism; 0.00 for functional asplenia). [Of note, the numbers do not add up due to rounding.] We therefore assume that, at patient level, screening at the BETER clinics averts on average 0.62 DALYs per survivor in a 5-year period. In the 1.8. DATA-ANALYSIS section we describe that we will perform an ‘intention to screen’ analysis, assessing the impact of BETER survivorship care including survivors who choose not to attend. Preliminary data from a small study in a few BETER clinics showed an attendance rate of 60-70%. When we take an attendance rate of 65% into account, the expected difference between the intervention and the control group is 0.40 DALY: 0.78 DALYs in the intervention group (65% of 0.56 DALYs + 35% of 1.18 DALYs) and 1.18 DALYs in the control group.

1.7.2. Sample size calculation
In the ‘intention to screen’ analysis, we expect a 5-year accumulation of 0.78 DALYs in the intervention group and 1.18 DALYs in the control group. With a 2-sided significance level of 0.05 and 80% power to detect a difference of 0.20 standard deviation (small effect size) and larger, we need 393 patients in each study arm. When we take 15% loss to follow-up and incomplete data into account, we need 452 patients in each study arm.

1.7.3. Effect size for other analyses
Now that we have powered for the ‘intention to screen’ analysis, what are the effect sizes we can detect in other analyses? For the patient level analysis we anticipate only 7% incomplete data (as these survivors all are or have been attending a BETER clinic). With an attendance rate of 65% we anticipate 274 survivors at patient level analysis in each arm (65% of 452/1.07). This allows us to detect a difference of at least 0.24 standard deviation (small effect size) with an expected difference of 0.62 DALYs between intervention and control group, a 2-sided significance level of 0.05 and 80% power.
For important sub-analyses, comparing for example the yield/incidence of the different CVD outcomes or subgroups according to age (above/below 50 years) or sex, the proposed sample size provides 80% power to detect medium effect sizes (0.50 standard deviation). These analyses are relevant to provide information on the effectiveness of specific guidelines, and because guidelines are different for males and females (regarding breast cancer screening) and different age groups.

1.8. DATA-ANALYSIS

1.8.1. Intention to screen analysis
To assess the impact of BETER survivorship care on the centres’ eligible lymphoma population, the outcome parameters will be compared between the first 450 survivors who were invited to a BETER clinic that started in 2013-2014 and 450 matched survivors who were treated for lymphoma at BETER centres that does not start a clinic until 2021-2024. Reasons why participating centres did not start a BETER clinic before 2021 are of logistic nature involving identification and tracing of patients and setting up the infrastructure, especially the availability of an interested cardiologist and a nurse-practitioner; factors very unlikely to be associated with treatment-specific risks of late effects and our study outcomes. Centres starting an early BETER clinic included both University Medical Centres and large peripheral hospitals. This allows us to use an instrumental variable analysis with the variable ‘treated for lymphoma at a centre with a BETER clinic that started in 2013-2014’ (y/n) as instrumental variable. We hereby mimic randomization and minimalize the risk of bias in this observational study. The variable ‘treated for lymphoma at a centre with a BETER clinic that started in 2013-2014’ is strongly associated with the likelihood of

receiving the intervention (65% vs. 0-5% for clinics that started in 2013-2014 and 2021-2024 respectively), but not associated with any prognostic factor or with the outcome (other than through the intervention). The attendance rates are based on preliminary data from a small study in a few BETER clinics which showed that the attendance rate was 60-70% and that survivors only rarely visited a BETER centre not connected to their original lymphoma treatment centre.
Individual matching of control group participants to the intervention group on relevant treatment characteristics will control for any differences in lymphoma treatment between centres.

When we select the matched comparison group eligible for BETER care in 2013-2016, we do not yet know whether they will attend the BETER clinic when invited in 2021-2024; therefore, in a truly unbiased analysis, attendance should not be an eligibility criterion in the intervention group as well. Our intention-to-screen analysis is therefore the most valid approach to evaluation of the cost-effectiveness of BETER survivorship care.

1.8.2. Patient level analysis
To assess the impact of BETER survivorship care on screened/treated individuals, outcome parameters will be compared between the first 270 survivors who attended a BETER clinic that started in 2013-2014 and matched survivors who are not (yet) screened and were treated for lymphoma at BETER centres that start a clinic in 2021-2024.

On both levels of analysis, multiple linear regression analysis with will be used for continuous outcomes (such as DALYs and health care costs) and Poisson or negative binomial regression analysis will be used for count variables (i.e. yield of previously undiagnosed adverse events). The regression analyses will also take the amount of follow-up time into account, thereby correcting for a possible difference in follow-up time in the intervention and control group.

2. COST EFFECTIVENESS ANALYSIS (CEA)

2.1. General considerations
A cost-effectiveness analysis will be performed comparing costs and effects of “BETER care” versus “no BETER care”. The analysis will be performed from a healthcare and societal perspective of the Netherlands, using a 5-year (based on data from the current study) and long term/lifetime (based on extrapolations by means of data from literature) time horizon. In the study, we will derive information on proportions- and types of late effects occurred in an early or late stage after their initial treatment. We expect that screening will either find late effects in an earlier stage (which will presumably lead to lower healthcare costs and complaints, and higher QoL), or even will prevent late effects from occurring.

2.2. Cost analysis
Direct and indirect healthcare costs will be gathered in the proposed study, extracted via patient files (direct healthcare consumption), and productivity losses via the questionnaires (indirect costs), as described above. Benchmark costs from the Dutch NZA or costs from the handbook of costing studies from the Dutch ZorgInstituut will be used if possible; otherwise, an Activity Based Costing method will be performed for health care consumption which is unknown.

2.3. Patient outcome analysis
Effects will be expressed in costs/DALYs and costs/QALYs obtained from the proposed study, and additional literature.
For the DALYs, we will use the incidence/prevalence, frequency and severity of morbidities in the study to obtain the years less lived (YLL). To calculate the years lived with disease (YLD), we will use disease weight based on literature (18–21,28).
For the QALYs, we will use utilities by means of the EQ-5D-5L for both intervention and control groups at the end of follow-up in 2021-2024.
Long term consequences can be partly based on the current study, in combination with additional literature to extrapolate the effects for life time.

2.4. Cost-effectiveness Analysis
The cost-effectiveness analysis will be performed by means of a discrete event simulation, to take the factor “timing” into account and because patient level data is available. In the cost-effectiveness analysis, we will incorporate several scenarios for follow-up strategies, based on the data generated in the proposed study.
The results of the cost-effectiveness analysis will be incremental costs (or savings) of the BETER care per QALY gained. The CEA will be performed according to the guidelines for economic evaluations of the Dutch Zorg Instituut. State of the art health economic methods will be applied. Uncertainty around the results will be quantified by means of non-parametric bootstrapping and cost-effectiveness acceptability curves, showing the possibility of the BETER care being whether or not cost-effective for various values of the Dutch society willingness to pay for one QALY.

3. BUDGET IMPACT ANALYSIS (BIA)

3.1. General considerations
A BIA will be performed to estimate the 5-year financial consequences of adoption and diffusion of the BETER care in the Dutch healthcare system. The following points will be addressed: the proposed intervention, the potential number of patients that are eligible for the intervention, costs per patient, treatments, palliative care. The guidelines from the Dutch ZorgInstituut and ISPOR 2016 will be followed for the design and execution of the BIA.

3.2. Cost Analysis
The costs gathered for the Cost-effectiveness analysis will be used applicable for the BIA.

4. SYSTEMATIC REVIEW

4.1 Search Strategy
Publications about the effectiveness of survivorship care for survivors of HL or childhood cancer were identified by a structured search strategy. We searched the databases of Pubmed, Embase (Ovid) and Scopus for relevant publications till February 19, 2020. The search strategies that were used can be found in Supplement 6.

Although our project aims to evaluate the effectiveness of survivorship care for HL survivors, we also selected literature about the effectiveness of such care for childhood cancer survivors. This was done because we expected there would be very little literature on HL and possibly a bit more for childhood cancer, as the establishment of survivorship care for HL is quite recent, while such care for childhood cancer survivors has been initiated around 2005-2010. We assumed that results from studies in childhood cancer survivors could also be relevant for our research question.
To assess whether a publication was relevant we applied the following criteria:
1. The study should include screening (surveillance) for long-term adverse effects of cancer treatment;
2. The study population should consist of (at least) 5-year survivors of lymphoma or childhood cancer;
3. The studied intervention should be (one of) the screening methods/interventions used in BETER, i.e. screening and treatment for breast cancer, CVD, cardiovascular risk factors, thyroid dysfunction or vaccination for asplenia (spleen dysfunction);
4. The study should include a control group receiving usual care (or no care);
5. The outcome of the study should not (only) be the diagnostic accuracy of the screening method but (also) effectiveness and/or cost-effectiveness of the screening/survivorship care program;
6. The study outcome measure(s) should include one or more of our primary outcome measures: the burden of disease or yield of one or more late adverse cancer treatment effects, or health care costs;
7. The study outcome(s) should be assessed in clinical practice; therefore simulation studies modeling effectiveness of cancer survivor care programs based on a simulated cohort of cancer survivors or on data for other (non-cancer) high-risk groups were excluded.

4.2. Results
639 titles were retrieved about survivorship care for HL survivors (315 from Pubmed, 194 from Embase(ovid) and 130 from SCOPUS) and 176 titles were retrieved for childhood cancer survivorship care (98 from Pubmed and 45 from Embase(ovid) and 33 from SCOPUS). After removing 269 duplicates, 546 publications were assessed for eligibility.
First, titles and abstracts of publications identified by the search strategy were independently screened by 2 reviewers (Annelies Nijdam, Floor van Leeuwen). Second, full-text papers of all selected publications were retrieved. Both reviewers independently assessed each retrieved full-text paper to determine whether it met the inclusion criteria.
Based on titles and abstracts, 31 titles were obtained in full text, 26 for HL and 5 for childhood cancer. If the 2 reviewers disagreed with respect to the eligibility of a title/abstract, we always obtained and assessed the full text. The most important reason for exclusion of titles was that studies only reported on the risk of long-term adverse events (recommending screening for late effects in their conclusion), but did not examine the effect of an intervention to reduce the risk. Other studies were excluded as they studied the diagnostic accuracy of screening interventions or studied screening interventions not (yet) part of the BETER screening program.

After closer inspection of the 31 publications obtained in full text, it turned out that none of the publications met the inclusion criteria. Most frequent reasons for ineligibility of papers were:
1. Description of screening program, no results;
2. Intervention did not correspond to (one of) the screening methods/interventions used in BETER survivorship care;
3. Simulation study, no data from clinical practice;
4. No comparison group;
5. Reported outcome measures did not correspond with one of the primary outcomes in our proposed study; e.g. only participation rates or diagnostic accuracy of one of the BETER screening methods were reported.
All publications obtained in full-text were hand-searched for additional relevant references; this yielded 8 potentially relevant papers.

We also searched ClinicalTrials.gov, a website which provides a worldwide overview of ongoing clinical studies that are currently recruiting patients and have not yet been published. This website is maintained by the National Library of Medicine and the National Institutes of Health and has information on publicly and privately supported clinical studies on a wide range of diseases and conditions. We did not identify any ongoing studies examining the effectiveness of survivorship care (or screening for late adverse effects) in survivors of HL or childhood cancer.

4.3. Synthesis of evidence

4.3.1. Signorelli et al. (2017)
The most promising publication was a systematic review published on the impact of long-term follow-up care for childhood cancer survivors by Signorelli et al. in 2017 (29). The authors assessed impact of engagement in specialized survivorship care on survivors' medical and psychosocial outcomes. Nine eligible articles were found. The majority of studies focused on knowledge about late effects (n=4), risk perception of late effects (n=4), general health care use and use of support services (n=4), and number of late effects detected (n=4; no comparison group).
Although none of the papers addressed the (cost-)effectiveness of survivorship care in terms of reduced morbidity and/or mortality from late effects, we briefly describe here the most important results of this systematic review. Survivors attending follow-up care tended to demonstrate higher knowledge about their treatment and diagnosis (n = 2), and had more accurate late effects risk perception (n = 3). Attendees engaged in increased more regular surveillance, had fewer emergency department visits/hospitalizations (n = 1), and more late effects detected (n = 3), than non-attendees. No signi?cant differences were observed between attendees and non-attendees in psychological morbidities, including post-traumatic stress disorder

(30). Although not signi?cant either, attendees tended to report higher distress levels than non-attendees. Survivors with moderate/severe late effects who were disengaged from follow-up, had signi?cantly lower health-related quality of life than survivors under surveillance with no/mild late effects, worst on physical and social functioning, vitality and general health perceptions scales (31).
Signorelli and colleagues concluded that there is a dearth of literature systematically evaluating the medical and psychosocial impact of follow-up care.

We identified a few papers with results that had some relevance for our research questions, although none of them addressed the (cost-)effectiveness of survivorship care in terms of reduced morbidity and/or mortality from late effects. One of these papers was included in Signorelli et al, and two were not included as they concerned HL survivors. We briefly describe these papers below.

4.3.2. Chen et al (2009)
Chen et al (2009) evaluated lipid screening and statin use in a hypothetical cohort of 30-year old 5-year HL survivors treated with chest radiation (13). They compared no screening with screening at 1-, 3-, 5-, or 7-year intervals and used Markov models to calculate life expectancy, quality-adjusted life expectancy and life-time costs. A 3-year interval was most cost-effective. The authors pointed out that their results were very sensitive to the assumptions made in the models regarding the magnitude of the risk increase of CVD in Hodgkin survivors and the efficacy of statin use in this specific population (unknown because of potential different pathogenesis of CVD).

4.3.3. Furzer et al (2020)
Furzer et al (2020) used simulation models to evaluate the cost-utility of 8 different breast cancer screening strategies for adolescent HL survivors treated with chest radiotherapy, starting at 25 years of age (14). No comparison was made with no screening (i.e. no screening till the age of recommended population screening); the reference was annual MRI and mammography from age 25 (Childhood Oncology Group guideline). Among all assessed surveillance strategies, the differences in life expectancy were small, but annual MRI alone from age 25 was the most cost-effective strategy. In a previous simulation model, the same authors estimated that 80 patients would need to be invited to MRI-based screening to prevent one breast cancer death, indicating that MRI-based screening should reduce breast cancer mortality in young Hodgkin survivors (32). A limitation of these simulation analyses is that model assumptions about the efficacy of breast cancer screening at young ages were mostly based on BRCA1/2 mutation carriers, who are known to have different breast cancer tumor characteristics than other women.

4.3.4. Howell et al (2009)
Howell et al (2009) described the results of the National Notification Risk Assessment and Screening Programme launched by the U.K. Department of Health in 2003 (11). Women treated with chest radiotherapy for cancer before age 36 (mostly HL survivors) were recalled by their general practitioner to undergo mammographic breast cancer screening. In the study area (Manchester) 58% of women attended, and breast cancer seemed to be diagnosed in an earlier stage; this was, however, based on only 5 breast cancers (vs. 13 breast cancers diagnosed outside the program) and neither national data of this programme nor follow-up data on incident screening have been published.

4.3.5. Simulation modelling studies
In 2014, two simulation modelling studies evaluated the efficacy and cost-effectiveness of echocardiography followed by angiotensin-converting enzyme (ACE) inhibitor and ß-blocker therapies after asymptomatic left ventricular dysfunction diagnosis in childhood cancer survivors treated with anthracyclines. Wong et al (2014), using Markov models, reported that screening (at 1- to 5-year intervals) versus no screening extended life expectancy by 6 months and QALYs by 1.6 months, and reduced the cumulative incidence of heart failure by 18% at 30 years after cancer diagnosis (33). However, less frequent screenings were more cost-effective and maintained 80% of the health benefits. Results were most sensitive to the assumed magnitude of treatment efficacy; higher treatment efficacy resulted in lower cost-effectiveness. Limitations noted by the authors were that lifetime non-heart failure mortality and the cumulative incidence of heart failure >20 years after diagnosis were extrapolated; the efficacy of ACE inhibitor and ß-blocker therapy in childhood cancer survivors with subclinical heart failure is unknown. In a similar simulation analysis with different assumptions, Yeh et al (2014) estimated a lifetime risk for heart failure among 5-year childhood cancer survivors of 18.8% without routine cardiac assessment (average age at onset, 58.8 years) (34). Routine echocardiography reduced heart failure lifetime risk by 2.3% (with assessment every 10 years) to 8.7% (annual assessment). The authors noted the same limitations as Wong et al (2014), illustrating that there is a strong need for clinical practice studies into the effectiveness of screening methods used in cancer survivorship care.

In conclusion, so far no studies have assessed the (cost-)effectiveness of cancer survivorship care focused on screening and treatment for late adverse effects of treatment.

5. TIME SCHEDULE
The proposed study will take 4.5 years to complete:
- 0.75 year for preparation, development of the study protocol and obtaining medical ethics approval
- 2.75 years of patient inclusion and data collection
- 1.0 year for data-analysis and reporting

6. REQUESTED PERSONNEL
The project coordinator (0.2 FTE for 4.5 years) will coordinate the project, involving communication with all parties involved and coordination of data collection and analysis. This requires excellent coordination skills since many parties and different stakeholders are involved (12 different hospitals with haemato-oncologists, radiation oncologists, nursing specialists and cardiologists, the patient organisation, five disease registries and the survivors' general practitioners). The coordinator will also

obtain Medical Ethics approval in all 12 hospitals and organise research contracts according to GDPR rules.
A PhD student will perform part of the data collection from the medical records (20%), perform quality control with the data abstractor, send letters to general practitioners, perform all linkages with disease registries, and analyse the yield and effectiveness data as well as attendance and adherence data.
A data abstractor (1 FTE for 2.75 year) will perform most of the data collection (abstraction of 80% of the medical records) and assist the PhD student in sending letters to general practitioners.
A junior health technology assessment researcher will perform the (cost-)effectiveness analysis (1 FTE for 0.5 year) under supervision of a senior health technology assessment researcher (0.2 FTE for 0.5 year).

7. FEASIBILITY
The infrastructure of the BETER clinics and the availability of our nationwide retrospective cohort of lymphoma survivors greatly facilitate the quasi-experimental study design. So far, 5 out of 18 currently active BETER clinics have actively recalled HL survivors during more than 5 years. The remaining BETER centres are planning to start in the course of 2020-2022. Our project is strongly supported by the entire BETER Consortium and the Consortium has developed a tradition of successful collaboration in a number of projects such as assessment of late adverse effects of treatment, development of new screening strategies, the BETER-REFLECT Biobank and risk prediction modelling studies (15,16).

8. REACTION TO THE COMMENTS OF THE COMMITTEE
We thank the committee for their valuable feedback

8.1. De commissie merkt op dat de subhypotheses bijdragen aan het concretiseren van de hypothese, maar mogelijk niet toetsbaar zijn met de in de aanvraag genoemde steekproefgrootte. Daarnaast vraagt de commissie zich af waarom het evalueren van het effect op kwaliteit van leven en kosten als secundaire doelstelling is geformuleerd. Door de commissie wordt verondersteld dat de evaluatie van kosten en effect op kwaliteit van leven onderdeel zijn van de (kosten)effectiviteitsanalyse.

We agree with the committee that quality of life is a crucial outcome in this study and we have therefore made it one of the primary outcomes. Power to detect relevant differences in QoL is always much higher than for disease outcomes and therefore the proposed project is currently powered to detect a small difference (effect size = 0.20 standard deviation) in the overall accumulated DALYs between the two groups. This sample size also allows for the detection of medium effect-sizes of 0.50 standard deviation in sub-analyses (see 1.7.3. Effect size for other analyses). Other more explorative analyses concerning secondary outcomes, such as the assessment of the association between attendance to the BETER clinic and knowledge of late effects and distress about late effects, may prove to be underpowered.

8.2. Patient (P):
8.2.1. U dient de inclusie- en exclusiecriteria in de subsidieaanvraag duidelijker te beschrijven.

We have further elaborated on the inclusion/exclusion criteria in paragraph ‘1.2. PATIENT (P)’.

8.2.2. De commissie vindt onduidelijk hoe u gegevens kunt verzamelen over jongere overlevers van lymphoma, zoals beoogd in uw project, wanneer gebruik zal worden gemaakt van retrospectieve data. U dient dit nader toe te lichten.

For the conduct of the study, we use a previously established comprehensive data set from a large nationwide retrospective cohort of all lymphoma survivors treated in 27 BETER centres between 1965 and 2012. We have now explained this extensively in the proposal.

8.3. Outcome (O): de commissie vindt de dataverzameling in uw aanvraag onduidelijk beschreven en verzoekt u:
8.3.1. Nader toe te lichten op welke momenten informatie over kwaliteit van leven en productiviteitsverliezen wordt verzameld. De commissie merkt op dat op basis van het projectidee lijkt dat dit eenmalig wordt gemeten en de commissie vraagt zich af wat de toegevoegde waarde dan is van deze metingen.

As we describe in paragraph ‘1.1.1. Considerations about other study designs’ a pre-test post-test control group design was not possible: baseline measurements in centres not yet participating could not be performed, as treating physicians considered it unethical to invite survivors who were discharged many years ago back to the hospital for a baseline assessment only, without subsequently offering them survivorship care according to the BETER guidelines. In all survivors health-related quality of life (HRQoL) and health-related productivity losses (HRPL) will be assessed once, at end of follow-up in 2021-2024. We describe in the proposal that the matching of the intervention and control groups allows for valid comparisons.

8.3.2. Toe te lichten hoe de DALYs worden geoperationaliseerd.

We have elaborated on the measurement and calculation of the DALYs in paragraph ‘1.6.1.2. Assessment of Burden of Disease’.

8.4. Follow-up time (T): omdat de twee groepen van 300 personen gezonde personen zijn, vraagt de commissie zich af of de follow-up periode van 4 tot 9 jaar lang genoeg is om de effecten van de screening te evalueren. Zij verzoekt u dit nader te onderbouwen in de subsidieaanvraag.

HL survivors visiting the BETER clinics may not have symptoms, but their risks of serious late adverse treatment effects are substantial. The 40-year cumulative incidence of coronary heart disease, valvular heart disease, cardiomyopathy and congestive heart failure is 50%, at a median age of 65 years (16), the 30-year cumulative incidence of breast cancer is 17-26%, at a median age of 55 years (15). We recently showed that, at age 65, HL survivors had a 68% cumulative risk of having

developed either CVD or second malignancy (4). Moreover, these conditions occur at younger ages than in the general population. Furthermore, the absolute risk of hypothyroidism is 44-67% 25 years after treatment (3,35) and the relative risk of overwhelming post-splenectomy infections is elevated up to 20 (17). To assess the potential health gains (in terms of averted DALYs) of BETER survivorship care, a model was constructed in which the burden of disease accumulated during 5-year follow-up was compared between the intervention group (screened at a BETER clinic) and the control group. The model includes cumulative incidence of the late adverse events after treatment under study: (risk factors of) CVD, breast cancer, hypothyroidism and overwhelming post-splenectomy infections due functional asplenia. We powered our analyses on the estimated average number of DALYs accumulated per survivor in 5 year follow-up in the intervention a control group. As the follow-up in the proposed study is expected to be longer (on average 8 years), we are confident that the follow-up will be long enough to evaluate the effects of screening.

8.5. Study design (s):
8.5.1. De commissie vraagt zich af of u sterfte kunt meten, omdat het matching protocol alleen patiënten kan selecteren die zich aanmelden én dus nog in leven zijn. De commissie vraagt u dit punt te verduidelijken.

For the conduct of the study, we use a previously established comprehensive data set from a large nationwide retrospective cohort of all lymphoma survivors treated in 27 BETER centres between 1965 and 2012, with patient characteristics, treatment data, and outcome including date and cause of death. This allows selection of a control population of individuals who were not yet screened during 2013-2016, yet alive at the time the corresponding survivor in the intervention group was invited to come to the BETER clinic. Matched controls eligible for BETER screening in 2013-2016 who died during follow-up, prior to invitation for BETER screening in a BETER clinic 2021-2024, are eligible for our study. Date and cause of death are also known for survivors who were invited to attend a BETER clinic (regardless of whether they attended or not). This way mortality rates can be assesses for both the intervention and the control group.

8.5.2. De commissie merkt op dat uw aanvraag onvoldoende informatie bevat over de uit te voeren analyses van klinische effecten en kosteneffectiviteitsanalyse. U dient de geplande analyses nader toe te lichten in uw subsidieaanvraag.

We have elaborated on the subject in paragraph ‘2. COST EFFECTIVENESS ANALYSIS (CEA)’.

8.5.3. U dient nader toe te lichten hoe de matching plaatsvindt.
We have elaborated on this subject in paragraph ‘1.2.3. Control group’

8.5.4. Voor de kosteneffectiviteitsanalyse wordt gebruik gemaakt van een DES model, maar het is de commissie niet duidelijk waar de data voor dit model vandaan komen. De commissie verzoekt u dit nader toe te lichten.

We have further elaborated on this subject in paragraph ‘2.5. Cost-effectiveness Analysis’. The cost-effectiveness analysis will be performed by means of a discrete event simulation, to take the factor “timing” into account and because patient level data is available. We will perform an analysis on the 5-year data, based on the study. In addition, we will estimate the long term effects by means of a discrete event simulation, based on the study data, combined with extrapolations with data from e.g. literature.

8.5.5. U wordt verzocht om in de subsidieaanvraag de sample size berekening nader uit te werken. U dient hierbij in ieder geval in te gaan op hoe vaak gezondheidseffecten worden verwacht, wat de incidentie is en bij welke leeftijd.

We have elaborated on this subject in paragraphs ‘1.7.1. Assessing the potential health gains’ and ‘1.7.2. Sample size calculation’.

8.5.6. Daarnaast vraagt de commissie zich af of het voorgestelde aantal patiënten groot genoeg is om ook uitspraken te doen over verschillen in de frequentie van follow-up. Dit wordt niet nader toegelicht in het projectidee. Zij verzoekt u dit punt nader toe te lichten in de subsidieaanvraag.

The committee is right to question whether the sample size and variation in screening in the study population are large enough to assess the optimal frequency of certain screens. These analyses are of more explorative nature and may not provide significant results. However, the data generated in the proposed study will also be used in the cost-effectiveness analysis, incorporating several scenarios for follow up strategies to give us more insight in an optimal frequency.

8.6 Een deel van het projectbudget wordt gebruikt voor de start van vijf nieuwe BETER klinieken. De commissie acht het opzetten van vijf nieuwe BETER klinieken binnen het huidige project onnodig. U wordt verzocht dit onderdeel niet in de subsidieaanvraag op te nemen.

We are sorry for this misunderstanding: the start-up costs of the new BETER clinics were never meant to be part of this project proposal’s budget. The starting of BETER clinics is funded by the BETER Consortium and the participating centre. What we meant to say is that during this phase of the project, the centres were setting up the new clinics.

9. HISTORY GRANT APPLICATION
Feedback on a previous project idea from our group (DoelmatigheidsOnderzoek 2019-2021 call - September 2018; project number 74546) showed that the ZonMw review committee felt that the project did not fit the call for cost-effectiveness research proposals at that time. We fear that this decision may have been based on the misconception that the effectiveness of the BETER survivorship care did not need substantiating, as it is based on so-called evidence-based guidelines. The evidence underpinning these guidelines mainly consists of evidence for the increased risks of late adverse treatment effects in lymphoma survivors. The screening recommendations, however, had to be based on evidence from other high risk populations, which is

debatable as the pathogenesis of treatment-related adverse events may be different. Consequently, (cost-)effectiveness of BETER follow-up care has never been demonstrated. We adapted the previous proposal into a comprehensive evaluation of the follow-up care at the BETER clinics and strongly feel that the new proposal is a good fit for the current call.

Expertise, voorgaande activiteiten en producten / Expertise, prior activities and products

BETER CONSORTIUM
This project builds on the nationwide collaboration of the BETER consortium (Better care after (non-)Hodgkin lymphoma, Evaluation of long-term Treatment Effects and screening Recommendations), in which currently 33 lymphoma treatment centres participate, including all University Medical Centres, NKI and all radiotherapy facilities. The consortium consists of radiation oncologists, heamato-oncologists, epidemiologists, psychologists, nurse practitioners, and representatives from the Dutch Society of General Practitioners and the Haematology Patient Federation Hematon. Through the multidisciplinary composition and broad experience of the various experts in the consortium, the consortium has been very effective in organizing an infrastructure for survivorship care for lymphoma survivors (KWF/Alpe d'HuZes grant, NKI 2011-5270, with principal investigators Prof. Dr. F.E. van Leeuwen and Dr. B.M.P. Aleman, coordinator Dr A. Nijdam). The consortium has broad experience with the development of screening guidelines (richtlijnendatabase.nl) and a website for survivors and physicians (www.beternahodgkin.nl). The existing BETER infrastructure is already effectively used for research on late effects of lymphoma treatment and new screening methods, led by F.E. v Leeuwen and B.M.P. Aleman (see Supplement 5 Competence of the project leader)

BETER HEALTH CARE PROVIDERS
Both Dr. B.M.P. Aleman (Radiation Oncology) and Prof. Dr. J. Zijlstra (Haemato-oncology Amsterdam UMC) run a weekly BETER outpatient clinic and have extensive experience in clinical practice in recalling eligible lymphoma survivors for surveillance and screening them according to the BETER guidelines.

CARDIO-ONCOLOGY
Prof. A. Maas, Cardiology RadboudUMC, has extensive experience in screening for cardiovascular diseases and and cancer survivorship care.

BURDEN OF DISEASE FROM CHRONIC DISORDERS
Prof. Dr. M. Verschuren is Head of the Department Chronic Diseases Determinants, Prevention and Health Services RIVM (National Institute for Public Health and the Environment) and Professor of Healthy Vascular Ageing in Public Health Perspective at Utrecht University; she has extensive experience in the assessment of burden of disease from chronic diseases.

HEALTH TECHNOLOGY ASSESSMENT
Dr. V. Retèl (PhD degree in Health Economics) is Head of the Health Technology Assessment Facility of NKI and also has a part-time staff position at the University of Twente (Department of Health Technology and Services Research). She has extensive experience in cost-effectiveness research of new cancer treatments and diagnostics.

PATIENT REPRESENTATIVE
Cecile van Dierendonck has been a patient advocate for BETER since its establishment in 2009. Since the 1990s she has actively participated in various advisory groups, such as the board of the Dutch Federation of Cancer Patients (NFK) and the Dutch Haematology Patient Federation Hematon. Within Hematon Cecile van Dierendonck is a member of the HOPP ('Hematon Onderzoek Patiënten Participatie') committee; in this capacity she assesses proposals for scientific studies on their merits from a patient perspective.

Publicaties / Publications

Cancer survivorship care, late effects of cancer treatment

1. Aleman BMP, Zijlstra JM, Nijdam A, van 't Veer MB, Raemaekers JMM, Leeuwen FE namens de leden van het BETER-consortium. Geïndividualiseerde nazorg voor overlevers van (non)hodgkinlymfoom: achtergrond en ervaringen van de BETER polikliniek. Geaccepteerd voor publicatie in Ned Tijdschr Hematologie.
2. Jacobse JN, Steggink LC, Sonke GS, Schaapveld M, Hummel YM, Steenbruggen TG, Lefrandt JD, Nuver J, Crijns APG, Aleman BMP, van der Meer P, Gietema JA, van Leeuwen FE. Myocardial dysfunction in long-term breast cancer survivors treated at ages 40-50 years. Eur J Heart Failure. 2020;22(2):338.
3. Rigter LS, Spaander MCW, Aleman BMP, Bisseling TM, Moons LM, Cats A, Lugtenburg PJ, Janus CPM, Petersen EJ, Roesink JM, van der Maazen RWM, Snaebjornsson P, Kuipers EJ, Bruno MJ, Dekker E, Meijer GA, de Boer JP, van Leeuwen FE, van Leerdam ME. High prevalence of advanced colorectal neoplasia and serrated polyposis syndrome in Hodgkin lymphoma survivors. Cancer 2019;125(6):990.
4. Nijdam A, Dekker N, Aleman BMP, van 't Veer MB, Daniels LA, van der Maazen RW, Janus CPM, de Weijer RJ, Zijlstra JM, Stedema FG, Ta BD, Posthuma EFM, Manenschijn A, Dielwart MFH, Bilgin YM, van den Heuvel MJ, Boersma RS, van Leeuwen FE, Raemaekers JMM. Setting up a national infrastructure for survivorship care after treatment for Hodgkin lymphoma. Br J Haematology 2019;186(4):e103.
5. Starreveld DEJ, Daniels LA, Valdimarsdottir HB, Redd WH, de Geus JL, Ancoli-Israel S, Lutgendorf S, Korse CM, Kieffer JM, van Leeuwen FE, Bleiker EMA. Light therapy as a treatment of cancer-related fatigue in (non-)Hodgkin lymphoma survivors (SPARKLE trial): study protocol of a multicenter randomized controlled trial. BMC cancer 2018;18(1):880.
6. de Vries S, Schaapveld M, van Nimwegen FA, Jozwiak K, Lugtenburg PJ, Daniels LA, Roesink JM, van der Maazen RWM, Kok WEM, Aleman BMP, van Leeuwen FE. High burden of subsequent malignant neoplasms and cardiovascular disease in long-term Hodgkin lymphoma survivors. Br J Cancer 2018;118(6):887.
7. Aleman BM, Kok WE, van 't Veer MB, FE vL. Richtlijn: Screening op cardiovasculaire schade na hodgkinlymfoom; een

van de richtlijnen voor nazorg van vijfjaarsoverlevenden van hodgkinlymfoom. Ned Tijdschr Oncologie 2018;15:101-5. 2018.
8. van Nimwegen FA, Ntentas G, Darby SC, Schaapveld M, Hauptmann M, Lugtenburg PJ, Janus CPM, Daniels L, van Leeuwen FE, Cutter DJ, Aleman BMP. Risk of heart failure in survivors of Hodgkin lymphoma: effects of cardiac exposure to radiation and anthracyclines. Blood 2017;129(16):2257.
9. Krul IM, Opstal-van Winden AWJ, Aleman BMP, Janus CPM, van Eggermond AM, De Bruin ML, Hauptmann M, Krol ADG, Schaapveld M, Broeks A, Kooijman KR, Fase S, Lybeert ML, Zijlstra JM, van der Maazen RWM, Kesminiene A, Diallo I, de Vathaire F, Russell NS, van Leeuwen FE. Breast Cancer Risk After Radiation Therapy for Hodgkin Lymphoma: Influence of Gonadal Hormone Exposure. Int J Rad Oncol Biol Phys 2017;99(4):843.
10. Hoedjes M, van Stralen MM, Joe STA, Rookus M, van Leeuwen F, Michie S, Seidell JC, Kampman E. Toward the optimal strategy for sustained weight loss in overweight cancer survivors: a systematic review of the literature. J Cancer Survivorship: Research and Practice 2017;11(3):360.
11. van Nimwegen FA, Schaapveld M, Cutter DJ, Janus CP, Krol AD, Hauptmann M, Kooijman K, Roesink J, van der Maazen R, Darby SC, Aleman BM, van Leeuwen FE. Radiation Dose-Response Relationship for Risk of Coronary Heart Disease in Survivors of Hodgkin Lymphoma. J Clin Oncol 2016;34(3):235.
12. Ng AK, van Leeuwen FE. Hodgkin lymphoma: Late effects of treatment and guidelines for surveillance. Sem Hematol 2016;53(3):209.
13. Jacobsen PB, Rowland JH, Paskett ED, Van Leeuwen FE, Moskowitz C, Katta S, Wollins D, Robison LL. Identification of Key Gaps in Cancer Survivorship Research: Findings From the American Society of Clinical Oncology Survey. J Oncol Practice 2016;12(3):190.
14. van Nimwegen FA, Schaapveld M, Janus CM, Krol AD, Petersen EJ, Raemaekers JM, Kok WE, Aleman BM, van Leeuwen FE. Cardiovascular disease after Hodgkin lymphoma treatment: 40-year disease risk. JAMA Int Med. 2015;175(6):1007.
15. Schaapveld M, Aleman BMP, van Eggermond AM, Janus CPM, Krol ADG, van der Maazen RWM, Roesink J, Raemaekers JMM, de Boer JP, Zijlstra JM, van Imhoff GW, Petersen EJ, Poortmans PMP, Beijert M, Lybeert ML, Mulder I, Visser O, Louwman MWJ, Krul IM, Lugtenburg PJ, van Leeuwen FE. Second Cancer Risk Up to 40 Years after Treatment for Hodgkin's Lymphoma. N Engl J Med. 2015;373(26):2499.
16. Dekker N, van 't Veer MB, Aleman BM, van Leeuwen FE, Raemaekers JM. [The BETER survivorship care initiative for Hodgkin lymphoma; tailored survivorship care for late effects of treatment]. Ned Tijdschr Geneesk 2015;159:A9269.
17. Van Nimwegen FA, Schaapveld M, Janus CPM, Krol ADG, Raemaekers JMM, Kremer LCM, Stoval M, Aleman BMP, Van Leeuwen FE. Risk of diabetes mellitus in long-term survivors of Hodgkin lymfoma. J Clin Oncol 2014;32(29):3257.
18. Mulder RL, Kremer LCM, Hudson MM, Bhatia S, Landier W, Levitt G, Constine LS, Wallace WH, van Leeuwen FE, Ronckers CM, Henderson TO, Dwyer M, Skinner R, Oeffinger KC. Recommendations for breast cancer surveillance for female survivors of childhood, adolescent, and young adult cancer given chest radiation: a report from the International Late Effects of Childhood Cancer Guideline Harmonization Group. Lancet Oncol. 2013;14(13):e621.
19. Sieswerda E, Postma A, van Dalen EC, van der Pal HJ, Tissing WJ, Rammeloo LA, Kok WE, van Leeuwen FE, Caron HN, Kremer LC. The Dutch Childhood Oncology Group guideline for follow-up of asymptomatic cardiac dysfunction in childhood cancer survivors. Annals of Oncology 2012;23(8):2191.
20. Oeffinger KC, van Leeuwen FE, Hodgson DC. Methods to assess adverse health-related outcomes in cancer survivors. Cancer Epid Bio Prev. 2011;20(10):2022.
21. Geenen MM, Cardous-Ubbink MC, Kremer LC, van den Bos C, van der Pal HJ, Heinen RC, Jaspers MW, Koning CC, Oldenburger F, Langeveld NE, Hart AA, Bakker PJ, Caron HN, van Leeuwen FE. Medical assessment of adverse health outcomes in long-term survivors of childhood cancer. JAMA. 2007;297(24):2705.

Cost-effectiveness of cancer treatment/screening

1. Koole SN, van Lieshout C, van Driel WJ, van Schagen E, Sikorska K, Kieffer JM, Schagen van Leeuwen JH, Schreuder HWR, Hermans RH, de Hingh IH, van der Velden J, Arts HJ, Massuger L, Aalbers AG, Verwaal VJ, Van de Vijver KK, Aaronson NK, van Tinteren H, Sonke GS, van Harten WH, Retel VP. Cost Effectiveness of Interval Cytoreductive Surgery With Hyperthermic Intraperitoneal Chemotherapy in Stage III Ovarian Cancer on the Basis of a Randomized Phase III Trial. Journal of Clinical Oncology 2019;37(23):2041-2050.
2. Verbeek JGE, Atema V, Mewes JC, van Leeuwen M, Oldenburg HSA, van Beurden M, Hunter MS, van Harten WH, Aaronson NK, Retel VP. Cost-utility, cost-effectiveness, and budget impact of Internet-based cognitive behavioral therapy for breast cancer survivors with treatment-induced menopausal symptoms. Breast cancer research and treatment. 2019;178(3):573-585.
3. Retel VP, Steuten LMG, Geukes Foppen MH, Mewes JC, Lindenberg MA, Haanen J, van Harten WH. Early cost-effectiveness of tumor infiltrating lymphocytes (TIL) for second line treatment in advanced melanoma: a model-based economic evaluation. BMC cancer. 2018;18(1):895.

Referenties / References

1. Netherlands Comprehensive Cancer Organisation (IKNL). Dutch Cancer figures 1961-2017. Netherlands Cancer Registry. 2020.
2. Borchmann P, Eichenauer DA, Engert A. State of the art in the treatment of Hodgkin lymphoma. Nat Rev Clin Oncol. 2012;9(8):450–9.
3. Ng AK, Van Leeuwen FE. Hodgkin lymphoma: Late effects of treatment and guidelines for surveillance. Semin Hematol. 2016;53(3):209–15.
4. De Vries S, Schaapveld M, Van Nimwegen FA, Józwiak K, Lugtenburg PJ, Daniëls LA, et al. High burden of subsequent malignant neoplasms and cardiovascular disease in longterm Hodgkin lymphoma survivors. Br J Cancer. 2018;118(6):887–95.
5. Barbui T, Björkholm M, Gratwohl A. Cancer survivorship programs: Time for concerted action. Haematologica. 2014;99(8):1273–6.

6. Armenian SH, Hudson MM, Mulder RL, Chen MH, Constine LS, Dwyer M, et al. Recommendations for cardiomyopathy surveillance for survivors of childhood cancer: A report from the International Late Effects of Childhood Cancer Guideline Harmonization Group. Lancet Oncol. 2015;16(3):e123–36.
7. Knowledge Institute of Medical Specialists. The Dutch Medical Guideline Database for Secondary Care. Search for “na Hodgkinlymfoom.” Available from: richtlijnendatabase.nl
8. Nijdam A, Dekker N, Aleman BMP, van ’t Veer MB, Daniels LA, Maazen RW, et al. Setting up a national infrastructure for survivorship care after treatment for Hodgkin lymphoma. Br J Haematol. 2019;186(4):e103–8.
9. Griebsch I, Brown J, Boggis C, Dixon A, Dixon M, Easton D, et al. Cost-effectiveness of screening with contrast enhanced magnetic resonance imaging vs X-ray mammography of women at a high familial risk of breast cancer. Br J Cancer. 2006;95(7):801–10.
10. Silber JH, Cnaan A, Clark BJ, Paridon SM, Chin AJ, Rychik J, et al. Enalapril to prevent cardiac function decline in long-term survivors of pediatric cancer exposed to anthracyclines. J Clin Oncol. 2004;22(5):820–8.
11. Howell SJ, Searle C, Goode V, Gardener T, Linton K, Cowan RA, et al. The UK national breast cancer screening programme for survivors of Hodgkin lymphoma detects breast cancer at an early stage. Br J Cancer. 2009;101(4):582–8.
12. Daniëls LA, Krol ADG, De Graaf MA, Scholte AJHA, Van’t Veer MB, Putter H, et al. Screening for coronary artery disease after mediastinal irradiation in Hodgkin lymphoma survivors: Phase II study of indication and acceptance. Ann Oncol. 2014;25(6):1198–203.
13. Chen AB, Punglia RS, Kuntz KM, Mauch PM, Ng AK. Cost effectiveness and screening interval of lipid screening in Hodgkin’s lymphoma survivors. J Clin Oncol. 2009;27(32):5383–9.
14. Furzer J, Tessier L, Hodgson D, Cotton C, Nathan PC, Gupta S, et al. Cost-Utility of Early Breast Cancer Surveillance in Survivors of Thoracic Radiation-Treated Adolescent Hodgkin Lymphoma. J Natl Cancer Inst. 2020;112(1):63–70.
15. Schaapveld M, Aleman BMP, Van Eggermond AM, Janus CPM, Krol ADG, van der Maazen RWM, et al. Second Cancer Risk Up to 40 Years after Treatment for Hodgkin’s Lymphoma. N Engl J Med. 2015;373(26):2499–511.
16. Van Nimwegen FA, Schaapveld M, Janus CPM, Krol ADG, Petersen EJ, Raemaekers JMM, et al. Cardiovascular disease after Hodgkin lymphoma treatment: 40-year disease risk. JAMA Intern Med. 2015;175(6):1007–17.
17. Foss Abrahamsen A, Høiby EA, Hannisdal E, Jørgensen OG, Holte H, Hasseltvedt V, et al. Systemic pneumococcal disease after staging splenectomy for Hodgkin’s disease 1969-1980 without pneumococcal vaccine protection: a follow-up study 1994. Eur J Haematol. 1997;58(2):73–7.
18. Stouthard MEA, Essink-Bot M, Bonsel G, Barendregt J, Kramers P, Van de Water H, et al. Disability weights for diseases in the Netherlands. Tijdschr Gerontol Geriatr. 1997;(January 1997):1–67.
19. Hilderink HBM, Plasmans MHD, Snijders BEP, Boshuizen HC, René Poos MJJC, van Gool CH. Accounting for multimorbidity can affect the estimation of the Burden of Disease: A comparison of approaches. Arch Public Heal. 2016;74(1).
20. Vondeling G, Rozenbaum M, Dvortsin E, Postma M, Zeevat F. Burden of Early and Advanced Breast Cancer in The Netherlands. Value Heal. 2016;19(7):A756.
21. Salomon JA, Haagsma JA, Davis A, de Noordhout CM, Polinder S, Havelaar AH, et al. Disability weights for the Global Burden of Disease 2013 study. Lancet Glob Heal. 2015;3(11):e712–23.
22. Ware JE, Sherbourne CD. The MOS 36-item short-form health survey (SF-36). I. Conceptual framework and item selection. Med Care. 1992 Jun [cited 2014 Jul 17];30(6):473–83.
23. Janssen MF, Pickard AS, Golicki D, Gudex C, Niewada M, Scalone L, et al. Measurement properties of the EQ-5D-5L compared to the EQ-5D-3L across eight patient groups: a multi-country study. Qual Life Res. 2013 Sep;22(7):1717–27.
24. Bouwmans C, Krol M, Severens H, Koopmanschap M, Brouwer W, Hakkaart-van Roijen L. The iMTA Productivity Cost Questionnaire: A Standardized Instrument for Measuring and Valuing Health-Related Productivity Losses. Value Health. 2015 Sep;18(6):753–8.
25. Oeffinger KC, Ford JS, Moskowitz CS, Diller LR, Hudson MM, Chou JF, et al. Breast Cancer Surveillance Practices Among Women Previously Treated With Chest Radiation for a Childhood Cancer. JAMA Intern Med. 2009 Jan 28;301(4):404–14.
26. Oeffinger KC, Hudson MM, Mertens AC, Smith SM, Mitby PA, Eshelman-Kent DA, et al. Increasing rates of breast cancer and cardiac surveillance among high-risk survivors of childhood Hodgkin lymphoma following a mailed, one-page survivorship care plan. Pediatr Blood Cancer. 2011 May;56(5):818–24.
27. Custers JAE, Kwakkenbos L, van de Wal M, Prins JB, Thewes B. Re-validation and screening capacity of the 6-item version of the Cancer Worry Scale. Psychooncology. 2018;27(11):2609–15.
28. Groot MT, Baltussen R, Uyl-De Groot CA, Anderson BO, Hortobágyi GN. Costs and health effects of breast cancer interventions in epidemiologically different regions of Africa, North America, and Asia. Breast J. 2006;12(SUPPL. 1).
29. Signorelli C, Wakefield CE, Fardell JE, Wallace WHB, Robertson EG, McLoone JK, et al. The impact of long-term follow-up care for childhood cancer survivors: A systematic review. Crit Rev Oncol Hematol. 2017;114:131–8.
30. Ford JS, Chou JF, Sklar CA. Attendance at a survivorship clinic: impact on knowledge and psychosocial adjustment. J Cancer Surviv. 2013 Dec 22;7(4):535–43.
31. Blaauwbroek R, Groenier KH, Kamps WA, Meyboom-de Jong B, Postma A. Late effects in adult survivors of childhood cancer: the need for life-long follow-up. Ann Oncol Off J Eur Soc Med Oncol. 2007 Nov;18(11):1898–902.
32. Hodgson DC, Cotton C, Crystal P, Nathan PC. Impact of Early Breast Cancer Screening on Mortality Among Young Survivors of Childhood Hodgkin’s Lymphoma. J Natl Cancer Inst. 2016 Jul;108(7).
33. Wong FL, Bhatia S, Landier W, Francisco L, Leisenring W, Hudson MM, et al. Cost-effectiveness of the children’s oncology group long-term follow-up screening guidelines for childhood cancer survivors at risk for treatment-related heart failure. Ann Intern Med. 2014 May 20;160(10):672–83.
34. Yeh JM, Nohria A, Diller L. Routine echocardiography screening for asymptomatic left ventricular dysfunction in childhood cancer survivors: a model-based estimation of the clinical and economic effects. Ann Intern Med. 2014 May 20;160(10):661–71.
35. Hancock SL, Cox RS, McDougall IR. Thyroid diseases after treatment of Hodgkin’s disease. N Engl J Med. 1991 Aug 29;325(9):599–605.

Disciplines / Disciplines

- Kanker / Cancer
- Bloed- en lymfeziekten / Haematology

Financiële gegevens / Financial data

ZonMw budget

	Jaar / Year								
**Kostenpost**	*1*	*2*	*3*	*4*	*5*	*6*	*7*	*8*	**Totaal / Total**
Personeel	0	0	0	0	587.362	0	0	0	587.362
Materieel	0	0	0	0	0	0	0	0	0
Implementatie	0	0	0	0	0	0	0	0	0
Apparatuur	0	0	0	0	0	0	0	0	0
Overig	3.750	3.750	13.750	11.375	5.500	0	0	0	38.125
**Totaal / Total**	**3.750**	**3.750**	**13.750**	**11.375**	**592.862**	**0**	**0**	**0**	**625.487**

Co-financiering / Cofinancing

Naam co-financier / Name of cofinancier	Bedrag / Amount	Status

Bijzondere gegevens / Additional information

Vergunningen / Permits

	Verklaring nodig / Statement required?		Status verklaring / Statement status		
	Ja / Yes	Nee / No	Verkregen / Acquired	Aangevraagd / Applied	Nog niet aangevraagd / Not applied yet
METC	X				X
DEC		X			
WBO		X			

Onderschrijvingen / Assents

	Ja / Yes	Nee / No	N.v.t. / N.A.
Code biosecurity / Code Biosecurity			X
Code openheid dierproeven / Code Transparency of Animal Testing			X

Andere vergunningen / Other permits

Historie subsidieaanvraag / History grant application

Deze aanvraag is eerder ingediend bij het programma / This grant application has previously been submitted to the ZonMw programme:

DoelmatigheidsOnderzoek 2019-2021

Projectnummer / Project number:

74546

Deze aanvraag is ook ingediend bij organisatie / This grant application has also been submitted to organization:

Specifieke financieringsvoorwaarden voor de oproep evaluatieonderzoek ZE&GG – extra ronde 2019

Welk bedrag kan aangevraagd worden
In totaal is in deze subsidieronde een bedrag van € 6,6 miljoen beschikbaar. In deze ronde worden geen beperkingen gesteld aan de omvang van de gevraagde subsidie en de looptijd van het onderzoek. Wel is het van belang dat zowel het budget als de looptijd realistisch en goed onderbouwd zijn.

Cofinanciering is toegestaan, maar niet verplicht. Indien er sprake is van cofinanciering dient er een letter of commitment toegevoegd te worden bij de uitgewerkte subsidieaanvraag.

Wie kan aanvragen
- Een projectidee kan alleen door een zorg- of onderzoeksinstelling worden ingediend.
- Samenwerking binnen het projectteam met onderzoeksinstellingen, zorginstellingen en zelfstandige zorgprofessionals wordt aangemoedigd. Deze partijen kunnen als mede-aanvrager in de subsidieaanvraag worden opgenomen.
- Zorgverzekeraars, leveranciers en fabrikanten kunnen cofinancier van het project zijn.

Algemene toelichting bij begroting en specifieke financieringsvoorwaarden voor de oproep "Evaluatieonderzoek ZE&GG - extra ronde 2019"

Richtlijnen voor het opzetten van de begroting:
In de begroting worden de volgende posten onderscheiden:
1. Personele kosten
2. Materiële kosten
3. Implementatiekosten
4. Apparatuurkosten
5. Overige kosten

Onderstaand wordt op elk van deze posten nader ingegaan.

Personele kosten
Er is een onderscheid tussen organisaties die verplicht zijn de salaristabellen te hanteren (VSNU leden / NFU leden) zoals overeengekomen in bekostigingsbesluit wetenschappelijke instellingen en organisaties die volgens een uitgebreidere opgaaf te werk moeten gaan.

Leden van de VSNU/NFU
De tabelbedragen zoals gepubliceerd op de website van ZonMw zijn verplicht om te hanteren. Gebruik het bedrag wat overeenkomt met de beoogde looptijd van de inzet voor de betreffende functie. Deze waarde mag als 1 totaal bedrag per lijn item worden opgegeven, of over de jaren verdeeld, zodat het subtotaal het juist bedrag van de tabel weergeeft.
De daadwerkelijk beoogde FTE inzet over de looptijd dient te worden opgegeven.
De overhead opslag wordt op nihil gesteld.

Overige instellingen
Tot de personele kosten worden gerekend:
• de feitelijke salariskosten per jaar van de direct bij de projectuitvoering betrokken personeelsleden; per functie de salarisschaal, het inschalingsniveau en de werktijdfactor aangeven, en 12 maal het bruto maandsalaris berekenen;
• een opslagpercentage op de salariskosten ter dekking van de bijkomende personele kosten van max. 40%. In het opslagpercentage zijn verdisconteerd: sociale lasten, eindejaarsuitkering, 13de maand , vakantiegeld, wachtgeld, ziekterisico, advertentiekosten en overige wervingskosten, reiskosten woon-werkverkeer, ouderschapsverlof en toeslagen, kosten overig verlof, opleidingskosten, ondersteuning personeelszaken, gratificaties, binnenlandse dienstreizen, uitkering bij overlijden, sociale activiteiten, verhuis- en installatiekosten, tegemoetkoming ziektekosten en zogenaamde einde-projectkosten.

Voor alle functies geldt dat, indien het inschalingsniveau van de betrokken functionaris nog niet bekend is, de salariskosten worden berekend volgens het middenpunt van de schaal. Als de inschaling wel bekend is, wordt uitgegaan van de reële inschaling.

In de projectbegrotingen dient te worden uitgegaan van een jaarlijkse stijging van de salarislasten met

maximaal één periodiek en met een correctie ingevolge de inflatie van 2% per jaar. Bij de afrekening zal rekening worden gehouden met de werkelijk gemaakte kosten, met inachtneming van het maximaal ter beschikking gestelde subsidiebedrag.

Ter dekking van de overheadkosten kan bij programma's waar dat is aangegeven, een opslag-percentage op de bruto salariskosten (12 maal bruto maandsalaris plus max. 40%) worden berekend. Het opslagpercentage wordt ter vaststelling aan ZonMw voorgelegd en is geldig voor alle door de subsidieaanvrager in te dienen projecten.

Als gevolg van de algemene overheadopslag vervallen vergoedingen voor de reguliere infrastructuur zoals: begeleiding, indirect personeel, algemene diensten, ethische toetsing, verzekeringen, huisvestingslasten, kantoormaterialen, fotografie en reproductiekosten, porti- en telefoonkosten, automatiserings- en overige ICT-kosten, voorbereiding congresbezoek, representatiekosten en kosten page-charge/reprint/beoordelingskosten voor plaatsing van artikelen.

Materiële kosten

De specifiek voor het project benodigde verbruiksartikelen dienen inclusief BTW in de begroting te worden weergegeven. In het geval voor het project inherente excessieve reiskosten moeten worden gemaakt, kunnen deze onder de post materiële kosten worden opgevoerd.

Apparatuurkosten

Investeringen in apparatuur dienen goed gemotiveerd te worden. Alleen kosten die specifiek voor het project worden gemaakt, zijn subsidiabel; interestkosten worden niet vergoed. De kosten van investeringen (afschrijvingen) kunnen in de projectkosten worden opgenomen naar rato van het gebruik hiervan. Indien van apparatuur de volledige aanschafwaarde wordt gefinancierd, dient rekening gehouden te worden met de restwaarde van deze apparatuur.
Voor afschrijvingen en het bepalen van de restwaarde wordt uitgegaan van de volgende percentages:

* Computerapparatuur:
1e jaar - 40%;
2e jaar - 30%;
3e jaar - 20%;
4e jaar - 10%.

* Overige apparatuur: lineaire afschrijving in 5 jaar (20% per jaar).

Implementatiekosten

Onder implementatiekosten worden de kosten verstaan die worden gemaakt in het kader van het verspreiden en overdragen van kennis/ervaringen uit het project. Ook kosten in het kader van het voorbereiden van de daadwerkelijke invoering van de projectresultaten vallen hieronder. Voorbeelden van kostenposten zijn: publicaties, nieuwsbrieven, foldermateriaal, mailingen, lezingen, expertmeetings, uitwisselingsbijeenkomsten en de organisatie van congressen.

Overige kosten

Tot de overige kosten worden gerekend: kosten zoals het uitbesteden van werkzaamheden, juridische advisering, eventuele marketingadviezen, media adviezen en mediakosten zoals publiciteitscampagnes.

Budgetformat	Evaluatieonderzoek ZE&GG - extra ronde 2019
Datum	
Project naam	ationwide long-term follow-up care for lymphoma survivors in t
Project nummer	86578
Naam Hoofdaanvrager	Prof. dr. F.E. Van Leeuwen
Penvoerder / Bestuurlijk verantwoordelijke organisatie	Prof. dr. F.E. van Leeuwen / Prof. dr. E. Voest

Naam van organisatie	Type organisatie
Nederlands Kanker Instituut	Onderzoeksorganisatie

KOSTEN - Personeelskosten

Partner die de kosten maakt	Activiteiten (steekwoorden)	Functie	jaar 1	jaar 2	jaar 3	jaar 4	jaar 5	totaal van jaren	FTE %	Overhead opslag %	Subtotaal
Nederlands Kanker Instituut	Overall coordination, METC approval	Project coordinator	€ -	€ -	€ -	€ -	€ 413.280	€ 413.280	20%	0%	€ 82.656,00
Nederlands Kanker Instituut	Data collection, linkages and analysis	PhD student	€ -	€ -	€ -	€ -	€ 255.649	€ 255.649	100%	0%	€ 255.649,00
Nederlands Kanker Instituut	Supervision (cost-)effectiveness analysis	Coordinator cost-effectiveness analysis	€ -	€ -	€ -	€ -	€ 40.796	€ 40.796	20%	0%	€ 8.159,20
Nederlands Kanker Instituut	(Cost-)effectiveness analysis	Junior researcher cost-effectiveness	€ -	€ -	€ -	€ -	€ 34.046	€ 34.046	100%	0%	€ 34.046,00
Nederlands Kanker Instituut	Data abstraction	Research assistant	€ -	€ -	€ -	€ -	€ 206.852	€ 206.852	100%	0%	€ 206.852,00
			€ -	€ -	€ -	€ -	€ -	€ -	100%	0%	€ -
			€ -	€ -	€ -	€ -	€ -	€ -	100%	0%	€ -
			€ -	€ -	€ -	€ -	€ -	€ -	100%	0%	€ -
			€ -	€ -	€ -	€ -	€ -	€ -	100%	0%	€ -
			€ -	€ -	€ -	€ -	€ -	€ -	100%	0%	€ -
			€ -	€ -	€ -	€ -	€ -	€ -	100%	0%	€ -
			€ -	€ -	€ -	€ -	€ -	€ -	100%	0%	€ -
			€ -	€ -	€ -	€ -	€ -	€ -	100%	0%	€ -
			€ -	€ -	€ -	€ -	€ -	€ -	100%	0%	€ -
			€ -	€ -	€ -	€ -	€ -	€ -	100%	0%	€ -
			€ -	€ -	€ -	€ -	€ -	€ -	100%	0%	€ -
			€ -	€ -	€ -	€ -	€ -	€ -	100%	0%	€ -
			€ -	€ -	€ -	€ -	€ -	€ -	100%	0%	€ -
			€ -	€ -	€ -	€ -	€ -	€ -	100%	0%	€ -
			€ -	€ -	€ -	€ -	€ -	€ -	100%	0%	€ -

KOSTEN - Materiaalkosten & apparatuurkosten

Partner die de kosten maakt	Omschrijving	type	investeringsbedrag	aanschaf jaar (1-5)	Afschr. jaar 1	Afschr. jaar 2	Afschr. jaar 3	Afschr. jaar 4	Afschr. jaar 5	Subtotaal kosten
			€ -		€ -	€ -	€ -	€ -	€ -	€ -
			€ -		€ -	€ -	€ -	€ -	€ -	€ -
			€ -		€ -	€ -	€ -	€ -	€ -	€ -
			€ -		€ -	€ -	€ -	€ -	€ -	€ -
			€ -		€ -	€ -	€ -	€ -	€ -	€ -
			€ -		€ -	€ -	€ -	€ -	€ -	€ -
			€ -		€ -	€ -	€ -	€ -	€ -	€ -
			€ -		€ -	€ -	€ -	€ -	€ -	€ -
			€ -		€ -	€ -	€ -	€ -	€ -	€ -
			€ -		€ -	€ -	€ -	€ -	€ -	€ -
			€ -		€ -	€ -	€ -	€ -	€ -	€ -
			€ -		€ -	€ -	€ -	€ -	€ -	€ -
			€ -		€ -	€ -	€ -	€ -	€ -	€ -
			€ -		€ -	€ -	€ -	€ -	€ -	€ -
			€ -		€ -	€ -	€ -	€ -	€ -	€ -
			€ -		€ -	€ -	€ -	€ -	€ -	€ -
			€ -		€ -	€ -	€ -	€ -	€ -	€ -
			€ -		€ -	€ -	€ -	€ -	€ -	€ -
			€ -		€ -	€ -	€ -	€ -	€ -	€ -
			€ -		€ -	€ -	€ -	€ -	€ -	€ -

KOSTEN - Implementatiekosten

Partner die de kosten maakt	Omschrijving	jaar 1	jaar 2	jaar 3	jaar 4	jaar 5	Subtotaal
		€ -	€ -	€ -	€ -	€ -	€ -
		€ -	€ -	€ -	€ -	€ -	€ -
		€ -	€ -	€ -	€ -	€ -	€ -
		€ -	€ -	€ -	€ -	€ -	€ -
		€ -	€ -	€ -	€ -	€ -	€ -
		€ -	€ -	€ -	€ -	€ -	€ -
		€ -	€ -	€ -	€ -	€ -	€ -
		€ -	€ -	€ -	€ -	€ -	€ -
		€ -	€ -	€ -	€ -	€ -	€ -
		€ -	€ -	€ -	€ -	€ -	€ -

Budgetformat	Evaluatieonderzoek ZE&GG - extra ronde 2019
Datum	
Project naam	ationwide long-term follow-up care for lymphoma survivors in t
Project nummer	86578
Naam Hoofdaanvrager	Prof. dr. F.E. Van Leeuwen
Penvoerder / Bestuurlijk verantwoordelijke organisatie	Prof. dr. F.E. van Leeuwen / Prof. dr. E. Voest

		€ -	€ -	€ -	€ -	€ -	€ -
		€ -	€ -	€ -	€ -	€ -	€ -
		€ -	€ -	€ -	€ -	€ -	€ -
		€ -	€ -	€ -	€ -	€ -	€ -
		€ -	€ -	€ -	€ -	€ -	€ -
		€ -	€ -	€ -	€ -	€ -	€ -
		€ -	€ -	€ -	€ -	€ -	€ -
		€ -	€ -	€ -	€ -	€ -	€ -
		€ -	€ -	€ -	€ -	€ -	€ -
		€ -	€ -	€ -	€ -	€ -	€ -

KOSTEN - Overige kosten

Partner die de kosten maakt	Omschrijving	jaar 1	jaar 2	jaar 3	jaar 4	jaar 5	Subtotaal
Nederlands Kanker Instituut	METC approval	€ 2.000	€ -	€ -	€ -	€ -	€ 2.000,00
Nederlands Kanker Instituut	Travel expenses for data collection	€ 1.750	€ 3.500	€ 3.500	€ 875	€ -	€ 9.625,00
Nederlands Kanker Instituut	Patient participation		€ 250	€ 250	€ 500	€ 500	€ 1.500,00
Nederlands Kanker Instituut	Open access publication	€ -	€ -	€ -		€ 5.000	€ 5.000,00
Nederlands Kanker Instituut	Linkages with (disease) registries	€ -	€ -	€ 10.000	€ 10.000	€ -	€ 20.000,00
		€ -	€ -	€ -	€ -	€ -	€ -
		€ -	€ -	€ -	€ -	€ -	€ -
		€ -	€ -	€ -	€ -	€ -	€ -
		€ -	€ -	€ -	€ -	€ -	€ -
		€ -	€ -	€ -	€ -	€ -	€ -
		€ -	€ -	€ -	€ -	€ -	€ -
		€ -	€ -	€ -	€ -	€ -	€ -
		€ -	€ -	€ -	€ -	€ -	€ -
		€ -	€ -	€ -	€ -	€ -	€ -
		€ -	€ -	€ -	€ -	€ -	€ -
		€ -	€ -	€ -	€ -	€ -	€ -
		€ -	€ -	€ -	€ -	€ -	€ -
		€ -	€ -	€ -	€ -	€ -	€ -
		€ -	€ -	€ -	€ -	€ -	€ -
		€ -	€ -	€ -	€ -	€ -	€ -

Samenvatting kosten soorten

Totaal personeelskosten	€ 587.362,20
Totaal materiaalkosten & apparatuurkosten	€ -
Totaal implementatiekosten	€ -
Totaal overige kosten	€ 38.125,00
Totaal projectkosten	€ 625.487,20

Cofinanciering

Organisatie	Type organisatie	Beschrijving van bijdrage	jaar 1	jaar 2	jaar 3	jaar 4	jaar 5	IN KIND	IN CASH	Subtotaal
			€ -	€ -	€ -	€ -	€ -	€ -	€ -	€ -
			€ -	€ -	€ -	€ -	€ -	€ -	€ -	€ -
			€ -	€ -	€ -	€ -	€ -	€ -	€ -	€ -
			€ -	€ -	€ -	€ -	€ -	€ -	€ -	€ -
			€ -	€ -	€ -	€ -	€ -	€ -	€ -	€ -
			€ -	€ -	€ -	€ -	€ -	€ -	€ -	€ -
			€ -	€ -	€ -	€ -	€ -	€ -	€ -	€ -
			€ -	€ -	€ -	€ -	€ -	€ -	€ -	€ -
			€ -	€ -	€ -	€ -	€ -	€ -	€ -	€ -
			€ -	€ -	€ -	€ -	€ -	€ -	€ -	€ -

Matchings % Evaluation of nationwide long-term follow-up care for lymphoma survivors in the Netherlands Onderzoeksorganisatie	Matchings % Evaluation of nationwide long-term follow-up care for lymphoma survivors in the Netherlands kleine onderneming	Matchings % Evaluation of nationwide long-term follow-up care for lymphoma survivors in the Netherlands middelgrote onderneming	Matchings % Evaluation of nationwide long-term follow-up care for lymphoma survivors in the Netherlands grote onderneming	Matchings % Evaluation of nationwide long-term follow-up care for lymphoma survivors in the Netherlands Overig
0,00%	0,00%	0,00%	0,00%	0,00%

Matchings % Evaluation of nationwide long-term follow-up care for lymphoma survivors in the Netherlands Totaal	% bijdrage door ZonMw
0,00%	100,00%

**Totaal cofianciering**	€ -
**Totaal aangevraagde subsidie**	€ 625.487,20
**Financieringstekort**	€ -

Eindafrekening format

**Project naam**	Evaluation of nationwide long-ter
**Project nummer**	86578
**Naam Hoofdaanvrager**	Prof. dr. F.E. Van Leeuwen
**Penvoerder**	Prof. dr. F.E. van Leeuwen / Prof.

Naam van organisatie	Type organisatie
Nederlands Kanker Instituut	Onderzoeksorganisatie
-	-
-	-
-	-
-	-
-	-
-	-
-	-
-	-
-	-

KOSTEN - Personeelskosten

Partner die de kosten maakt	Activiteiten (steekwoorden)	Functie	Begroting	Realisatie	Verschil	Verklaring / Toelichting
Nederlands Kanker Instituut	Overall coordination, METC approval	Project coordinator	82.656,00	-	82.656,00	0,2 FTE for 4,5 years
Nederlands Kanker Instituut	Data collection, linkages and analysis	PhD student	255.649,00	-	255.649,00	1,0 FTE for 4,0 years
Nederlands Kanker Instituut	Supervision (cost-)effectiveness analy	Coordinator cost-effe	8.159,20	-	8.159,20	0,2 FTE for 0,5 year
Nederlands Kanker Instituut	(Cost-)effectiveness analysis	Junior researcher cos	34.046,00	-	34.046,00	1,0 FTE for 0,5 year
Nederlands Kanker Instituut	Data abstraction	Research assistant	206.852,00	-	206.852,00	1,0 FTE for 2,75 years
-	-	-	-	-	-	
-	-	-	-	-	-	
-	-	-	-	-	-	
-	-	-	-	-	-	
-	-	-	-	-	-	
-	-	-	-	-	-	
-	-	-	-	-	-	
-	-	-	-	-	-	
-	-	-	-	-	-	
-	-	-	-	-	-	
-	-	-	-	-	-	
-	-	-	-	-	-	
-	-	-	-	-	-	
-	-	-	-	-	-	
-	-	-	-	-	-	

KOSTEN - Materiaalkosten & apparatuurkosten

Partner die de kosten maakt	Omschrijving	type	Begroting	Realisatie	Verschil	Verklaring / Toelichting
-	-	-	-	-	-	
-	-	-	-	-	-	
-	-	-	-	-	-	
-	-	-	-	-	-	
-	-	-	-	-	-	
-	-	-	-	-	-	
-	-	-	-	-	-	
-	-	-	-	-	-	
-	-	-	-	-	-	
-	-	-	-	-	-	
-	-	-	-	-	-	
-	-	-	-	-	-	
-	-	-	-	-	-	
-	-	-	-	-	-	
-	-	-	-	-	-	
-	-	-	-	-	-	
-	-	-	-	-	-	
-	-	-	-	-	-	
-	-	-	-	-	-	
-	-	-	-	-	-	

KOSTEN - Implementatiekosten

Partner die de kosten maakt	Omschrijving		Begroting	Realisatie	Verschil	Verklaring / Toelichting
-	-	-	-	-	-	
-	-	-	-	-	-	
-	-	-	-	-	-	
-	-	-	-	-	-	
-	-	-	-	-	-	
-	-	-	-	-	-	
-	-	-	-	-	-	
-	-	-	-	-	-	
-	-	-	-	-	-	
-	-	-	-	-	-	
-	-	-	-	-	-	
-	-	-	-	-	-	
-	-	-	-	-	-	
-	-	-	-	-	-	
-	-	-	-	-	-	
-	-	-	-	-	-	
-	-	-	-	-	-	
-	-	-	-	-	-	
-	-	-	-	-	-	
-	-	-	-	-	-	

KOSTEN - Overige kosten

Partner die de kosten maakt	Omschrijving		Begroting	Realisatie	Verschil	Verklaring / Toelichting
Nederlands Kanker Instituut	METC approval	-	2.000,00	-	2.000,00	
Nederlands Kanker Instituut	Travel expenses for data collection	-	9.625,00	-	9.625,00	
Nederlands Kanker Instituut	Patient participation	-	1.500,00	-	1.500,00	
Nederlands Kanker Instituut	Open access publication	-	5.000,00	-	5.000,00	
Nederlands Kanker Instituut	Linkages with (disease) registries	-	20.000,00	-	20.000,00	
-	-	-	-	-	-	
-	-	-	-	-	-	
-	-	-	-	-	-	
-	-	-	-	-	-	
-	-	-	-	-	-	
-	-	-	-	-	-	
-	-	-	-	-	-	
-	-	-	-	-	-	
-	-	-	-	-	-	
-	-	-	-	-	-	
-	-	-	-	-	-	
-	-	-	-	-	-	
-	-	-	-	-	-	
-	-	-	-	-	-	
-	-	-	-	-	-	

Samenvatting kosten soorten

	Begroting	Realisatie	Verschil
**Totaal personeelskosten**	587.362,20	-	587.362,20
**Totaal materiaalkosten & apparatuurkosten**	-	-	-
**Totaal implementatiekosten**	-	-	-
**Totaal overige kosten**	38.125,00	-	38.125,00
**Totaal projectkosten**	625.487,20	-	625.487,20

Cofinanciering

Organisatie	Type organisatie		Begroting	Realisatie	Verschil	Verklaring / Toelichting
0	0		-	-	-	

0	0	-	-	-	
0	0	-	-	-	
0	0	-	-	-	
0	0	-	-	-	
0	0	-	-	-	
0	0	-	-	-	
0	0	-	-	-	
0	0	-	-	-	
0	0	-	-	-	

APPENDI□: Estimation of potential health care efficiency gain

Number of patients, health gains and cost-savings

PLEASE NOTICE: This is a mandatory appendix. You need to add this file (as a PDF file) as an attachment to your application in ProjectNet. The maximum length of the file is 2 pages (A4) and you are not allowed to change the format. Make sure your text remains visible in the given text fields!

1) Yearly number of patients in the Netherlands that will receive intervention targeted in this proposal

	Value	*Population / Time period*
Incidence (if applicable)		

The incidence is the number of new cases that develop a disease in a particular population in a given period of time.

	Value	*Population / Year*
Prevalence (if applicable)	20500	2020

The prevalence is the total number of cases of a disease that is present in a particular population at a particular time.

Yearly number of patients in the Netherlands (a): 20.500

Please explain numbers quoted above (max. 500 characters) — *characters left: 227*

The number of 5-year Hodgkin lymphoma (HL) and diffuse large B-cell lymphoma (DLBCL) survivors eligible for BETER survivorship care in the Netherlands in 2020 is estimated from the incidence and survival rates of HL and DLBCL in the Netherlands for the period of 1961-2017.

References or sources used (max. 500 characters) — *characters left: 438*

www.cijfersoverkanker.nl from The Netherlands Cancer Registry.

2) Estimate the potential effects on health from the intervention(s) that will be evaluated in this proposal compared to usual care. Preferably in (quality adjusted) life years, (QA)LY's, or another generic measure. This should be the primary outcome measure in your proposal (or related to).

	Value	*Outcome measure (QALY's/other generic measure)*
Effect of usual care (b)	1,18	DALYs in 5 years
Effect of intervention to be evaluated (c)	0,56	DALYs in 5 years
Potential effect on health of new intervention compared to usual care, patientlevel (c-b)	-0,62	DALYs in 5 years

Please explain numbers quoted above (max. 500 characters) — *characters left: 6*

A Hodgkin lymphoma (HL) survivor, who is not screened for late adverse treatment events, acquires 1,18 DALYs in 5 years (1,12 for breast cancer + cardiovascular disease; 0,01 for hypothyroidism; 0,04 for functional asplenia), as opposed to 0,56 DALYs for a survivor screened at a BETER clinic (0,55 for breast cancer + cardiovascular disease; 0,00 for hypothyroidism; 0,00 for functional asplenia). Screening at the BETER clinics averts on average 0,62 DALYs per HL survivor in a 5-year period.

References or sources used (max. 500 characters) characters left: 205

Proportions were extracted from PMID 25915855, 19667275, 1861693, 15127830 and risk table cardiovascular risk, version 2016. Disability weights for cardiovascular disease were extracted from PMID 27551405, 26475018. Number of DALYs averted with an extensive breast cancer program: PMID 16430401.

3) Estimate the potential effects on costs (€) associated with the intervention(s) that will be evaluated in this proposal compared to usual care. The costs should at least be presented from the healthcare perspective. If relevant, other perspectives like the institution or societal perspective could be presented.

	Healthcare perspective	Other perspective: State perspective...
Costs usual care per patient per year (d)	€ 1.267,00	€
Costs intervention that will be evaluated, per patient per year €	€ 996,00	€
Potential cost-savings of new intervention: yearly population(a)*costsaving per patient per year(d-e)	*Based on 100% implementation* € **5.555.500**	€ **0**
Expected (realistic) estimate of % implementation 3 years after closure of study	80%	
Potential cost-savings of new intervention: yearly population(a)*costsaving per patient per year(d-e)	*Corrected for % implementation* € **4.444.400**	€ **0**

Please explain numbers quoted above (max. 650 characters) characters left: 4

Usual care (UC) consist of: 20% early detected Late Effects (LE) for Cardio vasculair disease (CVD) of E160/y and 80% late detected CVD LE of E501/y. For the BETER strategy (E777/5y), these proportions were 75% for early and 25% for late LE. Breast cancer burden pppy was estimated based on Vondeling ea, 2018: E700M divided over 111k cases of breast cancer in the NL in 2011. For hyperthyroidism, drug costs (E22) were calculated over 31,5y for the UC group, and for 33y for BETER. For asplenia, vaccinations costs (E290) were calculated for the BETER strategy only. Finally, a combination of CVD and breast cancer was taken into account for 3%.

References or sources used (max. 500 characters) characters left: 158

Drug costs were based on www.medicijnkosten.nl; intervention costs were based on Dutch "passantentarieven" of cardiologiecentra CCN-2018; proportions were based on several publications on CVD, breast cancer, hyperthyroidism and asplenia a.o.: PMID 2915855, 19667275, 1861693, 15127830. Breast cancer burden PMID 29514651 (Vondeling ea, 2018).

4) Summary of potential health care efficiency gain / cost savings (max. 1000 characters)

characters left: 312

Based on the available data, the costs saved in health care only are estimated to amount up to E4.4 million with an implementation rate of 80%. This is an underestimation of the total costs, as societal costs were not taken into account (currently unknown). In a sample of 300 lymphoma survivors, screened for a period of 5 years, 185 DALYs are estimated to be averted. The benefit of survivorship care according to the BETER guidelines is expected to improve even more when survivors are all included directly after their 5-year follow-up for recurrence (on average at the age of 33 for Hodgkin lymphoma survivors instead of the current 52 years), which is the goal of the BETER project.

Bijlage 3

VERKLARING WETENSCHAPPELIJKE/ BEROEPS VERENIGING MBT PRIORITERING, INCLUSIES EN IMPLEMENTATIE

Achtergrond

Het programma Zorgevaluatie en Gepast Gebruik heeft als doel de vraagstukken te evalueren die er toe doen. Binnen de wetenschappelijke verenigingen, beroepsverenigingen en patiëntenverenigingen is de afgelopen jaren reeds ingezet in het vaststellen van een kennisagenda met kennisvragen die volgens de leden nader onderzocht moeten worden. Evaluatieonderzoeken naar onderwerpen die voorkomen op een van de kennisagenda's ontvangen daarmee reeds steun nog voor de start van het onderzoek.
De voorwaarden voor de indiening en toekenning van een evaluatieonderzoek staan beschreven in de subsidie oproep **Zorgevaluatie en Gepast Gebruik – evaluatieonderzoek extra ronde 2019**.

De betrokkenheid van wetenschappelijke verenigingen en/of beroepsverenigingen bij de evaluatieonderzoeken heeft belangrijke consequenties voor de uitvoer de projecten en de implementatie van de toekomstige resultaten. Gegeven het draagvlak binnen de beroepsgroep en/of de wetenschappelijke vereniging, zullen zij zich dan ook rechtstreeks inspannen voor de uitvoering van het evaluatieonderzoek en voor de implementatie van de resultaten. Het evaluatieonderzoek wordt uitgevoerd door een netwerk, gedragen door de betrokken Wetenschappelijke vereniging of beroepsvereniging. De betrokken Wetenschappelijke vereniging of beroepsvereniging en de hoofdaanvrager staan samen garant voor de benodigde inclusie van patiënten.

Verklaring

Het bestuur van de Wetenschappelijke vereniging en/of beroepsvereniging NVRO verklaart in relatie tot het evaluatieonderzoek **"Evaluation of nationwide long-term follow-up care for lymphoma survivors in the Netherlands: does survivorship care at the BETER clinics reduce morbidity and mortality from late effects of lymphoma treatment and associated costs?"** met als hoofdaanvrager mw. prof. dr. F.E. van Leeuwen, hoofd afdeling epidemiologie, Nederlands Kanker Instituut, Amsterdam en als mede projectaanvrager mw. dr. B.M.P. Aleman, radiotherapeut-oncoloog, Nederlands Kanker Instituut, Amsterdam,

- Garant te staan voor het belang van de kennislacune die de basis heeft gevormd tot de uitgewerkte subsidieaanvraag voor evaluatieonderzoek
- Zich tot het uiterste in te spannen, samen met de hoofdaanvrager, om de beoogde inclusies binnen het genoemde tijdsbestek van het betreffende evaluatieonderzoek te realiseren;
- Garant te staan voor implementatie van de resultaten van het evaluatieonderzoek in de relevante richtlijn(en) binnen twaalf maanden na goedkeuring van het eindverslag van het evaluatieonderzoek.

Datum: 16/3/2020

Ondertekening: [signature]

Functie: voorzitter commissie kwaliteit/ lid bestuur NVRO

Bijlage 3

VERKLARING WETENSCHAPPELIJKE/ BEROEPS VERENIGING MBT PRIORITERING, INCLUSIES EN IMPLEMENTATIE

Achtergrond

Het programma Zorgevaluatie en Gepast Gebruik heeft als doel de vraagstukken te evalueren die er toe doen. Binnen de wetenschappelijke verenigingen, beroepsverenigingen en patiëntenverenigingen is de afgelopen jaren reeds ingezet in het vaststellen van een kennisagenda met kennisvragen die volgens de leden nader onderzocht moeten worden. Evaluatieonderzoeken naar onderwerpen die voorkomen op een van de kennisagenda's ontvangen daarmee reeds steun nog voor de start van het onderzoek.
De voorwaarden voor de indiening en toekenning van een evaluatieonderzoek staan beschreven in de subsidie oproep **Zorgevaluatie en Gepast Gebruik – evaluatieonderzoek extra ronde 2019**.

De betrokkenheid van wetenschappelijke verenigingen en/of beroepsverenigingen bij de evaluatieonderzoeken heeft belangrijke consequenties voor de uitvoer de projecten en de implementatie van de toekomstige resultaten. Gegeven het draagvlak binnen de beroepsgroep en/of de wetenschappelijke vereniging, zullen zij zich dan ook rechtstreeks inspannen voor de uitvoering van het evaluatieonderzoek en voor de implementatie van de resultaten. Het evaluatieonderzoek wordt uitgevoerd door een netwerk, gedragen door de betrokken Wetenschappelijke vereniging of beroepsvereniging. De betrokken Wetenschappelijke vereniging of beroepsvereniging en de hoofdaanvrager staan samen garant voor de benodigde inclusie van patiënten.

Verklaring

Het bestuur van de Wetenschappelijke vereniging en/of beroepsvereniging NVvH verklaart in relatie tot het evaluatieonderzoek "**Evaluation of nationwide long-term follow-up care for lymphoma survivors in the Netherlands: does survivorship care at the BETER clinics reduce morbidity and mortality from late effects of lymphoma treatment and associated costs?"** met als hoofdaanvrager mw. prof. dr. F.E. van Leeuwen, hoofd afdeling epidemiologie, Nederlands Kanker Instituut, Amsterdam en als co-project leider mw. prof. dr. J.M. Zijlstra hematoloog in Amsterdam UMC, Amsterdam,

- Garant te staan voor het belang van de kennislacune die de basis heeft gevormd tot de uitgewerkte subsidieaanvraag voor evaluatieonderzoek
- Zich tot het uiterste in te spannen, samen met de hoofdaanvrager, om de beoogde inclusies binnen het genoemde tijdsbestek van het betreffende evaluatieonderzoek te realiseren;
- Garant te staan voor implementatie van de resultaten van het evaluatieonderzoek in de relevante richtlijn(en) binnen twaalf maanden na goedkeuring van het eindverslag van het evaluatieonderzoek.

Datum:	24 april 2020
Ondertekening:	Prof. dr. Karina Meijer
Functie:	Secretaris NVvH

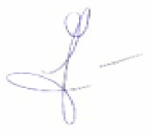

Bijlage: CONSORT Flow-diagram

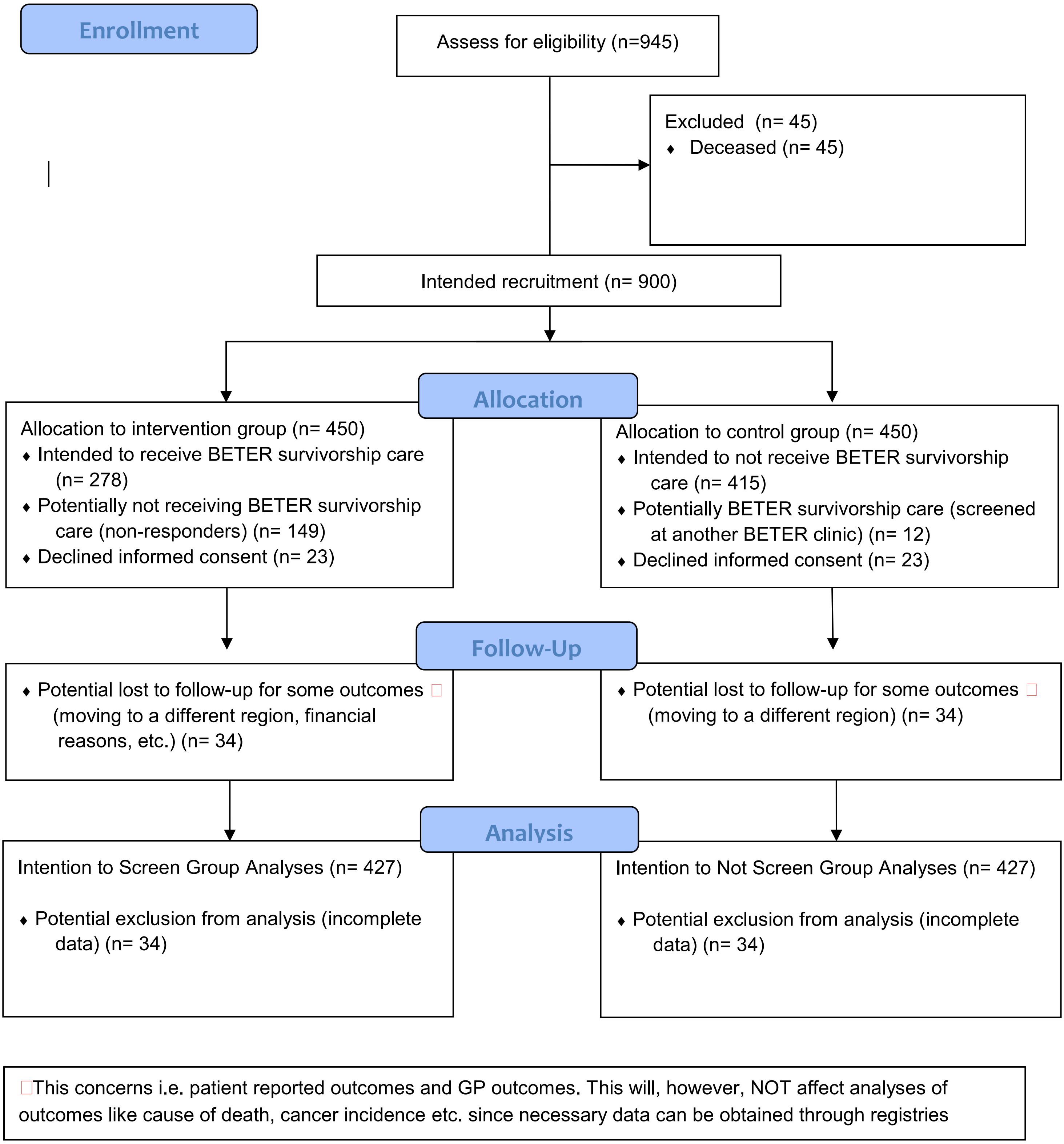

BETER centres including patients in the study:

BETER clinic started in 2013-2014	BETER clinic will start in 2021-2024
Amsterdam UMC, location VUmc, Amsterdam	Amsterdam UMC, location Academisch Medisch Centrum, Amsterdam
Erasmus MC, Rotterdam	Leids Universitair Medisch Centrum, Leiden
Antoni van Leeuwenhoek, Amsterdam	Universitair Medisch Centrum Groningen
Universitair Medisch Centrum Utrecht	Catharina Ziekenhuis, Eindhoven
Radboudumc, Nijmegen	Hagaziekenhuis, Den Haag
Instituut Verbeeten, Tilburg/Den Bosch/Breda	Haaglanden Medisch Centrum, Den Haag
	St. Antonius Ziekenhuis, Nieuwegein

Informatie voor deelnemers
aan medisch-wetenschappelijk onderzoek

BETER-EVALUATIE studie

Evaluatie van zorg op de BETER-Poli's

Inleiding

Geachte heer/mevrouw,

Wij willen u vragen om mee te doen aan een medisch-wetenschappelijk onderzoek. Meedoen is vrijwillig. Om mee te doen is wel uw schriftelijke toestemming nodig. U ontvangt deze brief omdat u onder controle bent bij de BETER polikliniek in [ziekenhuis]. Voordat u beslist of u wilt meedoen aan dit onderzoek, leggen wij u in deze brief uit wat het onderzoek voor u inhoudt. Lees deze informatie rustig door en vraag uw arts of de onderzoeker om uitleg als u vragen heeft. U kunt ook de onafhankelijk deskundige, die aan het eind van deze brief genoemd wordt, om aanvullende informatie vragen. U kunt er ook over praten met uw partner, vrienden of familie. Verdere informatie over meedoen aan onderzoek staat in de bijgevoegde brochure 'Medisch-wetenschappelijk onderzoek'.

Dit onderzoek voldoet aan de voorwaarden gesteld in de Wet op het Medisch Wetenschappelijk Onderzoek (WMO). De WMO noemt mensen die meedoen aan medisch wetenschappelijk onderzoek 'proefpersonen'. Wij vinden de term 'proefpersoon' bij dit wetenschappelijke onderzoek niet passend omdat er niet een medicijn wordt getest bij de deelnemers aan het onderzoek en aan hen niet gevraagd wordt om mee te doen aan een experiment. Om deze reden zullen wij in de rest van deze informatiebrief spreken over 'deelnemers aan het onderzoek'. Het is echter wel van belang dat u zich hierbij realiseert dat u, ondanks de andere bewoording, deelneemt aan medisch wetenschappelijk onderzoek.

1. Algemene informatie

Dit onderzoek is opgezet door het Antoni van Leeuwenhoek en is onderdeel van het BETER-project: **B**etere zorg na (non-)Hodgkinlymfoom: **E**valuatie van en screening op lange **T**ermijn **E**ffecten van chemotherapie en **R**adiotherapie. Het onderzoek vindt plaats in verschillende ziekenhuizen, waaronder uw ziekenhuis, en wordt door artsen, verpleegkundig specialisten en onderzoekers uitgevoerd. In totaal zullen er ongeveer 900 mensen meedoen.
De medisch-ethische toetsingscommissie van het Antoni van Leeuwenhoek heeft dit onderzoek goedgekeurd. Algemene informatie over de toetsing van onderzoek vindt u in de brochure 'Medisch-wetenschappelijk onderzoek'.

2. Achtergrond van het onderzoek

De meeste patiënten met Hodgkinlymfoom kunnen tegenwoordig worden genezen door combinaties van chemotherapie, radiotherapie en immunotherapie. Hoewel de behandeling van Hodgkinlymfoom als doel heeft de kwaadaardige cellen te vernietigen, gaat dit helaas gepaard met het beschadigen van gezonde cellen. Deze beschadigingen kunnen maanden tot nog vele jaren later gezondheidsproblemen veroorzaken, de zogenaamde late effecten van de behandeling (bijvoorbeeld hart- en vaatziekten en nieuwe vormen van kanker). De BETER poliklinieken zijn opgezet om deze gezondheidsproblemen eerder te ontdekken zodat ze beter te behandelen zijn. De eerste BETER poli's zijn gestart in 2013, nadat er in Nederland richtlijnen waren opgesteld voor nazorg gericht op late effecten van lymfklierkankerbehandeling. Maar we weten eigenlijk niet hoe goed we er in slagen, door de richtlijnen te volgen, om ziekte en sterfte door late effecten van behandeling te voorkómen. Dat komt ook doordat Nederland het eerste land is waar speciale nazorg voor overlevers van Hodgkinlymfoom is opgezet. Daarom doen we nu in zes ziekenhuizen een evaluatieonderzoek bij de eerste groep patiënten die voor de BETER polikliniek zijn uitgenodigd, in de periode 2013-2016.
We hopen dat dit onderzoek resultaten zal opleveren waardoor we de richtlijnen en de verdere zorg op de BETER poli's verder kunnen verbeteren.

3. Doel van het onderzoek

Het doel van dit onderzoek is om na te gaan hoeveel afwijkingen en ziekten er bij het screeningsonderzoek op de BETER poliklinieken worden gevonden. We willen ook weten in hoeverre de gevonden afwijkingen en ziekten minder ernstig zijn dan bij een vergelijkingsgroep van overlevers van Hodgkinlymfoom die nog nooit op een BETER poli zijn geweest, omdat de ziekenhuizen waar zij zijn behandeld nog geen BETER poli hebben gestart. Verder willen we weten hoeveel kosten er in de zorg gespaard zijn door de zorg op de BETER poli's. Tenslotte willen we onderzoeken of de zorg op de BETER poli invloed heeft gehad op uw kwaliteit van leven, uw kennis over late effecten van behandeling en uw zorgen over mogelijke late effecten.

4. Wat meedoen inhoudt

Als u meedoet, dan vragen we u een vragenlijst in te vullen over de aandoeningen die na uw laatste bezoek aan de BETER poli bij u zijn gevonden (door huisarts of specialist), uw medicatiegebruik, bezoeken aan de huisarts of andere ziekenhuizen dan waar de BETER poli is, en deelname aan bevolkingsonderzoeken. Daarnaast vragen we u vragenlijsten in te vullen over wat u weet over late effecten van behandeling, hoe hoog u denkt dat de risico's zijn, of u zich daar veel of weinig zorgen over maakt, of u tevreden bent met de zorg op de BETER poli. Verder vragen we naar uw kwaliteit van leven. Tenslotte, willen we ook graag weten wat we aan de zorg op de BETER poli zouden kunnen verbeteren.

We vragen u tevens om toestemming te geven voor het verzamelen van gegevens uit uw medisch dossier (door medewerkers van de BETER-Evaluatie studie) en voor het opvragen van gegevens over uw gezondheid bij uw huisarts en/of uw behandeld specialist in een ander ziekenhuis. We vragen u ook om toestemming voor het opvragen van gegevens over eventuele ziektes bij een aantal ziekteregistraties zoals de Nederlandse Kankerregistratie, het Pathologisch-Anatomisch Landelijk Geautomatiseerd Archief (PALGA, registratie voor weefseluitslagen), Dutch Hospital Data (registratie ontslagdiagnose ziekenhuizen) en de Nederlandse Hart Registratie (NHR). Mocht u tijdens de looptijd van het onderzoek overlijden, dan willen wij de officiële doodsoorzaak opvragen bij het Centraal Bureau voor de Statistiek (CBS).

5. Mogelijke voor- en nadelen

Het is belangrijk dat u de mogelijke voor- en nadelen van dit onderzoek goed afweegt voordat u besluit mee te doen. Er zijn voor u geen nadelen aan deelname verbonden, behalve dat het invullen van de vragenlijsten u wat tijd kost, wij schatten een uur. U heeft zelf ook geen voordeel van meedoen aan dit onderzoek. Uw deelname kan wel bijdragen aan meer kennis over de late gevolgen van de behandeling van lymfeklierkanker en aan verbetering van de zorg op de BETER poliklinieken.

6. Als u niet wilt meedoen

U beslist zelf of u meedoet aan het onderzoek. Deelname is vrijwillig. Als u niet wilt meedoen, heeft dat geen gevolgen voor eventuele zorg en behandelingen die u nu of in de toekomst nodig heeft.

7. Eind van het onderzoek

Het hele onderzoek is afgelopen wanneer de gegevens van alle deelnemers geanalyseerd zijn. Een samenvatting van het onderzoek zal te lezen zijn op de website van het BETER-project (www.beternahodgkin.nl).

8. Gebruik uw gegevens, vertrouwelijkheid van gegevens, en bewaartermijn

Voor dit onderzoek worden uw persoonsgegevens en lichaamsmateriaal verzameld, gebruikt en bewaard. Het gaat om gegevens zoals uw naam, adres, geboortedatum en om gegevens over uw gezondheid. Het verzamelen, gebruiken en bewaren van uw gegevens is nodig om de vragen die in dit onderzoek worden gesteld te kunnen beantwoorden en de resultaten te kunnen publiceren.

Om uw privacy te waarborgen, worden uw gegevens voorzien van een code. Uw naam en andere gegevens die u direct kunnen identificeren worden apart bewaard, dus niet samen met de onderzoeksgegevens. Alleen met de sleutel van de code zijn gegevens tot u te herleiden en de sleutel van de code blijft bij de onderzoeker. De resultaten van het onderzoek zullen in wetenschappelijke tijdschriften en rapporten worden gepubliceerd, maar uw gegevens daarin zijn nooit naar u persoonlijk te herleiden. U zult dus nooit als persoon herkend worden.

Uw gegevens worden 15 jaar bewaard op de onderzoekslocatie (uw ziekenhuis dan wel het Antoni van Leeuwenhoek).

9. Intrekken toestemming

U kunt uw toestemming voor gebruik van uw persoonsgegevens altijd weer intrekken. Dit geldt voor dit onderzoek en ook voor het bewaren en het gebruik voor het toekomstige onderzoek.

10. Meer informatie over uw rechten bij verwerking van gegevens

Voor algemene informatie over uw rechten bij verwerking van uw persoonsgegevens kunt u de website van de Autoriteit Persoonsgegevens raadplegen.
Bij vragen over uw rechten kunt u contact opnemen met de verantwoordelijke voor de verwerking van uw persoonsgegevens. Voor dit onderzoek zijn het Antoni van Leeuwenhoek en uw ziekenhuis verantwoordelijk voor de naleving van de regels voor de verwerking van uw persoonsgegevens. Zie

bijlage A voor contactgegevens.
Bij vragen of klachten over de verwerking van uw persoonsgegevens raden we u aan eerst contact op te nemen met de onderzoekslocatie. U kunt ook contact opnemen met de Functionaris voor de Gegevensbescherming van het Antoni van Leeuwenhoek of uw ziekenhuis (zie bijlage A voor contactgegevens) of de Autoriteit Persoonsgegevens.

11. Heeft u vragen?

Bij vragen kunt u contact opnemen met de onderzoeker. Voor onafhankelijk advies over meedoen aan dit onderzoek kunt u terecht bij de onafhankelijke arts. Deze arts weet veel over het onderzoek, maar heeft niets te maken met dit onderzoek.
Indien u klachten heeft over het onderzoek, kunt u dit bespreken met de onderzoeker of uw behandelend arts. Wilt u dit liever niet, dan kunt u zich wenden tot de klachtenfunctionaris van uw ziekenhuis. Alle contactgegevens vindt u in **bijlage A**: Contactgegevens.

15. Ondertekening toestemmingsformulier

Wanneer u voldoende bedenktijd heeft gehad, wordt u gevraagd te beslissen over deelname aan dit onderzoek. Indien u toestemming geeft, zullen wij u vragen deze op de bijbehorende toestemmingsverklaring schriftelijk te bevestigen. Door uw schriftelijke toestemming geeft u aan dat u de informatie heeft begrepen en instemt met deelname aan het onderzoek.
Het handtekeningenblad wordt door de onderzoeker bewaard. U krijgt een kopie of een tweede exemplaar van deze toestemmingsverklaring.

Bijlage A: Contactgegevens

Contactgegevens van de onderzoekslocatie

- Indien u nog vragen of opmerkingen heeft over het onderzoek, dan kunt u een e-mail sturen naar….. of contact opnemen met de coördinator van de BETER-EVALUATIE studie via telefoonnummer …..

- Indien u een onafhankelijk advies wenst over deelname aan onderzoek kunt u contact opnemen met de onafhankelijk arts, die niet direct betrokken is bij het onderzoek:…..

- Indien u klachten heeft over de (uitvoering van) de studie, kunt u contact opnemen met de klachtenfunctionaris van uw ziekenhuis of van het Antoni van Leeuwenhoek. De Functionaris Gegevensbescherming van het Antoni van Leeuwenhoek kunt u bereiken via privacy@nki.nl. U kunt ook contact opnemen met het Centrum Patiënteninformatie in de centrale hal van het Antoni van Leeuwenhoek (Plesmanlaan 121, Amsterdam) of bellen naar 020-5129111.

Contactgegevens van het [uitnodigend ziekenhuis]
[betrokken BETER-arts]
[Antoni van Leeuwenhoek]
[Plesmanlaan 121]
[1066 CX Amsterdam]
[Tel.: 020-512 9111]

Bijlage C: toestemmingsformulier deelnemer aan medisch-wetenschappelijk onderzoek

BETER-EVALUATIE studie

Evaluatie van zorg op de BETER poli's

- Ik heb de informatiebrief gelezen. Ook kon ik vragen stellen. Mijn vragen zijn voldoende beantwoord. Ik had genoeg tijd om te beslissen of ik meedoe.
- Ik weet dat meedoen vrijwillig is. Ook weet ik dat ik op ieder moment kan beslissen om toch niet mee te doen of te stoppen met het onderzoek. Daarvoor hoef ik geen reden te geven.
- Ik geef toestemming om mijn huisarts en/of behandelend specialist te informeren over mijn deelname dit onderzoek.
- Ik geef toestemming voor inzage in mijn medisch dossier aan de onderzoekers die betrokken zijn bij deze studie, om informatie te verzamelen over mijn ziektegeschiedenis en behandelingen. Ik geef toestemming voor het opvragen van informatie bij mijn huisarts en/of behandelend specialist(en) als deze gegevens nodig zijn voor het beantwoorden van de onderzoeksvraag.
- Ik geef toestemming voor het verzamelen en gebruiken van mijn gegevens voor de beantwoording van de onderzoeksvraag in dit onderzoek.
- Ik geef toestemming voor het opvragen en gebruiken van mijn gegevens uit landelijke registraties, te weten: de Nederlandse Kankerregistratie (NKR), het Pathologisch-Anatomisch Landelijk Geautomatiseerd Archief (PALGA, registratie voor weefseluitslagen), de BETER registratie, de registratie voor ziekenhuisontslagen en de Nederlandse Hart Registratie (NHR). Ook geef ik toestemming om, in het geval ik tijdens de looptijd van het onderzoek zou komen te overlijden, mijn officiële doodsoorzaakgegevens op te vragen hij het Centraal Bureau voor de Statistiek.

Tekenpagina BETER Evaluatie studie:

- Ik wil meedoen aan dit onderzoek.

Deelnemer aan medisch-wetenschappelijk onderzoek:
Achternaam en voorletters: ……………………………….
Handtekening: ………………………………………………   Datum : … / … / …

BETER Evaluatie studie

Onderstaande wordt ingevuld door de onderzoeker of diens vertegenwoordiger:

Ik verklaar dat ik deze deelnemer aan medisch-wetenschappelijk onderzoek volledig heb geïnformeerd over het genoemde onderzoek. Als er tijdens het onderzoek informatie bekend wordt die de toestemming van de deelnemer aan medisch-wetenschappelijk onderzoek zou kunnen beïnvloeden, dan breng ik hem/haar daarvan tijdig op de hoogte.

Naam onderzoeker (of diens vertegenwoordiger): …………………………………..

Handtekening: ………………………………………………   Datum: … / … / …

Competence of the project leader Flora E. van Leeuwen

Prof. Dr. Flora van Leeuwen has been working in the Netherlands Cancer Institute (NKI) since 1981, when she initiated the NKI Epidemiology group. Over the years the group has expanded to 35 employees, and became part of the Division of Psychosocial Research and Epidemiology (75 employees), since 2010 headed by Flora van Leeuwen. She also has a Chair in Cancer Epidemiology at the Medical Faculty of Amsterdam University (formerly VUmc).

The Cancer Epidemiology group at NKI focuses on three principal research lines:

(1) The assessment of the long-term risks of second malignancy, cardiovascular disease and other adverse events following treatment of Hodgkin lymphoma, testicular cancer, breast cancer, non-Hodgkin lymphoma and childhood cancer; including development of risk prediction models allowing identification of high-risk groups;

(2) Screening for late effects of cancer treatment and implementation of cancer survivorship care, to reduce morbidity and mortality from late adverse effects of cancer treatment, in order to increase cancer survivors' life expectancy and quality of life;

(3) The assessment of the roles of hormone-related and genetic risk factors in the etiology of breast and ovarian cancer. Special interest is in cancer etiology in BRCA1/2 families.

Flora van Leeuwen has extensive experience in the conduct of large-scale cohort studies (both retrospective and prospective), linkage with disease registries, and nested case-control studies. She established several large nationwide cohorts of cancer patients (see below) and high-risk individuals from the population (BRCA mutation carriers). She has been very successful in bringing together clinicians and researchers from a large number of hospitals to collaborate in research and clinical activities in nationwide consortia, such as BETER (lymphoma survivorship care) and HEBON (Hereditary Breast and Ovarian cancer in the Netherlands).

Recently, she has also initiated and successfully completed screening studies in cancer survivors, focusing on the diagnostic value of different screening methods to early detect adverse events after cancer treatment (*Jacobse JN, et al. Eur J Heart Fail. 2020;22(2):338).* She has also been actively involved in intervention studies to reduce the risk of late effects of cancer treatment, e.g. she is co-principal investigator of an intervention study to reduce fatigue in Hodgkin lymphoma survivors (*Starreveld DEJ et al. BMC Cancer 2018, 10: 880*). Flora van Leeuwen has been awarded a large number of grants especially from the Dutch Cancer Society, on the risk of and risk factors for late adverse effects of cancer treatment and has successfully completed these studies. In 2011 she was awarded a Queen Wilhelmina Research Program Grant from the Dutch Cancer Society entitled "Assessment of late adverse events after treatment for Hodgkin lymphoma." In 2019, the U.S. National Cancer Institute awarded her the Rosalind Franklin award for Women in Cancer Research, for her national and international work on late effects of cancer treatment.

With respect to the first research line, now that curative treatment is available for a substantial group of cancer patients, it is increasingly important to evaluate to which extent the occurrence of late complications of treatment affects their long-term survival. To assess the risk of second cancers, cardiovascular disease and other comorbidities after radiotherapy and systemic treatments her research group established large patient cohorts with information on radiation fields and chemotherapy regimens. The nationwide Hodgkin lymphoma cohort includes 10,500 patients treated 1965-2009 and the testicular cancer cohort includes 8,500 patients treated 1966-2009. For breast cancer we have built a 2-hospital cohort of breast cancer patients treated 1970-2009 (N=26,000). Furthermore, Flora van Leeuwen co-established the Dutch Childhood Oncology LATER cohort of childhood cancer survivors (N=6,200). These cohorts are linked with the Netherlands Cancer Registry (NCR), Dutch Hospital Data, Statistics Netherlands (cause of death) and cardiovascular disease registries, but also actively followed through general practitioners. Radiation and drug dose-response relationships, influence of lifestyle and genetic susceptibility are investigated in case-control studies, in which patients are requested to complete questionnaires and donate blood. Also, samples of the cohorts are approached for medical assessments in the participating clinics, e.g. assessment of subclinical cardiovascular disease to examine the value of

cardiovascular screening in cancer survivors (*Jacobse JN, et al. Eur J Heart Fail. 2020;22(2):338).*

Important scientific achievements include improved insight into:

1. The magnitude of the risk of breast cancer after radiotherapy for Hodgkin lymphoma, including radiotherapy dose-response assessment *(Schaapveld M, et al. N Eng J Med. 2015;373(26):2499-2511; Krul IM, et al. Int J Radiat Oncol Biol Phys. 2017;99(4):843-853; van Leeuwen FE, et al. J Natl Cancer Inst. 2003;95(13):971-980; De Bruin ML, et al. J Clin Oncol. 2009;27(26):4229-4231).*
2. The effects of chemotherapy for Hodgkin lymphoma on second malignancy risk: a risk increase for gastrointestinal tract cancers and a decrease for breast cancer (mediated through the gonadotoxicity of chemotherapy) *(Morton LM, et al. J Clin Oncol. 2013;31(27):3369-3377; Schaapveld M, et al. N Eng J Med. 2015;373(26):2499-2511; Travis LB, et al. JAMA 2003;290(4):465-475; De Bruin ML, et al. J Clin Oncol. 2009;27(26):4229-4231).*
3. The magnitude of risk of various cardiovascular diseases after chemotherapy and radiotherapy for Hodgkin lymphoma, including radiation dose-response and radiation volume analysis *(van Nimwegen FA, et al. J Clin Oncol. 2016;34(3):235-243; van Nimwegen FA, et al. JAMA Int Med. 2015;175(6):1007-1017; van Nimwegen FA, et al. Blood 2017;129(16):2257-2265).*
4. The total burden of late adverse events after treatment for Hodgkin lymphoma and childhood cancer. *(de Vries S, et al. Br J Cancer 2018;118(6):887-895; Geenen MM, et al. JAMA 2007;297(24):2705-2715).*

With respect to the second research line: knowledge generated in the first research line, especially the high risk of late effects of treatment in Hodgkin lymphoma survivors, inspired Flora van Leeuwen to initiate the development of a nationwide survivorship care program for lymphoma survivors, in close collaboration with her clinical colleagues Berthe Aleman and John Raemaekers. The program is coordinated by her research group and by the department of Radiation Oncology, NKI. Starting in 2009, a consortium of hemato-oncologists and radiation oncologists of now 33 lymphoma treatment centres has been established, the BETER consortium (**B**etter care after Lymphoma: **E**valuation of long-term **T**reatment **E**ffects and screening **R**ecommendations). The ultimate goal of the survivorship care program is to improve life expectancy and quality of life of Hodgkin lymphoma survivors by reducing morbidity and mortality from late treatment effects. *(Nijdam A, et al. Br J Haematol. 2019;186(4):e103); Dekker N, et al. Ned Tijdschr Geneeskd. 2015;159:A9269.)* Together with her clinical colleagues she obtained a Dutch Cancer Society infrastructure grant to establish the introduction of the survivorship care program in a large number of hospitals; this grant was successfully completed in 2019. National evidence-based follow-up guidelines for HL survivors have been developed. So far, 18 BETER survivorship clinics have started and >8,500 eligible 5-year Hodgkin lymphoma and large B-cell lymphoma survivors have been identified. Lymphoma treatment data have been collected for all Hodgkin lymphoma survivors, to allow risk-based screening. These data are now being combined with BETER adverse events data in a national database to facilitate evaluation of the survivorship care program as well as research. Furthermore, together with the Lymphoma Patient Association Hematon we developed a special website about Hodgkin lymphoma late effects: www.beternahodgkin.nl. The proposed project will build upon the excellent nationwide collaboration of lymphoma treatment centres in the BETER consortium.
Flora van Leeuwen is also involved in the survivorship care program for Dutch survivors of childhood cancer (member of the Steering Group of LATER). She is also involved in many international collaborations regarding cancer survivorship care, e.g. the U.S. Childhood Cancer Survivor Study (member of the External Advisory Committee), the St. Jude Life study and with the UK investigators (Prof. John Radford, Manchester) planning to set up an infrastructure for Hodgkin lymphoma survivorship care in the UK.

Literature Search - Research Question

What is effectiveness of cancer survivorship care programs for survivors of lymphoma or childhood cancer?

Search Strategy

Databases of Pubmed, Embase (Ovid) and Scopus were searched for relevant publications up to February 19, 2020. The following search strategies were used:

Hodgkin

PubMed:
((survivor*[tiab] OR late effect*[tiab] OR long term adverse effect*[tiab]) AND (program*[tiab] OR surveillance[tiab] OR screening[tiab])) AND ("Hodgkin Disease"[Mesh] OR hodgkin*[tiab])

- 315 results

Embase(ovid):
(((survivor* OR "late effect*" OR "long term adverse effect*").ti,ab,kw. ADJ5 (program* OR surveillance OR screening).ti,ab,kw.) AND (exp Hodgkin disease/ OR (hodgkin*).ti,ab,kw.))

- 194 results

SCOPUS:
((TITLE-ABS-KEY (survivor* OR “late effect*” OR “long term adverse effect*”) W/5 TITLE-ABS-KEY(program* OR surveillance OR screening)) AND TITLE-ABS-KEY (hodgkin*))

- 130 results

Childhood:

PubMed:
(((survivor care[tiab] OR survivorship care[tiab]) AND (program*[tiab] OR surveillance[tiab] OR screening[tiab]) OR survivor program*[tiab])) AND (("Child"[Mesh] OR "Adolescent"[Mesh] OR "Infant"[Mesh] OR childhood[tiab] OR pediatric[tiab] OR paediatric[tiab]) AND ("Neoplasms"[Mesh] OR neoplas* [tiab] OR tumor [tiab] OR tumors [tiab] OR tumour* [tiab] OR cancer* [tiab] OR malign* [tiab] OR oncolog* [tiab] OR carcinom* [tiab]))

- 98 results

Embase(ovid):
(((("survivor care" OR "survivorship care").ti,ab,kw. ADJ5 (program* OR surveillance OR screening).ti,ab,kw.) OR ("survivor program*").ti,ab,kw.) AND ((childhood OR pediatric OR paediatric).ti,ab,kw. AND (neoplas* OR tumor OR tumors OR tumour* OR cancer* OR malign* OR oncolog* OR carcinom*).ti,ab,kw.))

- 45 results

SCOPUS:
(((TITLE-ABS-KEY("survivor care" OR "survivorship care") W/5 TITLE-ABS-KEY(program* OR surveillance OR screening)) OR TITLE-ABS-KEY("survivor program*")) AND (TITLE-ABS-KEY(childhood OR pediatric OR paediatric) AND TITLE-ABS-KEY(neoplas* OR tumor OR tumors OR tumour* OR cancer* OR malign* OR oncolog* OR carcinom*)))

- 33 results

Numbers FLOW chart

Database		Aantallen
PubMed	Hodgkin	315
**PubMed**	Childhood cancer	98
Embase(ovid)	Hodgkin	194
**Embase (ovid)**	Childhood cancer	45
SCOPUS	Hodgkin	130
**SCOPUS**	Childhood cancer	33
	Total	815
	Duplicates removed	269
	Total without duplicates	546
